# Specific associations between plasma biomarkers and postmortem amyloid plaque and tau tangle loads

**DOI:** 10.15252/emmm.202217123

**Published:** 2023-03-13

**Authors:** Gemma Salvadó, Rik Ossenkoppele, Nicholas J Ashton, Thomas G Beach, Geidy E Serrano, Eric M Reiman, Henrik Zetterberg, Niklas Mattsson‐Carlgren, Shorena Janelidze, Kaj Blennow, Oskar Hansson

**Affiliations:** ^1^ Clinical Memory Research Unit, Department of Clinical Sciences, Malmö Lund University Lund Sweden; ^2^ Alzheimer Center Amsterdam, Neurology Vrije Universiteit Amsterdam, Amsterdam UMC location VUmc Amsterdam The Netherlands; ^3^ Amsterdam Neuroscience, Neurodegeneration Amsterdam The Netherlands; ^4^ Department of Psychiatry and Neurochemistry, Institute of Neuroscience and Physiology, The Sahlgrenska Academy University of Gothenburg Gothenburg Sweden; ^5^ Institute of Psychiatry, Psychology and Neuroscience, Maurice Wohl Institute Clinical Neuroscience Institute King's College London London UK; ^6^ NIHR Biomedical Research Centre for Mental Health and Biomedical Research Unit for Dementia at South London and Maudsley, NHS Foundation London UK; ^7^ Banner Sun Health Research Institute Sun City AZ USA; ^8^ Banner Alzheimer's Institute Arizona State University and University of Arizona Phoenix AZ USA; ^9^ Clinical Neurochemistry Laboratory Sahlgrenska University Hospital Mölndal Sweden; ^10^ Department of Neurodegenerative Disease UCL Institute of Neurology, Queen Square London UK; ^11^ UK Dementia Research Institute at UCL London UK; ^12^ Hong Kong Center for Neurodegenerative Diseases Hong Kong China; ^13^ Department of Neurology Skåne University Hospital Lund Sweden; ^14^ Wallenberg Center for Molecular Medicine Lund University Lund Sweden; ^15^ Memory Clinic, Skåne University Hospital Malmö Sweden

**Keywords:** Alzheimer's disease, co‐pathologies, head‐to‐head, neuropathology, p‐tau species, Biomarkers, Neuroscience

## Abstract

Several promising plasma biomarkers for Alzheimer's disease have been recently developed, but their neuropathological correlates have not yet been fully determined. To investigate and compare independent associations between multiple plasma biomarkers (p‐tau181, p‐tau217, p‐tau231, Aβ42/40, GFAP, and NfL) and neuropathologic measures of amyloid and tau, we included 105 participants from the Arizona Study of Aging and Neurodegenerative Disorders (AZSAND) with antemortem plasma samples and a postmortem neuropathological exam, 48 of whom had longitudinal p‐tau217 and p‐tau181. When simultaneously including plaque and tangle loads, the Aβ42/40 ratio and p‐tau231 were only associated with plaques (ρ_Aβ42/40_[95%CI] = −0.53[−0.65, −0.35], ρ_p‐tau231_[95%CI] = 0.28[0.10, 0.43]), GFAP was only associated with tangles (ρ_GFAP_[95%CI] = 0.39[0.17, 0.57]), and p‐tau217 and p‐tau181 were associated with both plaques (ρ_p‐tau217_[95%CI] = 0.40[0.21, 0.56], ρ_p‐tau181_[95%CI] = 0.36[0.15, 0.50]) and tangles (ρ_p‐tau217_[95%CI] = 0.52[0.34, 0.66]; ρ_p‐tau181_[95%CI] = 0.36[0.17, 0.52]). A model combining p‐tau217 and the Aβ42/40 ratio showed the highest accuracy for predicting the presence of Alzheimer's disease neuropathological change (ADNC, AUC[95%CI] = 0.89[0.82, 0.96]) and plaque load (*R* ^2^ = 0.55), while p‐tau217 alone was optimal for predicting tangle load (*R* ^2^ = 0.45). Our results suggest that high‐performing assays of plasma p‐tau217 and Aβ42/40 might be an optimal combination to assess Alzheimer's‐related pathology *in vivo*.

## Graphical abstract

This study conducted a head‐to‐head comparison between multiple plasma biomarkers and neuropathological measures of amyloid plaques and neurofibrillary tangles.

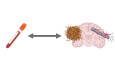

**The paper explained**

**Problem**

Alzheimer's disease is characterized by the deposition of amyloid plaques and neurofibrillary tau tangles in the brain. In recent years, several plasma biomarkers have been developed to assess these two pathologies, which have been validated against other fluid and neuroimaging biomarkers. However, their specific associations with each of these two pathologies are still not fully understood.

**Results**

In a set of participants with available blood samples and a neuropathological exam, we observed that some plasma biomarkers are specifically associated with only amyloid (Aβ42/40 and p‐tau231), some to only tau (GFAP) and, some to both pathologies (p‐tau217 and p‐tau181). Further, we showed that the combination of p‐tau217 and the Aβ42/40 ratio was optimal for assessing amyloid, while p‐tau217 alone was sufficient to assess tau pathology.

**Impact**

Comparing head‐to‐head the associations between high‐performing assays of different plasma biomarkers and neuropathological correlates allowed us to determine which is the optimal single or combination of plasma biomarkers for assessing actual pathology. This has a direct impact on the design of clinical trials, as we showed that p‐tau217 may not only be useful as a prescreening tool for clinical trials but also may be a good surrogate endpoint, especially on those trials targeting tau pathology. Furthermore, combining it with the Aβ42/40 ratio would significantly improve the assessment of continuous amyloid pathology.

## Introduction

The recent development of plasma biomarkers for Alzheimer's disease (AD) has revolutionized the field (Zetterberg & Bendlin, 2020; Hansson, 2021; Ossenkoppele *et al*, 2022), as these markers have the benefit of being significantly cheaper and less invasive than established markers (i.e., cerebrospinal fluid [CSF] and positron emission tomography [PET]), while showing the excellent diagnostic performance (Karikari *et al*, 2020; Palmqvist *et al*, 2020; Janelidze *et al*, 2021a). Several plasma biomarkers are currently available, among which, the most studied include the amyloid‐β42/40 (Aβ42/40) ratio, glial fibrillary acidic protein (GFAP), neurofilament light (NfL), and, particularly, phosphorylated tau (p‐tau) measures. Previous studies have indicated the excellent diagnostic performance of some of these plasma biomarkers for distinguishing AD from non‐AD neurodegenerative disorders (Karikari *et al*, 2020; Palmqvist *et al*, 2020; Thijssen *et al*, 2020; Janelidze *et al*, 2020a), with noninferior performance compared with CSF and PET markers (Palmqvist *et al*, 2019; Janelidze *et al*, 2020a, 2021a; Ashton *et al*, 2021; Benedet *et al*, 2021; Mielke *et al*, 2021), as well as an important utility for predicting disease progression (Ashton *et al*, 2021; Cullen *et al*, 2021; Mielke *et al*, 2021; Janelidze *et al*, 2021a). Nonetheless, there are still important topics to be addressed to optimize their usage in clinical practice, including improved interpretation of obtained plasma biomarker results and a fair head‐to‐head comparison, especially against gold standard neuropathological measures (Hansson *et al*, 2022).

One of the most important knowledge voids of plasma biomarkers is the degree to which they specifically correlate with key neuropathological changes. Although previous studies have already investigated the association of some of these biomarkers with measures of neuropathology, whether these markers are primarily related to amyloid, tau, or to both pathologies is still under debate. For instance, p‐tau181 has shown strong associations with neuropathological measures of amyloid‐β and tau pathologies (Lantero Rodriguez *et al*, 2020; Thijssen *et al*, 2020; Grothe *et al*, 2021; Smirnov *et al*, 2022), but this has been shown in independent analyses for amyloid and tau or with scales combining these two pathologies, which did not allow for the interpretation of the specific ‐or independent‐ associations with these two pathological measures. Similarly, plasma p‐tau231 has also shown associations with neuropathologically defined plaque and tangle load, without exploring specific associations with these neuropathologic measures (Ashton *et al*, 2021; Smirnov *et al*, 2022). Only one study with plasma p‐tau217 has suggested that this biomarker may be independently associated with both plaques and tangles (Mattsson‐Carlgren *et al*, 2021), but no other biomarkers were investigated. On the contrary, the accuracy of the plasma Aβ42/40 ratio, GFAP, or NfL levels to predict AD pathology seems to be lower than that of p‐tau markers, although only few studies have investigated their association with neuropathologic measures of AD pathology (Thijssen *et al*, 2020; Smirnov *et al*, 2022; Winder *et al*, 2022).

Another challenge when trying to optimize the use of plasma biomarkers in clinical practice is the lack of comparison among biomarkers in the same population. Differences in clinical performance for the same biomarkers can be observed depending on the characteristics of the study (e.g., diagnostic groups, patient characteristics, outcomes, and/or presence of co‐pathologies). Thus, head‐to‐head studies are crucial to allow a fair comparison and avoid bias due to population selection. Nonetheless, these studies are scarce, especially those including neuropathological measures (Smirnov *et al*, 2022; Winder *et al*, 2022). The use of neuropathological data would also allow investigating whether any of these biomarkers might be useful for detecting other common co‐pathologies observed in AD patients, such as Lewy bodies or TAR DNA‐binding protein 43 (TDP‐43; Hansson, 2021; Smirnov *et al*, 2022).

When comparing multiple plasma biomarkers, it is equally important to consider the discriminative power of the assays. While there are many plasma biomarkers currently available, there are also many platforms by which to measure them, which can highly affect their performance. As it has been shown recently with the plasma Aβ42/40 ratio, different assays and/or platforms could lead to significantly different performances in detecting AD‐related pathology (Janelidze *et al*, 2021b). Similarly, comparisons between multiple species and assays of p‐tau measures showed only a modest correlation, suggesting also significantly different diagnostic performance (Mielke *et al*, 2021; Janelidze *et al*, 2022a; Ashton *et al*, 2022b). Considering the differences in the clinical performance of different assays for the same biomarkers, the use of high‐performing assays is of utmost importance when comparing different biomarkers to avoid reporting differences that are related to the method rather than the biomarkers themselves.

Therefore, the main objective of this study was to identify specific relationships between multiple plasma biomarkers and core AD‐related pathologies using high‐performing assays. To this end, we investigated associations between multiple plasma biomarkers and autopsy‐assessed measures of core AD pathologies (plaque and tangle loads) in the same participants. We focused on investigating whether these biomarkers primarily reflect amyloid, tau, or both pathologies. Further, we identified the best combination of biomarkers to predict each of these pathological measures, as well as the presence or absence of AD as a binary measure based on pathology (Montine *et al*, 2012). We also investigated associations between plasma biomarkers and the presence of co‐pathologies commonly observed in AD patients including cerebral amyloid angiopathy (CAA), Lewy body disease (LBD), TDP‐43, cerebral white matter rarefaction (CWMR), and argyrophilic grain disease (AGD). Finally, we examined whether longitudinal changes of the two plasma biomarkers longitudinally available (i.e., p‐tau217 and p‐tau181) were associated with presence of AD pathology.

## Results

Our sample comprised a total of 105 participants from the Arizona Study of Aging and Neurodegenerative Disorders (AZSAND) including all participants with complete antemortem plasma samples and a postmortem neuropathological exam (Table 1). These participants were categorized as having significant AD pathologic change (*n* = 59) or not (*n* = 46) based on the Alzheimer's disease neuropathologic change (ADNC) scale, in which both amyloid and tau pathologies are accounted for (Montine *et al*, 2012). Participants with significant AD pathology were those with intermediate or high scores in the ADNC scale, whereas those with none or low scores were classified as having nonsignificant AD pathology. No differences in age at death nor sex were observed between groups. *APOE‐ε4* prevalence (49.2% vs. 10.9%, *P* < 0.001) and core AD pathology (plaques: 12.70 vs. 1.03, *P* < 0.001; tangles: 9.79 vs. 5.54, *P* < 0.001) measures were significantly higher in participants with intermediate/high ADNC. Among all the co‐pathologies under investigation (i.e., CAA, LBD, CWMR, and AGD), only the presence of CAA was significantly higher in participants with intermediate/high ADNC (86.4% vs. 34.8%, *P* < 0.001).

**Table 1:** Demographic characteristics at time of death.

	Overall (*n* = 105)	None/low ADNC (*n* = 46)	Interm./high ADNC (*n* = 59)	*P*‐value
*Demographics*				
Age, mean (SD)	84.7 (7.99)	83.5 (7.99)	85.7 (7.93)	0.174
Women, *n* (%)	43 (41.0%)	21 (45.7%)	22 (37.3%)	0.506
APOE‐e4 carrier, *n* (%)	34 (32.4%)	5 (10.9%)	29 (49.2%)	< 0.001
Time between blood sampling and death, days, mean (SD) (range)	482 (355) [9–1,760]	414 (300) [9–1,120]	536 (387) [9–1760]	0.137
*AD‐core neuropathological measures*				
Plaque total, mean (SD)	7.60 (6.34)	1.03 (1.99)	12.7 (2.83)	< 0.001
CERAD, *n* (%)				
Zero	27 (25.7%)	27 (58.7%)	0 (0%)	< 0.001
Sparse	19 (18.1%)	18 (39.1%)	1 (1.7%)	
Moderate	0 (0%)	0 (0%)	0 (0%)	
Frequent	59 (56.2%)	1 (2.2%)	58 (98.3%)	
Tangle total, mean (SD)	7.93 (3.69)	5.54 (2.41)	9.79 (3.44)	< 0.001
Braak stage, *n* (%)				
I–II	5 (4.8%)	5 (10.9%)	0 (0%)	< 0.001
III–IV	65 (61.9%)	37 (80.4%)	28 (47.5%)	
V–VI	35 (33.3%)	4 (8.7%)	31 (52.5%)	
*Presence of co‐pathologies*				
CAA, *n* (%)	67 (63.8%)	16 (34.8%)	51 (86.4%)	< 0.001
LBD, *n* (%)	21 (20.0%)	11 (23.9%)	10 (16.9%)	0.523
TDP‐43, *n* (%)^a^	19 (18.1%)	5 (10.9%)	14 (23.7%)	0.422
CWMR, *n* (%)	58 (55.2%)	20 (43.5%)	38 (64.4%)	0.052
AGD, *n* (%)^b^	28 (26.7%)	12 (26.1%)	16 (27.1%)	1.000
*Plasma levels*				
p‐tau217, pg/ml	0.432 (0.394)	0.194 (0.113)	0.617 (0.434)	< 0.001
p‐tau181, pg/ml	2.36 (1.49)	1.61 (0.845)	2.96 (1.61)	< 0.001
p‐tau231, pg/ml	30.0 (15.2)	25.4 (13.6)	33.6 (15.5)	< 0.001
Aβ42/40	0.125 (0.0142)	0.133 (0.0135)	0.119 (0.0114)	< 0.001
GFAP, ng/ml	0.190 (0.110)	0.149 (0.0948)	0.222 (0.111)	< 0.001
NfL, pg/ml	7.37 (5.11)	7.17 (5.12)	7.53 (5.15)	0.420

ADNC (Montine *et al*, 2012) refers to a measure of AD pathology and was dichotomized as: nonsignificant AD pathology (none/low) and significant AD pathology (intermediate/high). Plaques and tangles were measured in a semi‐quantitative scale ranging from 0 to 15. CERAD (Mirra *et al*, 1991) scale and Braak staging (Braak & Braak, 1991) refer to measures of plaque and neurofibrillary tangle, respectively. CAA (Beach *et al*, 2015), LBD (Beach *et al*, 2009), TDP‐43 (Arnold *et al*, 2013), CWMR (Dugger *et al*, 2014), and AGD (Josephs *et al*, 2008; Sabbagh *et al*, 2009) refer to the presence (vs. absence) of these co‐pathologies.

Aβ, amyloid‐β; ADNC, Alzheimer's disease neuropathologic change; AGD, argyrophilic grains disease; CAA, cerebral amyloid angiopathy; CERAD, Consortium to Establish a Registry for Alzheimer's Disease; CWMR, cerebral white matter rarefaction; GFAP, glial fibrillary acidic protein; Interm., intermediate; LBD, Lewy body disease; NfL, neurofilament light; p‐tau, phosphorylated tau; TDP‐43, TAR DNA‐binding protein 43.

^a^ Forty‐seven participants missing.

^b^ One participant missing.

### Associations between plasma biomarkers and core AD pathologies

**Figure 1:**
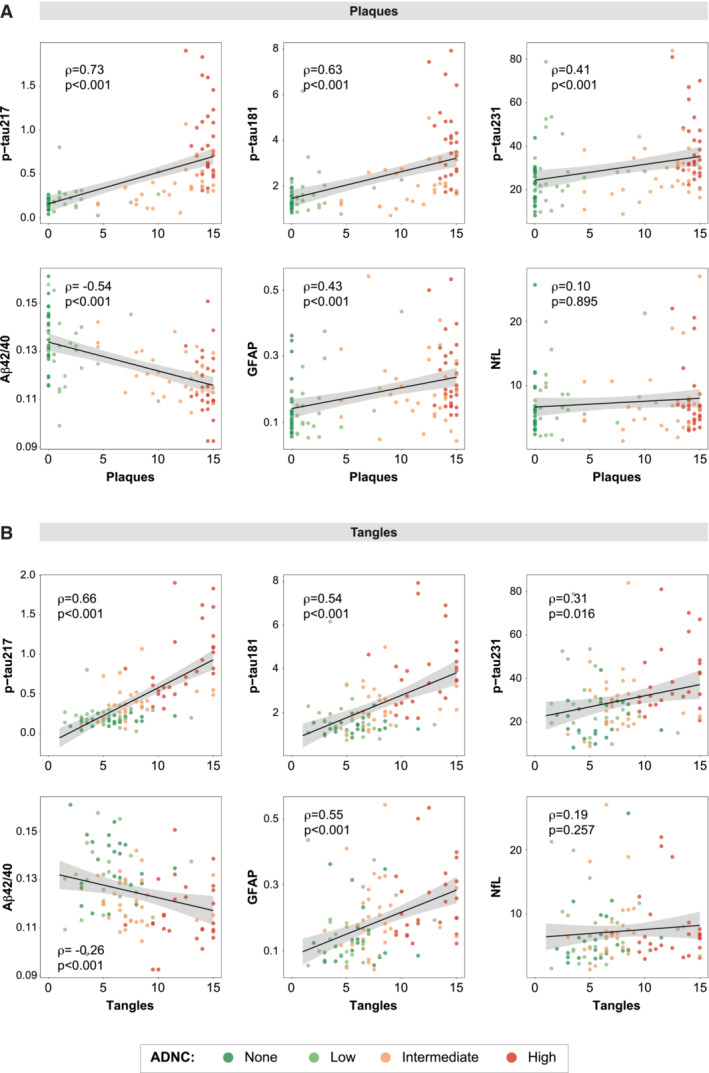
Associations between plasma biomarkers and amyloid plaque or neurofibrillary tau tangle loads Black lines represent the association between plasma biomarkers and amyloid plaque (A) and tau tangle (B) loads after adjusting for covariates (age, sex, and time between blood sampling and death), but dots represent raw data. Shadowed area represents the 95%CI. Plaque and tangle loads were measured on a semi‐quantitative scale from 0 to 3 using the CERAD (Mirra *et al*, 1991) templates in five different regions that were added up to a total score ranging from 0 to 15. Datapoints are colored based on the ADNC classification. Standardized Spearman's ρ and *P*‐values of the association between plasma biomarkers and a load of amyloid plaques or tau tangles are shown in the plot. Aβ, amyloid‐β; ADNC, Alzheimer's disease neuropathologic change; CERAD, Consortium to Establish a Registry for Alzheimer's Disease; CI, confidence interval; GFAP, glial fibrillary acidic protein; NfL, neurofilament light; p‐tau, phosphorylated tau.

Our first objective was to assess the associations between each plasma biomarker and the two neuropathological measures of AD pathology (i.e., total amount of plaques and tangles), independently. Plaque and tangle loads were measured on a semi‐quantitative scale that ranged from 0 to 3 in five different brain regions (Mirra *et al*, 1991), and we combined these regional measures into a total score (range: 0–15) for each pathology. We used partial Spearman's ρ to assess the association between each plasma biomarker and each total amount of pathology while adjusting for age, sex, and time between blood draw and death. We found that all plasma biomarkers except NfL (ρ = 0.10, *P* = 0.895) were significantly associated with the total amount of plaques (0.41 ≤ |ρ| ≤ 0.73, *P* < 0.001, Fig 1A and Table 2). Plasma p‐tau217 showed the highest correlation coefficient with plaques, which was significantly higher than all others (0.10 ≤ ρ_diff_ ≤ 0.63, *P* ≤ 0.016) except plasma Aβ42/40 ratio (ρ_diff_ = 0.19, *P* = 0.055). All plasma biomarkers except NfL (ρ = 0.19, *P* = 0.257) were also associated with the total amount of tangles in the independent models (0.26 ≤ |ρ| ≤ 0.66, *P* ≤ 0.016; Fig 1B and Table 2). Again, p‐tau217 had the highest correlation coefficient with the total amount of tangles, which was significantly higher than all other plasma correlation coefficients (0.11 ≤ ρ_diff_ ≤ 0.47, *P* ≤ 0.006) except for that of plasma GFAP (ρ_diff_ = 0.10, *P* = 0.206).

**Table 2:** Associations between plasma biomarkers and amyloid plaque and/or neurofibrillary tau tangle loads.

	Adjusted for covariates			Adjusted for covariates and pathology		
	ρ [95%CI]	*P*‐value	*P*‐value comp.	ρ [95%CI]	*P*‐value	*P*‐value comp.
*Plaques*						
p‐tau217	0.73 [0.58, 0.8]	**< 0.001**	Ref.	0.40 [0.21, 0.57]	**0.003**	0.246
p‐tau181	0.63 [0.47, 0.72]	**< 0.001**	0.016	0.36 [0.16, 0.52]	**0.009**	0.146
p‐tau231	0.41 [0.23, 0.54]	**< 0.001**	< 0.001	0.28 [0.09, 0.44]	0.084	0.028
A**β**42/40	−0.54 [−0.68, −0.37]	**< 0.001**	0.055	−0.53 [−0.66, −0.36]	**< 0.001**	Ref.
GFAP	0.43 [0.24, 0.58]	**< 0.001**	0.001	0.09 [−0.11, 0.31]	1.000	< 0.001
NfL	0.10 [−0.09, 0.28]	0.895	< 0.001	−0.04 [−0.25, 0.19]	0.145	< 0.001
*Tangles*						
p‐tau217	0.66 [0.51, 0.78]	**< 0.001**	Ref.	0.52 [0.34, 0.66]	**< 0.001**	Ref.
p‐tau181	0.54 [0.36, 0.67]	**< 0.001**	0.006	0.36 [0.15, 0.5]	**0.010**	0.004
p‐tau231	0.31 [0.10, 0.48]	**0.016**	0.001	0.11 [−0.09, 0.31]	1.000	< 0.001
A**β**42/40	−0.26 [−0.45, −0.05]	**< 0.001**	< 0.001	0.11 [−0.12, 0.32]	1.000	< 0.001
GFAP	0.55 [0.38, 0.68]	**< 0.001**	0.206	0.39 [0.18, 0.58]	**0.004**	0.207
NfL	0.19 [−0.01, 0.38]	0.257	< 0.001	0.19 [−0.04, 0.36]	0.535	0.003

Partial Spearman's ρ was used to investigate associations between plasma biomarkers and AD‐core pathologies. In all cases, we corrected for age, sex, and time between blood sampling and death. We further corrected for tau (first set of rows) or amyloid (last set of rows) pathologies for assessing specific associations with amyloid and tau, respectively (last three columns). Semi‐quantitative amyloid plaque load was used as a predictor in the first set of analyses (first set of rows) and semi‐quantitative tau tangle load was used in the second set of analyses (last set of rows). Significant associations (*P* < 0.05 corrected for multiple comparisons) are shown in bold. Differences between the correlation coefficients were tested using bootstrapping the strongest association as reference (Ref.) and shown in the last column. Significant differences (*P* < 0.05) can be understood as significantly weaker associations compared with those of the references in each case.

Aβ, amyloid‐β; CI, confidence intervals; GFAP, glial fibrillary acidic protein; NfL, neurofilament light; p‐tau, phosphorylated tau.

As a sensitivity analysis, we also investigated these correlations separately for participants without (i.e., ADNC none or low) and with significant AD pathology (i.e., ADNC intermediate or high, Appendix Table S1). In the group without significant AD pathology, only the Aβ42/40 ratio showed a significant correlation with amyloid (ρ = ‐0.33, *P* < 0.001). No plasma biomarkers showed a significant correlation with tau tangle load in this group. In the group of significant AD pathology, both p‐tau217 (ρ = 0.41, *P* = 0.049) and the Aβ42/40 ratio (ρ = ‐0.30, *P* < 0.001) presented a significant correlation with amyloid plaque load. Further, p‐tau217 (ρ = 0.56, *P* = 0.001), p‐tau181 (ρ = 0.49, *P* = 0.008), and GFAP (ρ = 0.47, *P* = 0.011) had a significant correlation with tau tangle load.

**Figure 2:**
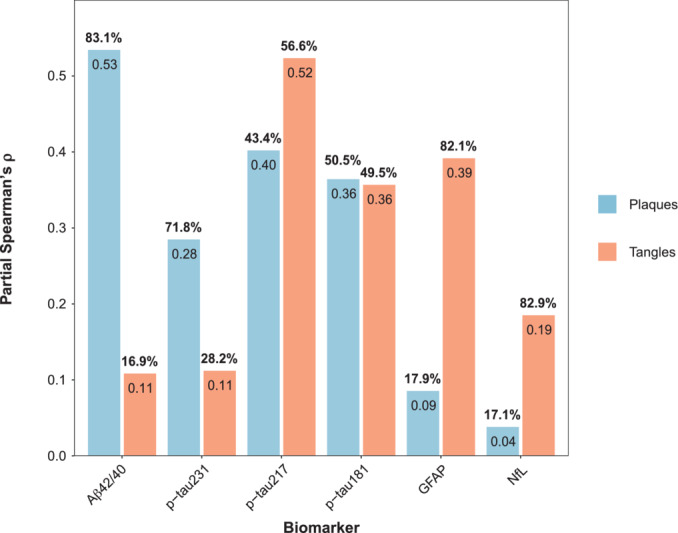
Specific associations between plasma levels and both amyloid plaque and tau tangle loads Bars represent the partial Spearman's ρ of amyloid plaque load (blue) and tau tangle load (orange) on plasma levels after adjusting for the other pathology load. In separate models, each biomarker was used as a dependent variable and either amyloid load or tau load as independent variables adjusting for the other pathology measure. We also adjusted for age, sex, and time between blood sampling and death. Numbers inside the bars represent partial Spearman's ρ and numbers above the bars represent the percentual partial Spearman's ρ over the sum of the partial Spearman's ρ of the two pathologies' (%partial ρ = 100*partial ρ/[partial ρ_plaque_ + partial ρ_tangle_]). Aβ, amyloid‐β; GFAP, glial fibrillary acidic protein; NfL, neurofilament light; p‐tau, phosphorylated tau.

Since plaque and tangle load were highly correlated (ρ[95%CI] = 0.63[0.48, 0.73], *P* < 0.001, Appendix Fig S1), we performed an analysis to identify the specific (or independent) associations between each plasma biomarker and the two pathologies. For this, we used partial Spearman's ρ again, adjusting further for the other pathology as well (i.e., when looking at plaques adjusting for tangles and vice‐versa). In these models, p‐tau217 (ρ = 0.40, *P* = 0.003), p‐tau181 (ρ = 0.36, *P* = 0.009), and the Aβ42/40 ratio (ρ = ‐0.53, *P* < 0.001) were significantly associated with plaques (Fig 2 and Table 2). Plasma GFAP showed no significant association with plaque load when adjusted for tangle load (β = 0.09, *P* = 1.00) and p‐tau231 showed an association only at a trend level (ρ = 0.28, *P* = 0.084). In this analysis, the Aβ42/40 ratio correlation coefficient with plaques was the highest, being significantly higher than that of p‐tau231 (ρ_diff_ = 0.25, *P* = 0.028) but not than that of p‐tau217 (ρ_diff_ = 0.13, *P* = 0.246) nor p‐tau181 (ρ_diff_ = 0.17, *P* = 0.146). In addition, we observed that only p‐tau217 (ρ = 0.52, *P* < 0.001), p‐tau181 (ρ = 0.36, *P* = 0.010), and GFAP (ρ = 0.39, *P* = 0.004) were associated with tangles. The correlation coefficient of p‐tau217 with tangles was significantly higher than that of p‐tau181 (ρ_diff_ = 0.17, *P* = 0.004) when adjusting for the total amount of plaques but not than that of plasma GFAP (ρ_diff_ = 0.13, *P* = 0.207).

Comparing the correlation coefficients to each of these two pathologies for each biomarker, we observed three groups of biomarkers (Fig 2 and Appendix Table S2). We observed that plasma p‐tau231 (71.8%) and the Aβ42/40 ratio (83.1%) had a major proportion of variance explained by plaques than tangles. On the contrary, the opposite happened with plasma GFAP and NfL, with tangles explaining the major part of these biomarkers' variance (GFAP: 82.1%, NfL: 82.9%). Finally, in p‐tau217 (plaques: 43.4%, tangles: 56.6%) and p‐tau181 (plaques: 50.5%, tangles: 49.5%) both pathologies contributed similarly to explaining their variance.

Finally, we investigated which combination of biomarkers better‐predicted plaques and tangles, independently. We found that the parsimonious model that better‐predicted load of amyloid plaques included both p‐tau217 and the Aβ42/40 ratio (*R* ^2^ = 0.57, Table 3), which was significantly better than the one only including p‐tau217 based on AICc (ΔAICc = 15.5). On the contrary, p‐tau217 alone was selected as the parsimonious model to predict the load of neurofibrillary tangles (*R* ^2^ = 0.50, Table 3).

**Table 3:** Parsimonious models to predict AD‐related pathology.

	β [95%CI]	*P*‐value association	*R* ^2^	AICc	AUC [95%CI]
*Plaques*					
p‐tau217	0.58 [0.44, 0.72]	**< 0.001**	0.57	218.36	–
A**β**42/40	−0.32 [−0.46, −0.18]	**< 0.001**			
Age	0.03 [−0.11, 0.16]	0.705			
Sex	0.01 [−0.26, 0.28]	0.940			
Time blood‐death	0.00 [−0.14, 0.13]	0.968			
*Tangles*					
p‐tau217	0.64 [0.50, 0.78]	**< 0.001**	0.50	233.46	–
Age	0.02 [−0.12, 0.16]	0.776			
Sex	−0.34 [−0.63, −0.05]	**0.020**			
Time blood‐death	0.13 [−0.01, 0.27]	0.077			
*ADNC*					
p‐tau217	1.83 [1.09, 2.79]	**< 0.001**	0.66	93.77	0.90 [0.84, 0.96]
A**β**42/40	−1.00 [−1.79, −0.29]	**0.008**			
Age	0.29 [−0.26, 0.89]	0.310			
Sex	0.07 [−1.10, 1.26]	0.908			
Time blood‐death	0.23 [−0.45, 0.96]	0.513			

Parsimonious models were selected as those that better explained each AD pathology measure with a smaller number of predictors based on the AICc criterion. Initial models included basic covariates (age, sex, and time between blood sampling) and all biomarkers that showed a significant association in the univariate analyses. Covariates were kept in the models even when they did not contribute to the model for a fair comparison to univariate analyses. Significant predictors (*P* < 0.05) are shown in bold. Men are the reference sex group.

Aβ, amyloid‐β; ADNC, Alzheimer's disease neuropathologic change; AICc, corrected Akaike criterion; AUC, area under the curve; CI, confidence intervals; p‐tau, phosphorylated tau.

### Prediction of neuropathological scales' classification

**Figure 3:**
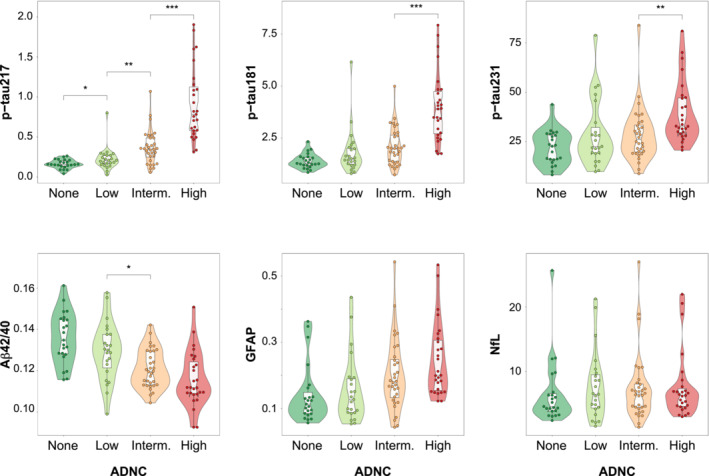
Plasma levels by ADNC classification Groups were compared using a pairwise Wilcoxon test as a *post hoc* comparison after testing tendency using a Kruskal–Wallis test. *Post hoc* comparisons were only performed between consecutive groups (*n*_comp_ = 3). Central band of the boxplot represents the median of the group, the lower and upper hinges correspond to the first and third quartiles, and the whiskers represent the maximum/minimum value or the 1.5 IQR from the hinge, whatever is lower. ***P ≤ 0.001; **P ≤ 0.010; *P ≤ 0.050. Aβ, amyloid‐β; ADNC, Alzheimer's disease neuropathologic change; CI, confidence interval; GFAP, glial fibrillary acidic protein; IQR, interquartile range; NfL, neurofilament light; p‐tau, phosphorylated tau.

Next, we investigated differences in plasma levels by ADNC groups (as a four‐level variable, i.e., none, low, intermediate, or high) using a Kruskal‐Wallis test and Wilcoxon test for *post hoc* comparisons. All three p‐tau plasma measures showed significant differences between intermediate and high levels of ADNC. Plasma p‐tau217 and the Aβ42/40 ratio levels were significantly different between intermediate and low ADNC. However, we only found significant differences between none and low ADNC in plasma p‐tau217 levels (Fig 3), although this became only a trend when removing the highest plasma value of the low group. Notably, p‐tau217 also showed the highest fold‐change among all ADNC consecutive levels (Appendix Table S3).

Then, we also examined differences in pathological scales specific for amyloid (Consortium to establish a registry for Alzheimer's disease [CERAD]) (Mirra *et al*, 1991) and tau (Braak staging) (Braak & Braak, 1991) pathologies. Similarly, all biomarkers except NfL showed significant differences between sparse and moderate/frequent groups on CERAD's classification. Only plasma p‐tau217 showed differences between zero and sparse groups (Appendix Fig S2). Regarding Braak staging, all biomarkers except NfL were significantly different when comparing 0–IV with V–VI groups (Appendix Fig S3).

**Figure 4:**
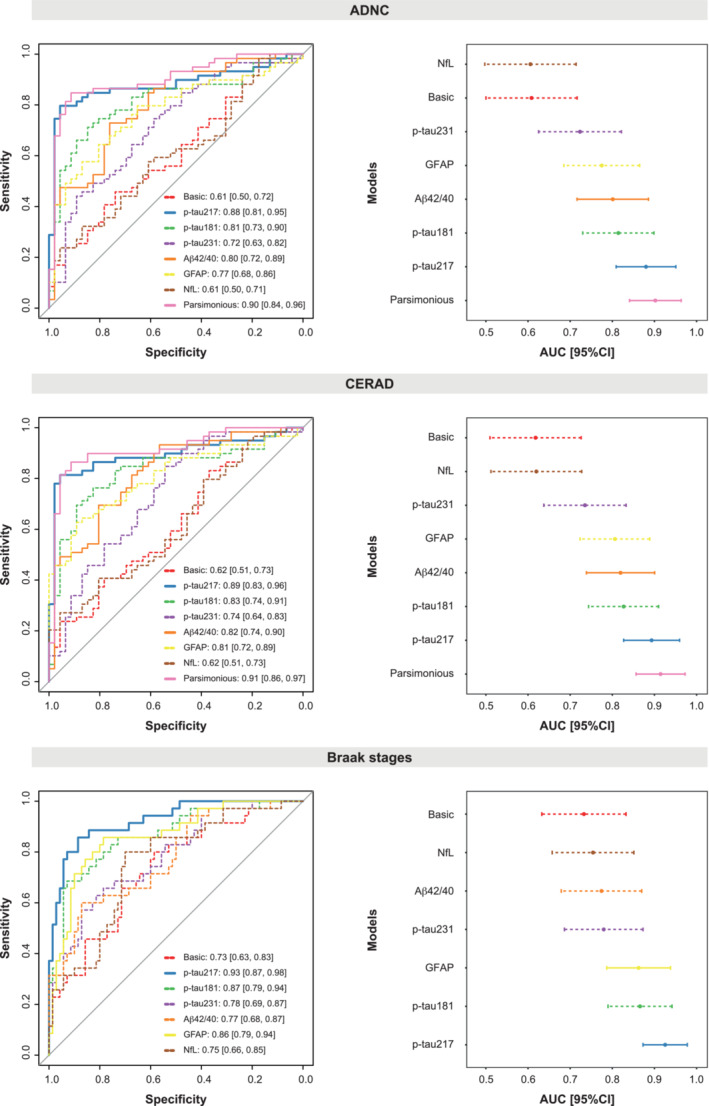
Plasma biomarkers for predicting neuropathological scales' classification ROC curves for all models are shown in the left column and the correspondent AUC and 95% CI are shown in the right column. Models for all individual plasma biomarkers and the parsimonious (when available) model are shown. All models included: age, sex, and time between blood sampling and death as covariates. The parsimonious model for ADNC and CERAD included p‐tau217 and Aβ42/40 as predictors. The basic model includes only covariates. ADNC was dichotomized as negative (none/low) or positive (intermediate/high). CERAD was dichotomized as negative (low/sparse) or positive (moderate/frequent). Braak stages were dichotomized as negative (0–IV) or positive (V–VI). The individual biomarker with the best performance is shown as a solid bold line. Dashed lines represent individual biomarkers with significant (*P* < 0.05) lower AUC than the best individual biomarker (p‐tau217 in all cases). Other models with solid lines represent AUC equivalent to that of the best individual biomarker. Aβ, amyloid‐β; ADNC, Alzheimer's disease neuropathologic change; AUC, area under the curve; CERAD, Consortium to Establish a Registry for Alzheimer's Disease; CI, confidence interval; GFAP, glial fibrillary acidic protein; NfL, neurofilament light; p‐tau, phosphorylated tau; ROC, receiver‐operating characteristic.

We next investigated the accuracy of each plasma biomarker to predict the presence of AD pathology as measured with the dichotomized ADNC (none/low vs. intermediate/high) classification. For this, we used receiver‐operating characteristic (ROC) curves and calculated the area under the curve (AUC) for each biomarker independently adjusting for age, sex, and time between blood sampling and death. All biomarkers except NfL (AUC[95%CI] = 0.61 [0.50, 0.71], *P* = 0.698) were predictive of the presence of ADNC when assessed individually (0.88 ≥ AUC ≥ 0.72, Appendix Table S4 and Fig 4). Plasma p‐tau217 had the highest AUC (AUC[95%CI] = 0.88 [0.81–0.95]) of all individual biomarkers, which was significantly higher than all others except for the Aβ42/40 ratio (AUC[95%CI] = 0.80 [0.72–0.89], *P* = 0.099). We also repeated this analysis with CERAD (low/sparse vs. moderate/frequent) and Braak staging (0–IV vs. V–VI) classification. For CERAD, p‐tau217 was also the best individual biomarker as per classification accuracy (AUC[95%CI] = 0.89 [0.83–0.96], *P* < 0.001), comparable only to that of the Aβ42/40 ratio (AUC[95%CI] = 0.82 [0.74–0.90], *P* < 0.001, Fig 4 and Appendix Table S5). For Braak staging, p‐tau217 again showed the highest accuracy (AUC[95%CI] = 0.93 [0.87–0.98], *P* < 0.001) comparable only to that of GFAP (AUC[95%CI] = 0.86[0.79–0.94], *P* < 0.001, Fig 4 and Appendix Table S6).

Next, we investigated whether combining different biomarkers would improve the models with only individual biomarkers. We found that plasma p‐tau217 and the Aβ42/40 ratio was the optimal combination to predict the presence of ADNC (AUC[95%CI] = 0.90 [0.84, 0.96], Table 3 and Fig 4), but the AUC was not significantly higher than that of p‐tau217 alone when using the DeLong's test (*P* = 0.124). With CERAD we observed a similar behavior, with plasma p‐tau217 and the Aβ42/40 ratio being the best combination (AUC[95%CI] = 0.91 [0.86–0.97], Appendix Table S7), although the AUC was not significantly better than that of p‐tau217 alone (*P* = 0.173). In the case of Braak staging, there were no combinations of biomarkers that improved the accuracy compared with p‐tau217‐only models.

### Prediction of presence of co‐pathologies

In this analysis, we investigated whether any of the available plasma biomarkers improved the basic models' accuracy (only covariates) on predicting the presence of co‐pathologies, using a similar approach as before but further adjusting for the presence of intermediate/high ADNC. We observed that only plasma NfL significantly improved the prediction of the presence of CWMR (Dugger *et al*, 2014) (AUC[95%CI] = 0.76 [0.66, 0.85]) compared with the basic model (AUC[95%CI] = 0.65 [0.54, 0.76], *P* = 0.028, Appendix Table S8 and Appendix Fig S4A). In particular, participants with CWMR had significantly higher plasma NfL levels than those without (β = 0.88, *P* = 0.002, Appendix Fig S4B). No other biomarkers improved the prediction of the presence of this nor any other co‐pathology (Appendix Tables S9–S12 and Appendix Fig S5). Raw distribution of plasma levels by the presence of each co‐pathology can be observed in Appendix Figs S6–S10.

As an additional analysis, we further checked whether there were differences between plasma levels in participants with only co‐pathologies (e.g., CAA only) and participants with AD pathology and co‐pathologies (e.g., CAA and ADNC) as it may have important clinical implications. We found that p‐tau217 was significantly higher in those participants having both AD pathology (as ADNC intermediate or high) and CAA compared to those with only AD pathology (*P* = 0.037, Appendix Fig S11). However, the group of AD‐only pathology was small (*n* = 8). At the statistical trend level, we also observed differences in plasma Aβ42/40 levels in AD‐only versus AD and LBD groups (*P* = 0.058, Appendix Fig S12); in both p‐tau217 and Aβ42/40 levels in AD‐only versus AD and AGD groups (*P* = 0.052 and *P* = 0.069, respectively; Appendix Fig S13) and in NfL levels in AD‐only versus AD and CWMR (*P* = 0.090, Appendix Fig S14). No differences were observed in the case of TDP‐43 (Appendix Fig S15). Finally, we also considered primary tauopathies (CBD, PSP, and AGD) as a unique group and compared the plasma levels of those participants to those with only AD pathology and those with AD pathology and other tauopathies. Plasma p‐tau217 (*P* < 0.001), p‐tau181 (*P* = 0.001), Aβ42/40 ratio (*P* < 0.001), and GFAP (*P* = 0.024) levels were significantly different when comparing participants with only AD pathology and participants with only primary tauopathies (Appendix Fig S16). Only Aβ42/40 ratio levels were different between the AD group with CBD, PSP, or AGD pathology (*P* = 0.038).

### Use of the p‐tau217/Aβ42 ratio

Given that the CSF p‐tau/Aβ42 ratio is commonly used both in research and in clinical practice, we wanted to investigate whether a plasma p‐tau/Aβ42 ratio would also be useful for predicting AD pathology. For this, we selected p‐tau217 as it showed the highest associations in the previous analyses. We compared the accuracy of predicting plaques and tangles, independently, comparing parsimonious models including the plasma p‐tau217/Aβ42 ratio as a possible independent variable to those obtained in the previous sections including p‐tau217. We observed that the p‐tau217/Aβ42 ratio was preferentially selected over p‐tau217 in the models predicting plaques and tangles. Based on the AICc, we observed that models including the p‐tau217/Aβ42 ratio were slightly, but significantly, better than those previously presented (plaques: *R* ^2^_p‐tau217/Aβ42 ratio_ = 0.60, AICc = 210.1 vs. *R* ^2^_p‐tau217_ = 0.57, AICc = 218.4; tangles: *R* ^2^_p‐tau217/Aβ42 ratio_ = 0.52, AICc = 228.7 vs. *R* ^2^_p‐tau217_ = 0.50, AICc = 233.5; Appendix Table S13). We also observed that p‐tau217/Aβ42 ratio levels were significantly different between the none versus low ADNC groups (*P* = 0.029), even when removing the outlier in the low group.

### Longitudinal associations between p‐tau217 and p‐tau181 with AD pathology

Finally, we investigated whether longitudinal changes in plasma p‐tau217 and p‐tau181 were associated with the presence of AD pathology at death (median[range] timepoints: 2 [2–5], mean (SD) time difference from first timepoint to death: 1378 (1357) days). Details of these participants can be found in Appendix Table S14. First, we observed that longitudinal increments of p‐tau217 but not p‐tau181 were associated with plaque burden (p‐tau217: β = 0.09, *P* = 0.005; p‐tau181: β = 0.05, *P* = 0.350, Table 4). In independent models, we observed that p‐tau217 increments, but not those in p‐tau181, were also associated with tangle load (p‐tau217: β = 0.09, *P* = 0.004; p‐tau181: β = 0.08, *P* = 0.094, Table 4).

**Table 4:** Associations between longitudinal changes of plasma biomarkers and the presence of AD‐related pathology at death.

Predictor	p‐tau217				p‐tau181			
	β [95%CI]	*P*	*R* ^2^	AICc	β [95%CI]	*P*	*R* ^2^	AICc
Plaques*time	0.09 [0.03, 0.15]	**0.005**	0.37	189.35	0.05 [−0.05, 0.15]	0.350	0.30	255.72
Tangles*time	0.09 [0.03, 0.14]	**0.004**	0.53	173.95	0.08 [−0.01, 0.17]	0.094	0.35	248.79
ADNC*time	0.13 [0.02, 0.24]	**0.018**	0.24	197.95	0.12 [−0.05, 0.29]	0.152	0.21	258.79

Linear mixed effect models were used to investigate these associations in independent models including age at baseline and sex as covariates using random intercepts and fixed time‐slopes. The interaction between time and amyloid plaques, tau tangles, or the presence of ADNC was used as predictors, in independent models for both p‐tau217 and p‐tau181. Significant associations (*P* < 0.05) between plasma biomarkers and the presence of AD‐related pathology are shown in bold.

ADNC, Alzheimer's disease neuropathologic change; AICc, corrected Akaike criterion, CI, confidence intervals; p‐tau, phosphorylated tau.

In the last analysis, we examined whether participants with intermediate/high ADNC pathology at death showed higher increments in p‐tau levels compared with those with none/low ADNC pathology. We observed that participants with intermediate/high ADNC had significantly higher p‐tau217, but not p‐tau181, longitudinal increases (p‐tau217: β = 0.13, *P* = 0.018; p‐tau181: β = 0.12, *P* = 0.152, Table 4 and Appendix Fig S17). These differences were observable up to 7 years before death, as defined by nonoverlapping 95%CIs. These results remained when removing two cases with very high plasma levels (p‐tau217: β = 0.21, *P* = 0.009; p‐tau181: β = 0.16, *P* = 0.118).

## Discussion

In this study, we have investigated the specific associations between multiple plasma biomarkers, using high‐performing assays, and autopsy‐assessed measures of AD pathology in a single cohort. Our main result was that the plasma Aβ42/40 ratio and p‐tau231 were selectively associated with plaques, plasma GFAP only with tangles, whereas p‐tau181 and, most strongly, p‐tau217 were independently associated with both plaques and tangles. We also observed that p‐tau217 showed the highest accuracy to predict the presence of AD pathology. Regarding co‐pathologies, only the use of plasma NfL showed an improvement on predicting the presence of cerebral white matter rarefaction (CWMR), but no other biomarkers further improved this prediction nor any of any other co‐pathology. Notably, the use of the plasma p‐tau217/Aβ42 ratio showed slight, although significant, improvements compared with p‐tau217 alone when assessing semi‐quantitative measures of AD pathology. Finally, we observed that longitudinal increases in p‐tau217, but not those of p‐tau181, were significantly associated with the presence of AD pathology at death, especially with tangle burden. Taken altogether, this study supports the use of plasma p‐tau217, when assessed with high‐performing assays, as the best biomarker for measuring AD‐related pathology, supported by its independent associations with neuropathological measures of both plaques and tangles.

The main result of this study was the observation that plasma p‐tau217 and plasma p‐tau181 were specific markers of both amyloid plaques and tau tangles. A previous study with a subsample of the individuals included here (*n* = 88) already suggested an independent association between plasma p‐tau217 and the two main AD‐related pathologies (Mattsson‐Carlgren *et al*, 2021). The novelty of our study was to demonstrate that this dual association only occurred in p‐tau217 and p‐tau181. Further, we observed that p‐tau217 changed earlier along the ADNC scale (Fig 3), and also that longitudinal changes in plasma p‐tau217, but not those of p‐tau181, were associated with AD‐related pathology. Although this analysis was exploratory, due to the limited sample size with longitudinal data, it is in agreement with a very recent study in which plasma p‐tau217 was the only biomarker with significantly different longitudinal increases based on amyloid status in both CU and MCI participants (Ashton *et al*, 2022a). Altogether, our data suggest that plasma p‐tau217 is the best‐suited plasma biomarker among the ones studied here to assess the presence of AD‐related pathology across the whole *continuum*. Although p‐tau181 has shown very good performance as an AD biomarker (Karikari *et al*, 2020, 2021; Thijssen *et al*, 2020; Janelidze *et al*, 2020a; Grothe *et al*, 2021), multiple (plasma and CSF) studies support that p‐tau217 may be a more useful biomarker than p‐tau181, as it has stronger correlations with amyloid and tau pathology proxies, earlier change, and better diagnostic accuracy (Barthélemy *et al*, 2020; Hanes *et al*, 2020; Palmqvist *et al*, 2020; Janelidze *et al*, 2020b, 2021a; Grothe *et al*, 2021; Leuzy *et al*, 2021). Further, our longitudinal results suggest that the utilization of plasma p‐tau217 in clinical trials may be useful not only as a prescreening method, but also for disease monitoring, especially for those drugs targeting tau pathology, but larger sample sizes are needed to confirm this finding.

While plasma p‐tau217 and p‐tau181 were associated with both plaques and tangles, the other studied biomarkers showed more specific associations to only one pathology. For instance, when amyloid was not accounted for, plasma p‐tau231 showed a significant correlation with tangle counts; however, when taking into account the two pathologies, this biomarker only showed a significant association with amyloid plaques. Previous studies have suggested that p‐tau231, both as a CSF and a plasma biomarker, may be an early AD marker tightly associated with amyloid pathology (Suárez‐calvet *et al*, 2020; Ashton *et al*, 2021; Meyer *et al*, 2022; Milà‐Alomà *et al*, 2022; Smirnov *et al*, 2022). Contrary to previous studies, we observed significantly lower associations with amyloid than that of p‐tau217 and the Aβ42/40 ratio, and some elevated levels in subjects without neuropathological evidence of amyloid plaques (Fig 1). One possible explanation for the early increases observed here and in previous studies may be that they are related to soluble amyloid, which cannot be detected in our study and is presumably an earlier event in the Alzheimer's *continuum*. We acknowledge that further research is needed to understand the relationship between this biomarker and actual pathology.

Similarly, the plasma Aβ42/40 ratio was also only associated with plaques when both pathologies were included in a single model, supporting its tight relationship with amyloid pathology (Verberk *et al*, 2018; Janelidze *et al*, 2021b). Nonetheless, the most important finding regarding the plasma Aβ42/40 ratio was that combining it with plasma p‐tau217 could slightly improve amyloid plaque assessment, replicating a previous result from our group when assessing amyloid positivity by CSF (Janelidze *et al*, 2022b); however, in our case this improvement only reached significance when predicting the continuous variable. Thus, our results suggest that the combination of the plasma Aβ42/40 ratio and p‐tau217 may be useful in clinical trials targeting amyloid pathology as a prescreening method, but more powered studies are needed to confirm the additional value of the Aβ42/40 ratio.

One surprising finding of our study was the specific association between plasma GFAP and tau tangles. Contrarily, previous studies have shown significant associations between this marker and amyloid pathology (as measured by CSF or PET), which were stronger than those with tau pathology (also measured with CSF or PET; Benedet *et al*, 2021; Pereira *et al*, 2021). Apart from the fact that GFAP levels were high in some cases with no or low amounts of plaques, two main points must be accounted when comparing these to our results. First, none of the aforementioned studies adjusted for tau pathology when assessing associations with amyloid. Following this approach (i.e., adjusting only for covariates), we also observed an association between plasma GFAP and plaques. And second, that tau PET is known to not be sensitive to early tau pathology, which may have decreased the power to detect these associations in previous studies (Mattsson *et al*, 2017; Leuzy *et al*, 2020; Soleimani‐meigooni *et al*, 2020). On the contrary, recent studies have shown the association between higher levels of plasma GFAP and increased risk of clinical progression and steeper rates of cognitive decline (even after adjusting for amyloid) (Rajan *et al*, 2020; Verberk *et al*, 2021; Ebenau *et al*, 2022), which supports a link with tau pathology given the known strong association between tau and clinical symptoms. Another plausible hypothesis is that plasma GFAP levels are not directly related to either plaque or tangle deposition but to the astrocytic reactivity in response to these processes. Actually, GFAP as a protein is overexpressed in reactive astrocytes, and its measures in CSF GFAP have been widely accepted as a marker of reactive astrogliosis. Unfortunately, no measures neuropathological measures of astrocytic reactivity were available in this sample, which prevented us to investigate this important issue. Future studies should investigate whether plasma GFAP is related to astrocytic reactivity and up to what level this is also indirectly related to amyloid and/or tau pathologies.

The CSF p‐tau/Aβ42 ratio has received a lot of attention in recent years, both as a research and a clinical tool (Hansson *et al*, 2006; Fagan *et al*, 2007; Li *et al*, 2007; Snider *et al*, 2009; Milà‐Alomà *et al*, 2020; Salvadó & Larsson, 2022). Thus, we wanted to investigate whether a similar ratio would be also useful using plasma biomarkers. We observed that p‐tau217/Aβ42 ratio slightly, but significantly, improved prediction accuracy to detect AD‐related pathology, and seem to be able to detect earlier changes. Although further investigation is needed, we suggest that this ratio may be useful to track AD pathology across the *continuum* due to its relationship with both main pathological hallmarks of AD, as well as better statistical characteristics of ratios, which can account for production/clearance participants' inter‐variability (Janelidze *et al*, 2016; Hansson *et al*, 2019).

Finally, we also investigated whether the levels of these biomarkers could be used to predict the presence of common AD co‐pathologies. Only plasma NfL significantly predicted the presence of CWMR (i.e., significantly improved the model with only covariates), with those subjects with the presence of CWMR having higher plasma NfL levels. This is in agreement with NfL being a biomarker of nonspecific axonal degeneration (Zetterberg *et al*, 2016; Bridel *et al*, 2019), but these results should be confirmed in an independent cohort. None of the biomarkers investigated could predict any of the other co‐pathologies investigated (i.e., CAA, TDP‐43, LBD, and AGD), which replicates some of the results from a recent study in a different cohort with a subsample of the biomarkers described here (Smirnov *et al*, 2022). Interestingly, we found that participants with AD and CAA pathologies had significantly higher levels of p‐tau217 than those with only CAA or only AD pathology. However, due to the low number of subjects with only AD pathology, we consider this as a hypothesis‐generating result that needs confirmation in a larger sample. Given the results from our study and previous studies, we emphasize the urgent need of developing new biomarkers capable of measuring the presence of these and other common co‐pathologies *in vivo* for a better diagnosis and prognosis for AD patients.

The main strength of this study was the availability of high‐performing assays of multiple plasma biomarkers, including the three main p‐tau biomarkers phosphorylated at different sites, in a relatively large neuropathological cohort. Thus, we were able to directly compare specific associations between all of these biomarkers to gold standard measures of pathology in the same participants. Further, the use of semi‐quantitative scores for measuring the burden of AD pathology, compared with the typical dichotomous scales used, allowed us to perform more complex analyses. However, some limitations must be acknowledged. First, we recognize the small number of participants with intermediate levels of pathology and those with or without certain co‐pathologies. Another limitation is the restricted number of participants in the longitudinal subsample, which may have reduced the power to find significant time interactions with plasma p‐tau181. In this analysis, the big difference in the number of blood draws and in their time lags may have also affected our results. Thus, our results in this regard should be taken with caution. Also, we could only analyze p‐tau217 and p‐tau181 in this longitudinal sample, which did not allow a complete comparison among biomarkers. Finally, we acknowledge that replication in an independent is needed to establish the robustness of our results.

In conclusion, our results support that plasma p‐tau217 and plasma p‐tau181 are specific markers of both amyloid plaques and tau tangles, whereas the Aβ42/40 ratio and p‐tau231 levels are markers strictly associated with plaques and GFAP with tangles. This is important when interpreting p‐tau measures in the A/T/N (Jack *et al*, 2016) context, as they may be more related to A (amyloid) than previously thought, as recently suggested (Groot *et al*, 2022; Moscoso *et al*, 2022; Therriault *et al*, 2022). Further, the combination of high‐performing assays of plasma p‐tau217 and the Aβ42/40 ratio gives the highest accuracy for predicting amyloid plaque load, while p‐tau217 alone may be sufficient to predict the load of tangles. These results may be useful to design prescreening strategies for clinical trials targeting amyloid and tau pathologies.

## Materials and Methods

### Participants

All samples were obtained through autopsies of subjects enrolled in the Arizona Study of Aging and Neurodegenerative Disorders and Brain and Body Donation Program (BBDP) at Banner Sun Health Research Institute (Beach *et al*, 2015). The BBDP recruits independently‐living normal and neurologically‐impaired elderly subjects predominantly from the surrounding Sun City's retirement communities. These volunteer research subjects are followed prospectively with annual standardized clinical assessments for the rest of their lives. Participants included in this study ranged from cognitively unimpaired to mild cognitive impairment and AD patients, as well as patients with other neurodegenerative diseases. We selected participants with both plasma and neuropathological exams available, including only those with all biomarkers available in the cross‐sectional analyses. Participants in the cross‐sectional analysis were also restricted as to those having blood drawing up to 5 years before death (mean (SD) [range] time: 482 (355) [9–1,760] days). All experiments were conducted in accordance with the Declaration of Helsinki. The operations of the Brain and Body Donation Program are approved by Institutional Review Boards and all participants or their legal representatives gave informed consent.

### Plasma biomarkers

Plasma p‐tau217 and p‐tau181 concentrations were measured in‐house using an immunoassay developed by Lilly Research Laboratories (IN, USA), each of which had performed very well in multiple studies and cohorts (Palmqvist *et al*, 2020; Janelidze *et al*, 2020a; Mielke *et al*, 2021; Thijssen *et al*, 2021). Plasma p‐tau231 concentration was also measured in‐house using a Simoa approach which was developed at the University of Gothenburg, which can detect Aβ pathology with high accuracy (Ashton *et al*, 2021). The remaining plasma biomarkers (Aβ42, Aβ40, GFAP, and NfL) were measured with prototype fully automated Elecsys® plasma immunoassays (updated versions for Aβ42 and Aβ40 (Palmqvist *et al*, 2022)) plasma immunoassays (not commercially available) cobas e 601 and cobas e 411 analyzers (Roche Diagnostics International Ltd, Rotkreuz, Switzerland), also in‐house (Palmqvist *et al*, 2019). Longitudinal samples were only analyzed for p‐tau217 and p‐tau181, and not the Elecsys measurements or p‐tau231 for logistic reasons.

Outliers were defined as subjects with values above or below more than 5 interquartile range of the third or the first quartile and were excluded from subsequent analyses. Only one plasma NfL value was considered an outlier. We also excluded another plasma NfL value that strongly affected the models (based on the standardized residuals) due to the very young age of the subject (44 years) and the strong correlation between NfL and age.

### Pathological measures

All neuropathological measures were performed by a single US‐certified neuropathologist (TGB).

#### Core AD pathology

Tissue processing methods have been detailed previously (Beach *et al*, 2008). Histopathological scoring was performed blinded to clinical and neuropathological diagnosis, as well as levels of the plasma biomarkers. Amyloid plaque and neurofibrillary tangle density were graded at standard sites in frontal, temporal, and parietal cortices, as well as the hippocampus and entorhinal cortex, as previously explained (Beach *et al*, 2008). To obtain the total plaque score, each region was first rated as none, sparse, moderate, or frequent, using the published CERAD templates (Mirra *et al*, 1991). These descriptive measures were then converted into 0–3 scores in each region that combined and gave the total plaque score with a maximum value of 15. Neurofibrillary tangle abundance was measured similarly using the CERAD templates. Additionally, Braak staging was performed based on the topographical distribution of neurofibrillary tangle change (Braak & Braak, 1991). Global CERAD scoring and Thal phases (Thal *et al*, 2002) were also assessed as a measure of amyloid pathology. Using these three global scales we obtained a global measure of ADNC as described in the NIA‐AA guidelines (Montine *et al*, 2012). Although the ADNC score was used as a four‐scale measure in some analyses, we also dichotomized as a significant AD pathology (nonsignificant AD pathology) ADNC when scores were intermediate or high (none or low).

#### Non‐AD pathology

CAA was graded on a 0–3 scale based analogously on CERAD templates (Mirra *et al*, 1991) and dichotomized as positive if the score was above 1. Immunohistochemical staining in 10 brain regions for p129 alpha‐synuclein, as well as Thioflavin‐S (for substantia nigra) was used as a secondary stain to detect Lewy bodies and were staged based on the Unified Staging System (Beach *et al*, 2009). TDP‐43 positivity, location of positivity, and morphology were recorded as explained previously (Arnold *et al*, 2013). Significant CWMR was defined as exceeding 25% of the total centrum semiovale area within one or more cerebral lobes using hematoxylin and eosin on large format section protocol (Dugger *et al*, 2014). AGD was defined as typical spindle‐shaped structures revealed by the Gallyas silver stain (Josephs *et al*, 2008; Sabbagh *et al*, 2009).

### Statistical analyses

First, partial Spearman's ρ was used to assess associations between each plasma biomarker (as dependent variable) and both plaques and tangles (as independent variables), independently. These models were adjusted for age, sex, and time between blood draw and death. To assess specific associations with each of these pathological measures, we also used partial Spearman's ρ further adjusting for the other pathology. Differences between two correlation coefficients were tested using a bootstrapping approach (*n* = 1,000). Specific contribution of plaque and tangle loads on each biomarker concentration was obtained as the percentage of partial Spearman's ρ of each pathology over the sum of the partial Spearman's ρ of the two pathologies. Diagnostic accuracies of plasma biomarkers were assessed using ROC curve analysis, with age, sex, and time between blood draw and death as covariates. When assessing the diagnostic accuracy of non‐AD pathologies, the ADNC status as a dichotomous variable was also included as a covariate. Differences in the area under the curve (AUC) between two ROC curves were compared with the DeLong test (Robin *et al*, 2011). Differences in plasma levels between pathological groups were assessed with Kruskal–Wallis tests, with the pairwise Wilcoxon test as a *post hoc* comparison among groups (only differences between consecutive groups tested). We used the R package MuMIn to select the most parsimonious models to predict both continuous and dichotomous pathological measures following procedures described earlier (Palmqvist *et al*, 2021). Only plasma biomarkers showing a significant association with each pathological measure in the initial univariable models were included as possible predictors and the aforementioned covariates. All covariates were included in the final parsimonious model (even when they were not significant) to allow a fair comparison against univariable models. When multiple p‐tau (at different phosphorylation sites) biomarkers were possible predictors, independent models for each p‐tau marker were performed and then compared based on the corrected Akaike criterion (AICc) to avoid multicollinearity problems. Plasma levels were log‐transformed for this analysis. Linear mixed models (LME) were used to assess longitudinal changes in plasma biomarkers. Three independent models were performed for each biomarker. In each one, the interaction between time and one measure of AD pathology (i. plaques, ii. tangles, or iii. presence of ADNC) was used as a predictor. The LME models were adjusted for age and sex and included random intercepts and fixed slopes due to the limited number of datapoints (median[range]: 2[2–5]). Statistical analyses were done using R version 4.1.0. Significance was set at *P* < 0.05 (two‐tailed) and corrected for multiple comparisons using a false discovery rate (FDR).

## Author contributions

**Gemma Salvadó:** Formal analysis; writing – original draft. **Rik Ossenkoppele:** Supervision; methodology; writing – review and editing. **Nicholas J Ashton:** Methodology; writing – review and editing. **Thomas G Beach:** Conceptualization; resources; funding acquisition; methodology; writing – review and editing. **Geidy E Serrano:** Resources; methodology; writing – review and editing. **Eric M Reiman:** Resources; funding acquisition. **Henrik Zetterberg:** Resources; methodology; writing – review and editing. **Niklas Mattsson‐Carlgren:** Supervision; writing – review and editing. **Shorena Janelidze:** Methodology; writing – review and editing. **Kaj Blennow:** Software; funding acquisition; methodology; writing – review and editing. **Oskar Hansson:** Conceptualization; resources; supervision; funding acquisition; project administration; writing – review and editing.

In addition to the CRediT author contributions listed above, the contributions in detail are:

GS, RO, and OH were involved in the study conception and design. GS analyzed the data. GS, RO, NM‐C, and OH interpreted the data. NJA and JS performed the experiments. GS wrote the manuscript. TGB, GES, ER, HZ, and KB participated in the acquisition of data. All authors critically reviewed and approved the final manuscript.

## Disclosure and competing interests statement

HZ has served at scientific advisory boards and/or as a consultant for Abbvie, Alector, ALZPath, Annexon, Apellis, Artery Therapeutics, AZTherapies, CogRx, Denali, Eisai, Nervgen, Novo Nordisk, Passage Bio, Pinteon Therapeutics, Red Abbey Labs, reMYND, Roche, Samumed, Siemens Healthineers, Triplet Therapeutics, and Wave, has given lectures in symposia sponsored by Cellectricon, Fujirebio, Alzecure, Biogen, and Roche, and is a co‐founder of Brain Biomarker Solutions in Gothenburg AB (BBS), which is a part of the GU Ventures Incubator Program (outside submitted work). KB has served as a consultant, at advisory boards, or at data monitoring committees for Abcam, Axon, BioArctic, Biogen, JOMDD/Shimadzu. Julius Clinical, Lilly, MagQu, Novartis, Ono Pharma, Pharmatrophix, Prothena, Roche Diagnostics, and Siemens Healthineers, and is a co‐founder of Brain Biomarker Solutions in Gothenburg AB (BBS), which is a part of the GU Ventures Incubator Program, outside the work presented in this paper. OH has acquired research support (for the institution) from ADx, AVID Radiopharmaceuticals, Biogen, Eli Lilly, Eisai, Fujirebio, GE Healthcare, Pfizer, and Roche. In the past 2 years, he has received consultancy/speaker fees from AC Immune, Amylyx, Alzpath, BioArctic, Biogen, Cerveau, Fujirebio, Genentech, Novartis, Roche, and Siemens.

## Supporting information



Appendix

## Data Availability

Anonymized data will be shared by request from a qualified academic investigator and as long as data transfer is in agreement with USA legislation on the general data protection regulation and decisions by the Institutional Review Boards of the Brain and Body Donation Program and the Arizona Study of Aging and Neurodegenerative Disorders.
