# Supplementary material for: Specific associations between plasma biomarkers and postmortem amyloid plaque and tau tangle loads
Source: EMBO Mol Med. 2023 Mar 13;15(5):e17123. doi: 10.15252/emmm.202217123 (PMC10165361; doi:10.15252/emmm.202217123)
Supplement: Supplementary file 1 — Appendix [file EMMM-15-e17123-s001.pdf]

Specific associations between plasma biomarkers and post-mortem amyloid plaque and tau tangle loads

Gemma Salvadó, Rik Ossenkoppele, Nicholas J. Ashton, Thomas G. Beach, Geidy E. Serrano, Eric M. Reiman, Henrik Zetterberg, Niklas Mattsson-Carlgren, Shorena Janelidze, Kaj Blennow, Oskar Hansson

Table of content

Appendix Tables:

Appendix Table S1 Associations between plasma biomarkers and amyloid plaque or neurofibrillary tau tangle loads in ADNC groups

Appendix Table S2 Contribution of amyloid plaque load and tau tangle load on plasma levels

Appendix Table S3 Plasma difference by ADNC levels

Appendix Table S4 Plasma biomarkers for predicting ADNC classification

Appendix Table S5 Plasma biomarkers for predicting CERAD classification

Appendix Table S6 Plasma biomarkers for predicting Braak staging classification

Appendix Table S7 Parsimonious models to predict CERAD and Braak staging classification

Appendix Table S8 Plasma biomarkers for predicting presence of cerebral white matter rarefaction

Appendix Table S9 Plasma biomarkers for predicting presence of CAA

Appendix Table S10 Plasma biomarkers for predicting presence of LBD

Appendix Table S11 Plasma biomarkers for predicting presence of TDP-43 pathology

Appendix Table S12 Plasma biomarkers for predicting presence of AGD

Appendix Table S13 Parsimonious models to predict AD-related pathology using the p-tau217/Aβ42 ratio

Appendix Table S14 Demographic characteristics of the longitudinal subsample

Appendix Figures:

Appendix Figure S1 Association between plaques and tangles

Appendix Figure S2 Plasma levels by CERAD classification

Appendix Figure S3 Plasma levels by Braak staging classification

Appendix Figure S4 Plasma biomarkers for predicting presence of cerebral white matter rarefaction

Appendix Figure S5 ROC curves showing diagnostic accuracy of plasma biomarkers for predicting presence of co-pathologies

Appendix Figure S6 Plasma levels by presence or absence of CAA

Appendix Figure S7 Plasma levels by presence or absence of LBD

Appendix Figure S8 Plasma levels by presence or absence of TDP-43 pathology

Appendix Figure S9 Plasma levels by presence or absence of CWMR

Appendix Figure S10 Plasma levels by presence or absence of AGD
Appendix Figure S11 Plasma levels by presence of AD pathology and/or CAA
Appendix Figure S12 Plasma levels by presence of AD pathology and/or LBD
Appendix Figure S13 Plasma levels by presence of AD pathology and/or AGD
Appendix Figure S14 Plasma levels by presence of AD pathology and/or CWMR
Appendix Figure S15 Plasma levels by presence of AD pathology and/or TDP-43
Appendix Figure S16 Plasma levels by presence of AD pathology and/or primary tauopathies
Appendix Figure S17 Associations between longitudinal changes of plasma biomarkers and presence of ADNC at death

Appendix Tables

	r [95%CI] plaques	p plaques	r [95%CI] tangles	p tangles
**ADNC none/low**				
**p-tau217**	0.10 [-0.28, 0.43]	1.000	0.11 [-0.25, 0.39]	1.000
**p-tau181**	0.22 [-0.04, 0.46]	1.000	0.00 [-0.32, 0.25]	0.442
**p-tau231**	0.08 [-0.22, 0.45]	1.000	-0.06 [-0.37, 0.20]	0.288
**Aβ42/40**	-0.33 [-0.57, -0.07]	<0.001	0.08 [-0.25, 0.42]	1.000
**GFAP**	-0.02 [-0.41, 0.54]	0.418	0.08 [-0.28, 0.44]	1.000
**NfL**	0.03 [-0.29, 0.51]	1.000	0.06 [-0.35, 0.37]	1.000
**ADNC intermediate/high**				
**p-tau217**	0.41 [0.15, 0.62]	0.049	0.56 [0.30, 0.75]	0.001
**p-tau181**	0.23 [-0.10, 0.50]	0.588	0.35 [0.04, 0.59]	0.122
**p-tau231**	0.35 [0.08, 0.57]	0.122	0.49 [0.22, 0.69]	0.008
**Aβ42/40**	-0.30 [-0.56, -0.04]	<0.001	-0.01 [-0.33, 0.29]	0.291
**GFAP**	0.03 [-0.26, 0.29]	1.000	0.47 [0.17, 0.67]	0.011
**NfL**	0.04 [-0.22, 0.36]	1.000	0.20 [-0.08, 0.45]	0.778

Appendix Table S1 Associations between plasma biomarkers and amyloid plaque or neurofibrillary tau tangle loads in ADNC groups

Partial Spearman's r was used for these analyses with each biomarker as an outcome, in independent models, and amyloid load or tau load as independent adjusted for age, sex, and time between blood sampling and death. ADNC none/low group represented participants without significant AD pathology, and ADNC intermediate/high group referred to participants with significant AD pathology. P-values were FDR-corrected for multiple comparisons. Abbreviations: Aβ, amyloid-β; FDR, false-discovery rate; GFAP, glial fibrillary acidic protein; NfL, neurofilament light; p-tau, phosphorylated tau.

	Partial r plaques	% partial r plaques	Partial r tangles	% partial r tangles
**p-tau217**	0.24	40.4	0.18	30.70
**p-tau181**	0.15	35.7	0.07	17.10
**p-tau231**	0.06	45.9	0.00	0.00
**Aβ42/40**	0.24	77.6	0.00	0.00
**GFAP**	0.03	6.4	0.13	30.40
**NfL**	-0.01	0.0	0.01	4.30

Appendix Table S2 Contribution of amyloid plaque load and tau tangle load on plasma levels

Partial Spearman's r was used for these analyses with each biomarker as a dependent variable, and amyloid plaque load or tau tangle load as independent variable. When assessing associations with plaques, we also adjusted for tau load, and the reverse when associations with tangles. We adjusted for age, sex, and time between blood sampling and death in all cases. Percentual partial Spearman's r is calculated as the ratio of partial r over the sum of the two pathologies' partial Spearman's r (%partial r = 100*partial r /(partial r_{plaque} + partial r_{tangle}).

Abbreviations: Aβ, amyloid-β; FDR, false discovery rate; GFAP, glial fibrillary acidic protein; NfL, neurofilament light; p-tau, phosphorylated tau.

ADNC	Fold increase none-low	p-value none-low	Fold increase low-interm	p-value low-interm	Fold increase interm-high	p-value interm-high
**p-tau217**	0.39	**0.038**	0.61	**0.002**	1.46	**<0.001**
**p-tau181**	0.30	0.138	0.15	**0.138**	0.88	**<0.001**
**p-tau231**	0.32	0.379	-0.01	0.703	0.37	**0.005**
**Aβ42/40**	-0.05	0.114	-0.06	**0.016**	-0.04	0.060
**GFAP**	0.10	0.621	0.29	0.102	0.21	0.140
**NfL**	0.18	0.735	-0.04	0.976	0.03	0.976

Appendix Table S3 Plasma difference by ADNC levels

Kruskal-Wallis tests were used to investigate differences in all plasma biomarkers by ADNC status. *Post hoc* analyses were performed with pairwise Wilcoxon rank sum tests among consecutive levels. Fold increases between consecutive levels were also calculated using the lowest level of each comparison as a reference. Significant differences (p<0.05 FDR-corrected) are shown in bold.

Abbreviations: Aβ amyloid-β; ADNC, Alzheimer's disease neuropathologic change; FDR, false discovery rate; GFAP, glial fibrillary acidic protein; NfL, neurofilament light; p-tau, phosphorylated tau.

ADNC	β [95%CI]	p-value β	AUC[95%CI]	R^2	AICc	p-value DeLong
**Basic**	-	-	0.61 [0.50, 0.72]	0.07	146.60	<0.001
**p-tau217**	2.22 [1.43, 3.22]	**<0.001**	0.88 [0.81, 0.95]	0.63	99.37	Ref
**p-tau181**	1.43 [0.84, 2.13]	**<0.001**	0.81 [0.73, 0.9]	0.42	119.96	0.002
**p-tau231**	0.69 [0.24, 1.19]	**0.004**	0.72 [0.63, 0.82]	0.19	139.30	0.002
**Aβ42/40**	-1.24 [-1.86, -0.71]	**<0.001**	0.80 [0.72, 0.89]	0.35	123.71	0.123
**GFAP**	1.10 [0.56, 1.74]	**<0.001**	0.77 [0.68, 0.86]	0.29	130.78	0.015
**NfL**	0.09 [-0.36, 0.55]	0.698	0.61 [0.50, 0.71]	0.07	148.66	<0.001

Appendix Table S4 Plasma biomarkers for predicting ADNC classification

Generalized linear regression models were used to investigate these associations in independent models including: age, sex, and time between blood sampling and death as covariates. ADNC was used as dependent variable, dichotomized as negative (none/low) or positive (intermediate/high). The basic model includes only covariates. Significant associations ($p<0.05$) between plasma biomarkers and ADNC positivity are shown in bold. Differences between the AUCs were calculated using the DeLong test, with the highest AUC as reference (ref.), shown in the last column. Significant differences ($p<0.05$) can be understood as significantly weaker predictive power compared with that of p-tau217.

Abbreviations: Aβ, amyloid-β; AICc, corrected Akaike criterion, AUC, area under the curve; CERAD, Consortium to establish a registry for Alzheimer's disease; CI, confidence interval; GFAP, glial fibrillary acidic protein; NfL, neurofilament light; p-tau, phosphorylated tau.

CERAD	β [95%CI]	p-value β	AUC[95%CI]	R^2	AICc	p-value DeLong
**Basic**	-	-	0.62 [0.51, 0.73]	0.08	145.98	<0.001
**p-tau217**	2.35 [1.53, 3.42]	**<0.001**	0.89 [0.83, 0.96]	0.66	96.12	ref.
**p-tau181**	1.53 [0.92, 2.27]	**<0.001**	0.83 [0.74, 0.91]	0.45	116.69	0.003
**p-tau231**	0.80 [0.34, 1.33]	**0.001**	0.74 [0.64, 0.83]	0.23	135.99	0.001
**Aβ42/40**	-1.37 [-2.04, -0.81]	**<0.001**	0.82 [0.74, 0.90]	0.40	119.36	0.104
**GFAP**	1.44 [0.82, 2.20]	**<0.001**	0.81 [0.72, 0.89]	0.40	122.14	0.039
**NfL**	0.26 [-0.19, 0.73]	0.263	0.62 [0.51, 0.73]	0.09	146.91	<0.001

Appendix Table S5 Plasma biomarkers for predicting CERAD classification

Generalized linear regression models were used to investigate these associations in independent models including: age, sex, and time between blood sampling and death as covariates. CERAD classification was used as dependent variable, dichotomized as negative (zero/sparse) or positive (moderate/frequent). The basic model includes only covariates. Significant associations ($p<0.05$) between plasma biomarkers and CERAD positivity are shown in bold. Differences between the AUCs were calculated using the DeLong test, with the highest AUC as reference (ref.), shown in the last column. Significant differences ($p<0.05$) can be understood as significantly weaker predictive power compared with that of p-tau217.

Abbreviations: Aβ, amyloid-β; AICc, corrected Akaike criterion; AUC, area under the curve; CERAD, Consortium to establish a registry for Alzheimer's disease; CI, confidence interval; GFAP, glial fibrillary acidic protein; NfL, neurofilament light; p-tau, phosphorylated tau.

Braak staging	β [95%CI]	p-value β	AUC[95%CI]	R^2	AICc	p-value DeLong
**Basic**	-	-	0.73 [0.63, 0.83]	0.23	123.95	<0.001
**p-tau217**	2.44 [1.57, 3.57]	**<0.001**	0.93 [0.87, 0.98]	0.73	77.04	ref.
**p-tau181**	1.46 [0.87, 2.17]	**<0.001**	0.87 [0.79, 0.94]	0.54	97.00	0.004
**p-tau231**	0.65 [0.16, 1.20]	**0.013**	0.78 [0.69, 0.87]	0.33	119.31	<0.001
**Aβ42/40**	-0.67 [-1.2, -0.20]	**0.008**	0.77 [0.68, 0.87]	0.32	118.31	0.001
**GFAP**	1.48 [0.83, 2.28]	**<0.001**	0.86 [0.79, 0.94]	0.51	102.07	0.061
**NfL**	0.48 [-0.03, 1.02]	0.070	0.75 [0.66, 0.85]	0.28	122.80	<0.001

Appendix Table S6 Plasma biomarkers for predicting Braak staging classification
Generalized linear regression models were used to investigate these associations in independent models including: age, sex and time between blood sampling and death as covariates. Braak staging was used as dependent variable, dichotomized as negative (0-IV) or positive (V-VI). The basic model includes only covariates. Significant associations ($p<0.05$) between plasma biomarkers and Braak staging positivity are shown in bold. Differences between the AUCs were calculated using the DeLong test, with the highest AUC as reference (ref.), shown in the last column. Significant differences ($p<0.05$) can be understood as significantly weaker predictive power compared with that of p-tau217.
Abbreviations: Aβ, amyloid-β; AICc, corrected Akaike criterion, AUC, area under the curve; CI, confidence interval; GFAP, glial fibrillary acidic protein; NfL, neurofilament light; p-tau, phosphorylated tau.

	β [95%CI]	p-value association	R^2	AICc	AUC[95%CI]
**CERAD**					
p-tau217	1.94 [1.16, 2.93]	**<0.001**	0.70	87.89	0.91 [0.86, 0.97]
Aβ42/40	-1.22 [-2.08, -0.46]	**0.003**			
Age	0.26 [-0.32, 0.88]	0.386			
Sex	-0.20 [-1.42, 1.02]	0.750			
Time blood-death	0.25 [-0.47, 1.03]	0.499			
**Braak stages**					
p-tau217	2.44 [1.57, 3.57]	**<0.001**	0.73	77.04	0.93 [0.87, 0.98]
Age	-0.31 [-0.98, 0.33]	0.347			
Sex	-1.78 [-3.34, -0.45]	**0.014**			
Time blood-death	0.83 [0.10, 1.67]	**0.035**			

Appendix Table S7 Parsimonious models to predict CERAD and Braak staging classification

Parsimonious models were selected as those that better explained each AD-related scale with the smaller number of predictors based on the AICc criterion. Initial models included basic covariates (age, sex, and time between blood sampling) and all biomarkers that showed a significant association in the univariate analyses. Men are the reference sex group. CERAD classification was used as dependent variable, dichotomized as negative (zero/sparse) or positive (moderate/frequent). Braak staging was used as dependent variable, dichotomized as negative (0-IV) or positive (V-VI).

Abbreviations: Aβ, amyloid-β; AICc, corrected Akaike criterion, AUC, area under the curve; CERAD, Consortium to Establish a Registry for Alzheimer's Disease; CI, confidence intervals; p-tau, phosphorylated tau.

CWMR	β [95%CI]	p-value β	AUC[95%CI]	R^2	AICc	p-value DeLong
Basic	-	-	0.65 [0.54, 0.76]	0.10	146.18	Ref.
p-tau217	0.58 [0.04, 1.18]	0.043	0.70 [0.60, 0.80]	0.16	144.02	0.145
p-tau181	0.28 [-0.21, 0.78]	0.271	0.67 [0.57, 0.78]	0.12	147.20	0.359
p-tau231	0.01 [-0.42, 0.45]	0.957	0.65 [0.55, 0.76]	0.10	148.43	0.548
Aβ42/40	0.07 [-0.40, 0.55]	0.774	0.66 [0.55, 0.76]	0.10	148.35	0.527
GFAP	0.40 [-0.08, 0.92]	0.111	0.68 [0.58, 0.79]	0.13	145.80	0.303
NfL	0.88 [0.36, 1.47]	0.002	0.76 [0.66, 0.85]	0.25	136.57	**0.028**

Appendix Table S8 Plasma biomarkers for predicting presence of cerebral white matter rarefaction

Generalized linear regression models were used to investigate these associations in independent models including: age, sex, time between blood sampling and death, and ADNC status, as a dichotomous variable, as covariates. CWMR was used as dependent variable, dichotomized as negative or positive. ADNC was dichotomized as negative (none/low) or positive (intermediate/high). The basic model includes only covariates. Differences between the AUCs were calculated using the DeLong test, with the AUC from the basic model as reference (ref.), shown in the last column. Significant differences ($p<0.05$) can be understood as significantly greater predictive power compared to that of only using covariates and are shown in bold.

Abbreviations: Aβ, amyloid-β; AICc, corrected Akaike criterion, AUC, area under the curve; CI, confidence interval; CWMR, cerebral white matter rarefaction; GFAP, glial fibrillary acidic protein; NfL, neurofilament light; p-tau, phosphorylated tau.

CAA	β [95%CI]	p-value β	AUC[95%CI]	R^2	AICc	p-value DeLong
Basic	-	-	0.82 [0.73, 0.91]	0.36	113.41	ref.
p-tau217	0.61 [-0.05, 1.32]	0.078	0.85 [0.77, 0.93]	0.40	112.38	0.209
p-tau181	0.49 [-0.13, 1.15]	0.129	0.84 [0.76, 0.92]	0.39	113.25	0.297
p-tau231	-0.01 [-0.52, 0.49]	0.970	0.82 [0.73, 0.91]	0.36	115.66	0.660
Aβ42/40	-0.66 [-1.30, -0.08]	0.032	0.84 [0.76, 0.92]	0.42	110.60	0.225
GFAP	0.09 [-0.51, 0.69]	0.764	0.82 [0.73, 0.91]	0.36	115.57	0.860
NfL	-0.18 [-0.76, 0.38]	0.527	0.81 [0.72, 0.90]	0.37	115.26	0.587

Appendix Table S9 Plasma biomarkers for predicting presence of CAA

Generalized linear regression models were used to investigate these associations in independent models including: age, sex, time between blood sampling and death, amyloid plaque and tau tangle measures as covariates. Presence or absence of CAA was used as dependent variable, dichotomized as negative (0-1) or positive (2-3). The basic model includes only covariates. Differences between the AUCs were calculated using the DeLong test, with the AUC from the basic model as reference (ref.), shown in the last column. Significant differences ($p<0.05$) can be understood as significantly greater predictive power compared with that of only using covariates. Abbreviations: Aβ, amyloid-β; AICc, corrected Akaike criterion, CAA, cerebral amyloid angiopathy; AUC, area under the curve; CI, confidence interval; GFAP, glial fibrillary acidic protein; NfL, neurofilament light; p-tau, phosphorylated tau.

LBD	β [95%CI]	p-value β	AUC[95%CI]	R^2	AICc	p-value DeLong
Basic	-	-	0.65 [0.51, 0.8]	0.08	111.53	ref.
p-tau217	0.12 [-0.52, 0.81]	0.727	0.64 [0.50, 0.79]	0.08	113.65	0.228
p-tau181	0.07 [-0.54, 0.66]	0.829	0.65 [0.50, 0.79]	0.08	113.73	0.495
p-tau231	0.04 [-0.49, 0.56]	0.890	0.65 [0.50, 0.79]	0.08	113.76	0.246
Aβ42/40	0.67 [0.07, 1.33]	0.036	0.70 [0.58, 0.83]	0.15	108.98	0.364
GFAP	0.02 [-0.57, 0.61]	0.954	0.65 [0.51, 0.80]	0.08	113.77	0.847
NfL	0.01 [-0.55, 0.56]	0.965	0.65 [0.51, 0.80]	0.08	113.78	0.339

Appendix Table S10 Plasma biomarkers for predicting presence of LBD

Generalized linear regression models were used to investigate these associations in independent models including: age, sex, time between blood sampling and death, amyloid plaque and tau tangle measures as covariates. Presence or absence of LBD was used as dependent variable, dichotomized as negative or positive. The basic model includes only covariates. Differences between the AUCs were calculated using the DeLong test, with the AUC from the basic model as reference (ref.), shown in the last column. Significant differences ($p<0.05$) can be understood as significantly greater predictive power compared with that of only using covariates.

Abbreviations: Aβ, amyloid-β; AICc, corrected Akaike criterion, AUC, area under the curve; CI, confidence interval; GFAP, glial fibrillary acidic protein; LBD, Lewy body disease; NfL, neurofilament light; p-tau, phosphorylated tau.

TDP-43	β [95%CI]	p-value β	AUC[95%CI]	R^2	AICc	p-value DeLong
Basic	-	-	0.60 [0.43, 0.77]	0.05	82.35	-
p-tau217	0.59 [-0.14, 1.42]	0.134	0.70 [0.56, 0.84]	0.11	82.40	0.106
p-tau181	0.35 [-0.30, 1.05]	0.296	0.65 [0.50, 0.81]	0.08	83.72	0.215
p-tau231	-0.01 [-0.57, 0.55]	0.977	0.60 [0.44, 0.77]	0.05	84.84	0.528
Aβ42/40	-0.32 [-1.00, 0.32]	0.337	0.62 [0.46, 0.79]	0.08	83.90	0.564
GFAP	0.03 [-0.62, 0.68]	0.936	0.60 [0.43, 0.77]	0.05	84.84	0.892
NfL	0.20 [-0.38, 0.78]	0.498	0.63 [0.47, 0.78]	0.06	84.39	0.498

Appendix Table S11 Plasma biomarkers for predicting presence of TDP-43 pathology

Generalized linear regression models were used to investigate these associations in independent models including: age, sex, time between blood sampling and death, amyloid plaque and tau tangle measures as covariates. Presence or absence of TDP-43 pathology was used as dependent variable, dichotomized as negative or positive. Differences between the AUCs were calculated using the DeLong test, with the AUC from the basic model as reference (ref.), shown in the last column. Significant differences ($p<0.05$) can be understood as significantly greater predictive power compared with that of only using covariates.

Abbreviations: Aβ, amyloid-β; AICc, corrected Akaike criterion, AUC, area under the curve; CI, confidence interval; GFAP, glial fibrillary acidic protein; NfL, neurofilament light; p-tau, phosphorylated tau; TDP-43, TAR DNA binding protein-43.

AGD	β [95%CI]	p-value β	AUC[95%CI]	R^2	AICc	p-value DeLong
Basic	-	-	0.53 [0.41, 0.66]	0.01	131.31	ref.
p-tau217	-0.60 [-1.21, -0.04]	0.042	0.65 [0.54, 0.77]	0.07	129.22	0.073
p-tau181	-0.35 [-0.92, 0.18]	0.204	0.60 [0.48, 0.72]	0.03	131.89	0.257
p-tau231	-0.27 [-0.76, 0.20]	0.274	0.58 [0.47, 0.70]	0.03	132.35	0.370
Aβ42/40	0.53 [0.00, 1.10]	0.058	0.63 [0.52, 0.75]	0.06	129.73	0.180
GFAP	-0.11 [-0.66, 0.42]	0.684	0.54 [0.41, 0.67]	0.01	133.40	0.880
NfL	-0.22 [-0.76, 0.28]	0.406	0.55 [0.42, 0.67]	0.02	132.86	0.775

Appendix Table S12 Plasma biomarkers for predicting presence of AGD

Generalized linear regression models were used to investigate these associations in independent models including: age, sex, time between blood sampling and death, amyloid plaque and tau tangle measures as covariates. Presence or absence of AGD pathology was used as dependent variable, dichotomized as negative or positive. The basic model includes only covariates. Differences between the AUCs were calculated using the DeLong test, with the AUC from the basic model as reference (ref.), shown in the last column. Significant differences ($p<0.05$) can be understood as significantly greater predictive power compared with that of only using covariates. Abbreviations: Aβ, amyloid-β; AGD, argyrophilic grain disease; AICc, corrected Akaike criterion, AUC, area under the curve; CI, confidence interval; GFAP, glial fibrillary acidic protein; NfL, neurofilament light; p-tau, phosphorylated tau.

	β [95%CI]	p-value association	R^2	AICc	AUC[95%CI]
**Plaques**					
p-tau217/Aβ42 ratio	0.63 [0.49, 0.78]	**<0.001**	0.60	210.1	NA
Aβ42/40	-0.26 [-0.4, -0.12]	**<0.001**			
Age	0.08 [-0.05, 0.21]	0.202			
Sex	0.01 [-0.25, 0.27]	0.929			
Time blood-death	-0.02 [-0.15, 0.11]	0.804			
**Tangles**					
p-tau217/Aβ42 ratio	0.66 [0.52, 0.80]	**<0.001**	0.52	228.7	NA
Age	0.08 [-0.06, 0.22]	0.281			
Sex	-0.33 [-0.61, -0.05]	**0.023**			
Time blood-death	0.11 [-0.03, 0.26]	0.113			
**ADNC**					
p-tau217/Aβ42 ratio	2.65 [1.75, 3.82]	**<0.001**	0.70	88.71	0.91 [0.85, 0.97]
Age	0.56 [0, 1.19]	0.061			
Sex	-0.29 [-1.47, 0.85]	0.618			
Time blood-death	0.32 [-0.43, 1.1]	0.413			

Appendix Table S13 Parsimonious models to predict AD-related pathology using the p-tau217/Aβ42 ratio

Parsimonious models were selected as those that better explained each AD-pathology measure with the smaller number of predictors based on the AICc criterion. Initial models included basic covariates (age, sex, and time between blood sampling) and all biomarkers that showed a significant association in the univariate analyses including both p-tau217 alone and p-tau217/Aβ42 ratio. Men are the reference sex group.

Abbreviations: Aβ, amyloid-β; AICc, corrected Akaike criterion, ADNC, Alzheimer's disease neuropathologic change; AUC, area under the curve; CI, confidence interval; p-tau, phosphorylated tau.

	Overall (n=48)	ADNC – negative (n=22)	ADNC – positive (n=26)
**Age at baseline, mean(SD)**	85.6 (7.99)	84.9 (6.92)	86.2 (8.88)
**Women, n(%)**	21 (43.8%)	10 (45.5%)	11 (42.3%)
**APOE-e4 carrier, n (%)**	12 (25.0%)	1 (4.5%)	11 (42.3%)
**Plaque total, mean(SD)**	6.47 (5.98)	0.818 (1.22)	11.3 (3.76)
**CERAD moderate/frequent, n(%)**	26 (54.2%)	0 (0%)	26 (100%)
**Tangle total, mean(SD)**	6.94 (2.69)	5.86 (1.68)	7.85 (3.07)
**Braak stage, n(%)**			
**I-II**	1 (2.1%)	1 (4.5%)	0 (0%)
**III-IV**	40 (83.3%)	20 (90.9%)	20 (76.9%)
**V-VI**	7 (14.6%)	1 (4.5%)	6 (23.1%)
**Timepoints, median[range]**	2 [2-5]	2 [2-5]	2 [2-4]
**Time difference, days, mean(SD)**	1,378 (1,357)	1,411 (1,398)	1,350 (1,349)

Appendix Table S14 Demographic characteristics of the longitudinal subsample

ADNC was dichotomized as: negative (none/low) and positive (intermediate/high).

Abbreviations: ADNC, Alzheimer's disease neuropathologic change; CERAD, Consortium to Establish a Registry for Alzheimer's Disease.

Appendix Figures

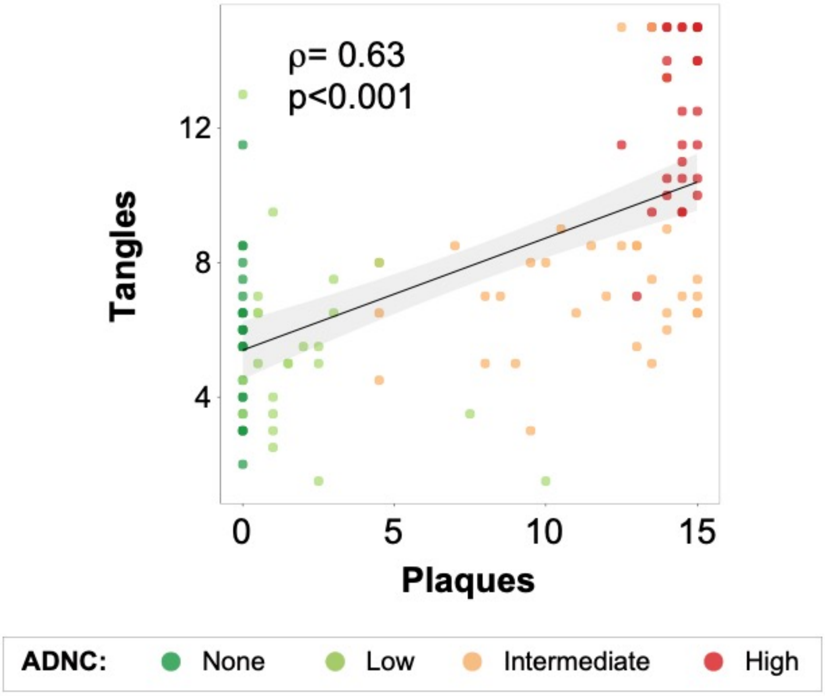

Appendix Figure S1 Association between plaques and tangles

Partial Spearman's r was used to obtain the correlation between the two measures (shown in the plot), adjusting for age, sex and time between blood sampling and death. Plaque and tangle loads were measured in a semi-quantitative scale from 0 to 3 using the CERAD (Mirra *et al*, 1991) templates in five different regions that were added up to a total score ranging from 0 to 15. Datapoints are coloured based on the ADNC classification, which refers to a measure of global AD pathology.

Abbreviations: ADNC, Alzheimer's disease neuropathologic change; CERAD, Consortium to Establish a Registry for Alzheimer's Disease.

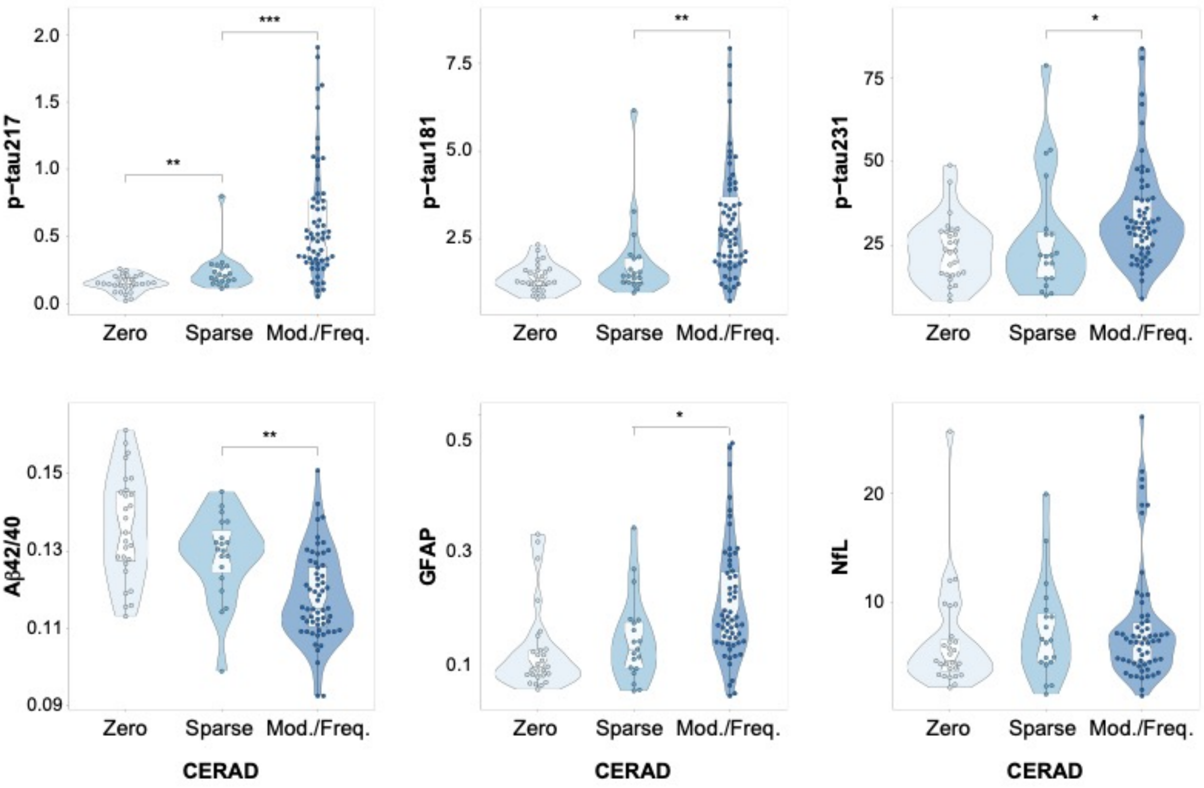

Appendix Figure S2 Plasma levels by CERAD classification

The CERAD (Mirra *et al*, 1991) scale refers to a measure of amyloid pathology. Groups were compared using a Kruskal-Wallis test.

*** $p<0.001$; ** $p<0.010$; * $p<0.050$

Abbreviations: Aβ, amyloid-β; CERAD, Consortium to Establish a Registry for Alzheimer's Disease; GFAP, glial fibrillary acidic protein; NfL, neurofilament light; p-tau, phosphorylated tau.

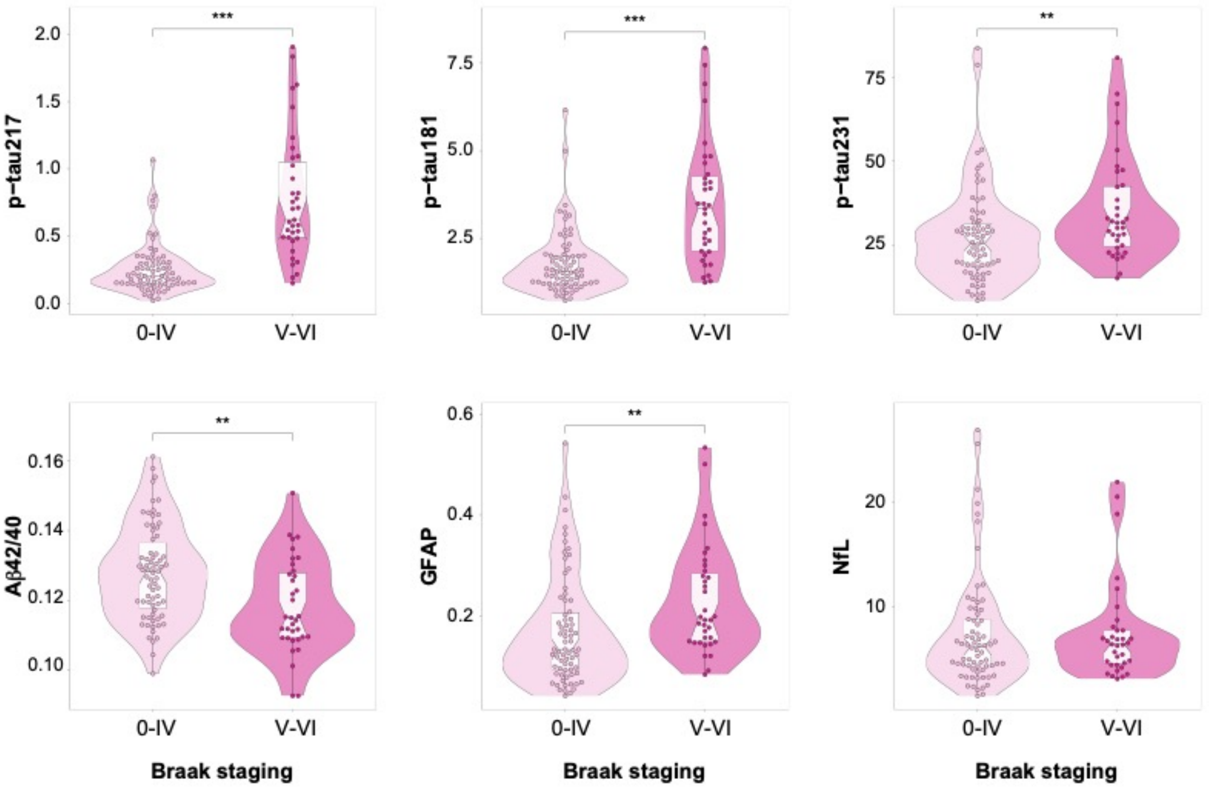

Appendix Figure S3 Plasma levels by Braak staging classification

The Braak staging (Braak & Braak, 1991) system refers to a measure of tangle pathology. Groups were compared using a Kruskal-Wallis test.

*** $p<0.001$; ** $p<0.010$; * $p<0.050$

Abbreviations: Aβ, amyloid-β; GFAP, glial fibrillary acidic protein; NfL, neurofilament light; p-tau, phosphorylated tau.

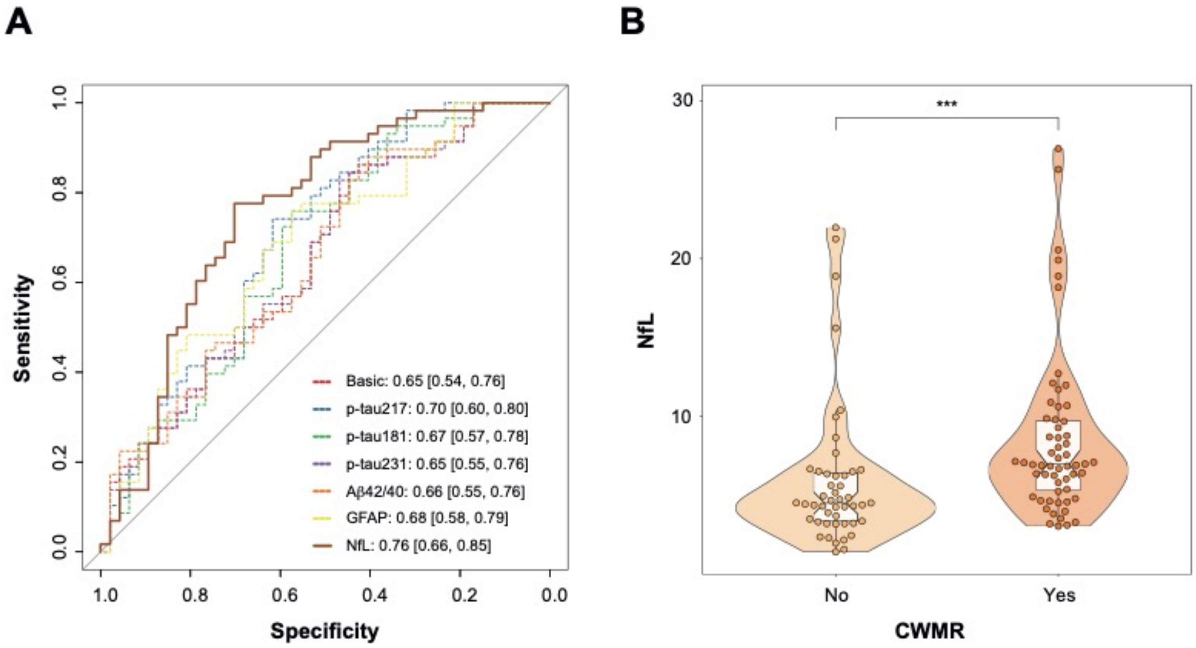

Appendix Figure S4 Plasma biomarkers for predicting presence of cerebral white matter rarefaction

ROC curves for all individual plasma biomarkers are shown in the left panel (a). In the ROC curves, all models included: age, sex, time between blood sampling and death, and presence of ADNC as a dichotomous variable as covariates. CWMR was used as dependent variable, dichotomized as negative or positive. ADNC was dichotomized as negative (none/low) or positive (intermediate/high). The basic model includes only covariates. AUCs and 95%CI are shown in the figure. The individual biomarker with best performance is shown as a solid bold line. Dashed lines represent individual biomarkers with significantly ($p<0.05$) lower AUC than the best individual biomarker. Only the addition of plasma NfL showed a significantly higher AUC than that of the basic model. Boxplot of plasma NfL levels by presence/absence of CWMR is shown in the right panel (b).

Abbreviations: Aβ, amyloid-β; ADNC, Alzheimer's disease neuropathologic change; AUC, area under the curve; CI, confidence interval; CWMR, cerebral white matter rarefaction; GFAP, glial fibrillary acidic protein; NfL, neurofilament light; p-tau, phosphorylated tau; ROC, receiver operating characteristic.

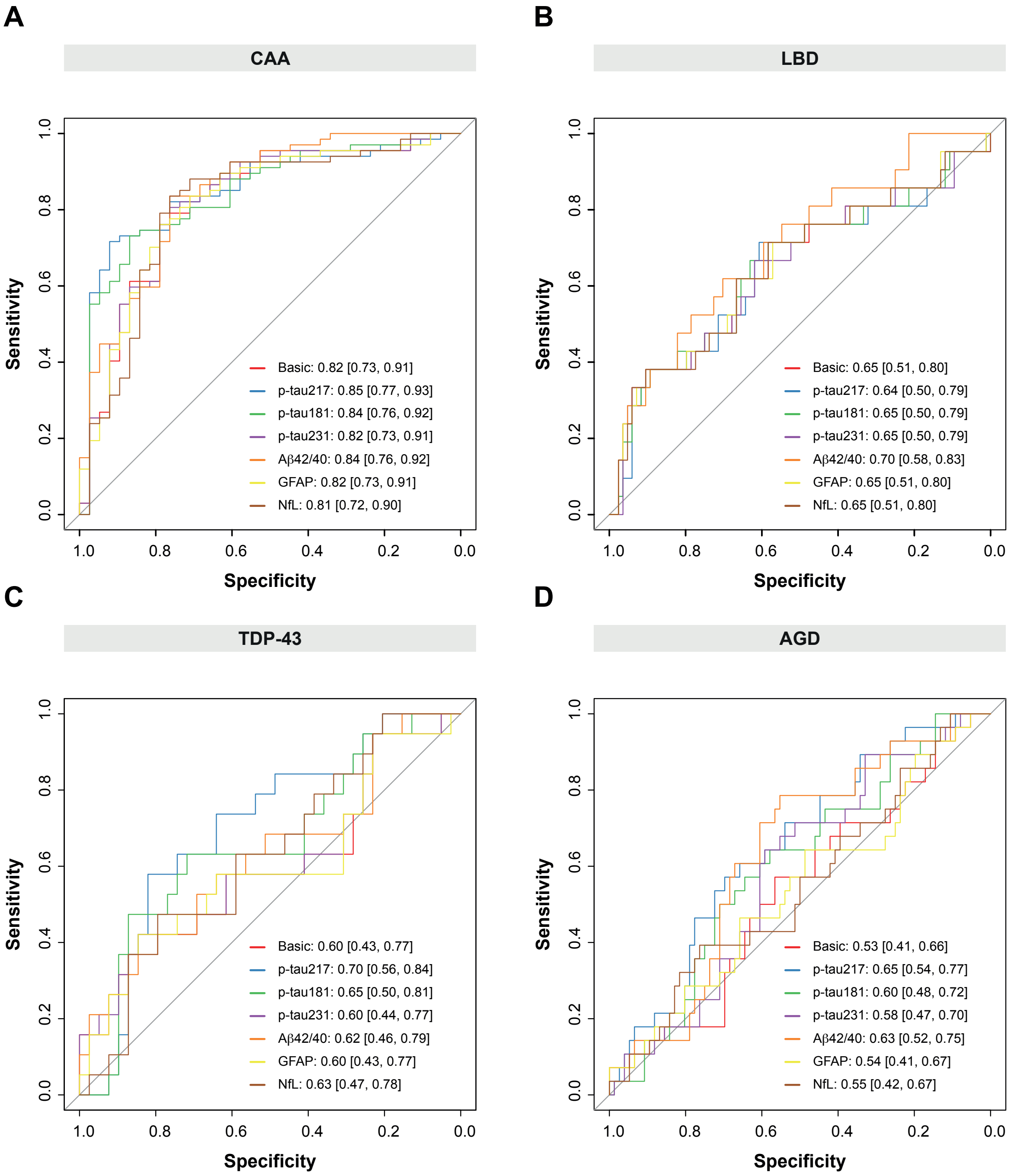

Appendix Figure S5 ROC curves showing diagnostic accuracy of plasma biomarkers for predicting presence of co-pathologies

ROC curves for predicting: CAA (a), LBD (b), TDP-43 (c) and AGD (d). All models included: age, sex, time between blood sampling, and death and presence of ADNC as a dichotomous variable as covariates. ADNC was dichotomized as negative (none/low) or positive (intermediate/high). The basic model includes only covariates. AUCs and 95%CI are shown in the figure.

Abbreviations: Aβ, amyloid-β; AUC, area under the curve, CAA, cerebral amyloid angiopathy; CI, confidence interval; GFAP, glial fibrillary acidic protein; LBD, Lewy body disease; NfL,

neurofilament light; p-tau, phosphorylated tau; ROC, receiver operating characteristic; TDP-43, TAR DNA binding protein 43.

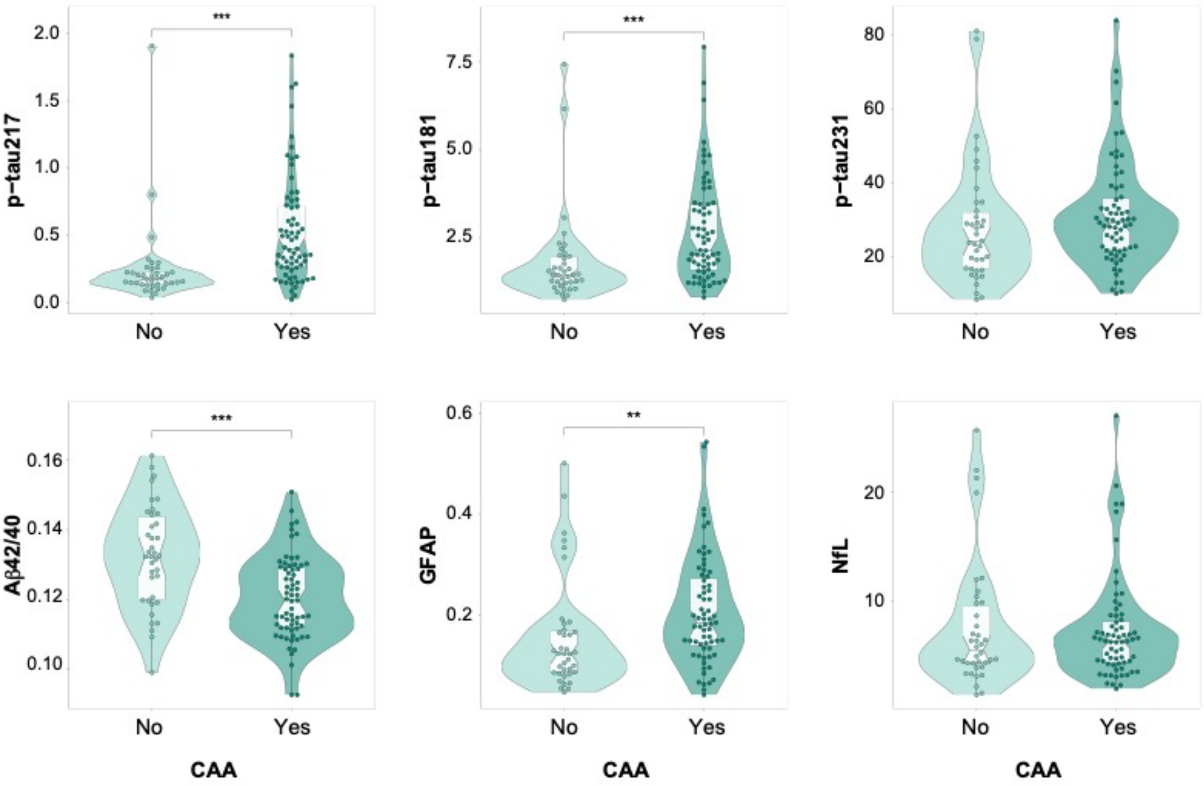

Appendix Figure S6 Plasma levels by presence or absence of CAA

Groups were compared using a Kruskal-Wallis test. None of these differences remained significant after adjusting for covariates (Appendix Table S8).

*** p<0.001; ** p<0.010 ; * p<0.050

Abbreviations: Aβ, amyloid-β; CAA, cerebral amyloid angiopathy; GFAP, glial fibrillary acidic protein; NfL, neurofilament light; p-tau, phosphorylated tau.

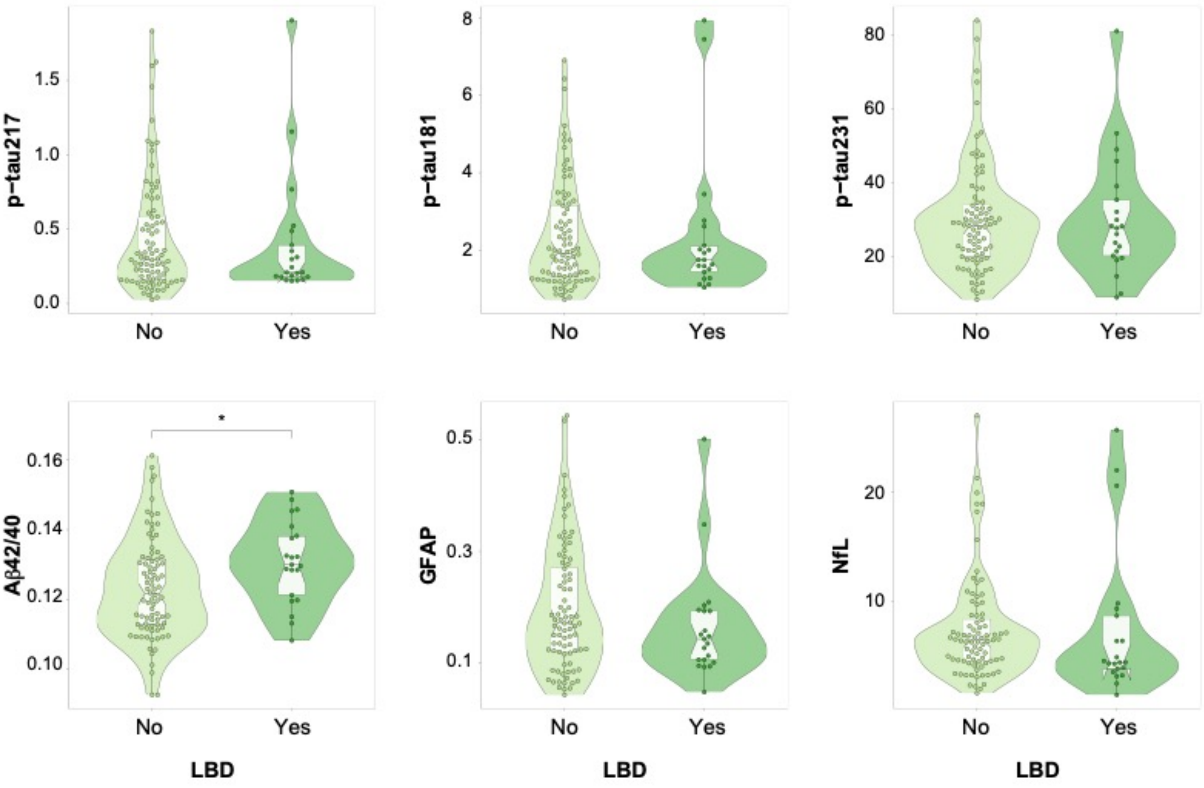

Appendix Figure S7 Plasma levels by presence or absence of LBD

Groups were compared using a Kruskal-Wallis test. Only Aβ42/40 differences remained significant after adjusting for covariates (Appendix Table S9).

*** p<0.001; ** p<0.010 ; * p<0.050

Abbreviations: Aβ, amyloid-β; GFAP, glial fibrillary acidic protein; LBD, Lewy body disease; NfL, neurofilament light; p-tau, phosphorylated tau.

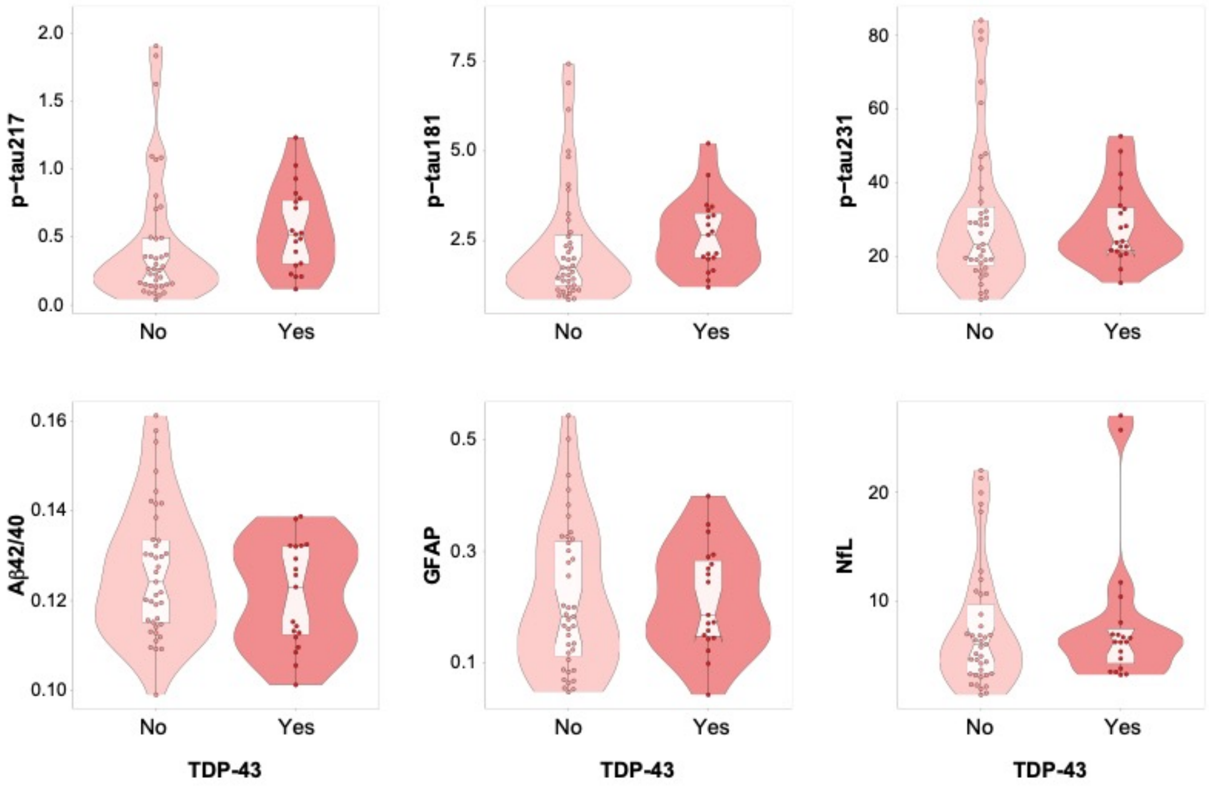

Appendix Figure S8 Plasma levels by presence or absence of TDP-43 pathology

Groups were compared using a Kruskal-Wallis test. None of these differences remained significant after adjusting covariates (Appendix Table S10).

*** p<0.001; ** p<0.010 ; * p<0.050

Abbreviations: Aβ, amyloid-β; GFAP, glial fibrillary acidic protein; NfL, neurofilament light; p-tau, phosphorylated tau, TDP-43, TAR DNA binding protein 43.

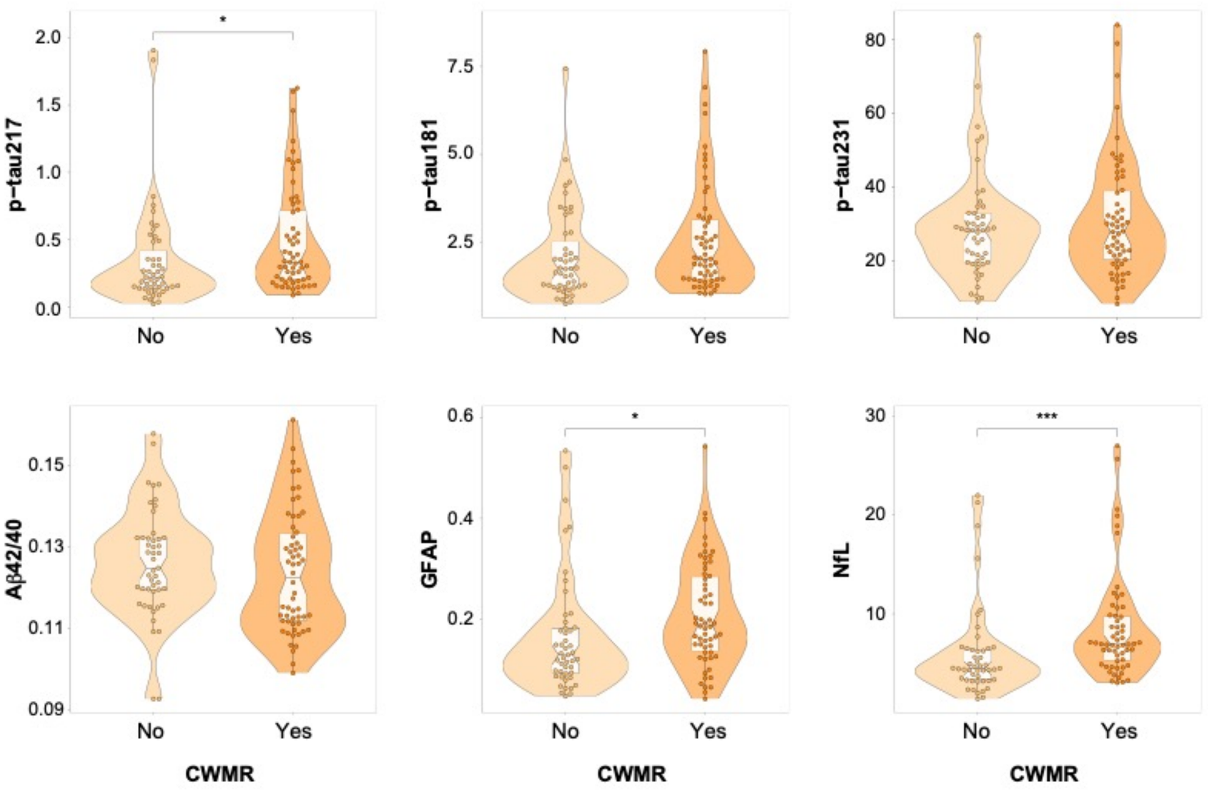

Appendix Figure S9 Plasma levels by presence or absence of CWMR

Groups were compared using a Kruskal-Wallis test. Differences of plasma p-tau217 and NfL levels remained significant after adjusting for covariates (Appendix Table S7).

*** p<0.001; ** p<0.010 ; * p<0.050

Abbreviations: Aβ, amyloid-β; CWMR, cerebral white matter rarefaction; GFAP, glial fibrillary acidic protein; NfL, neurofilament light; p-tau, phosphorylated tau.

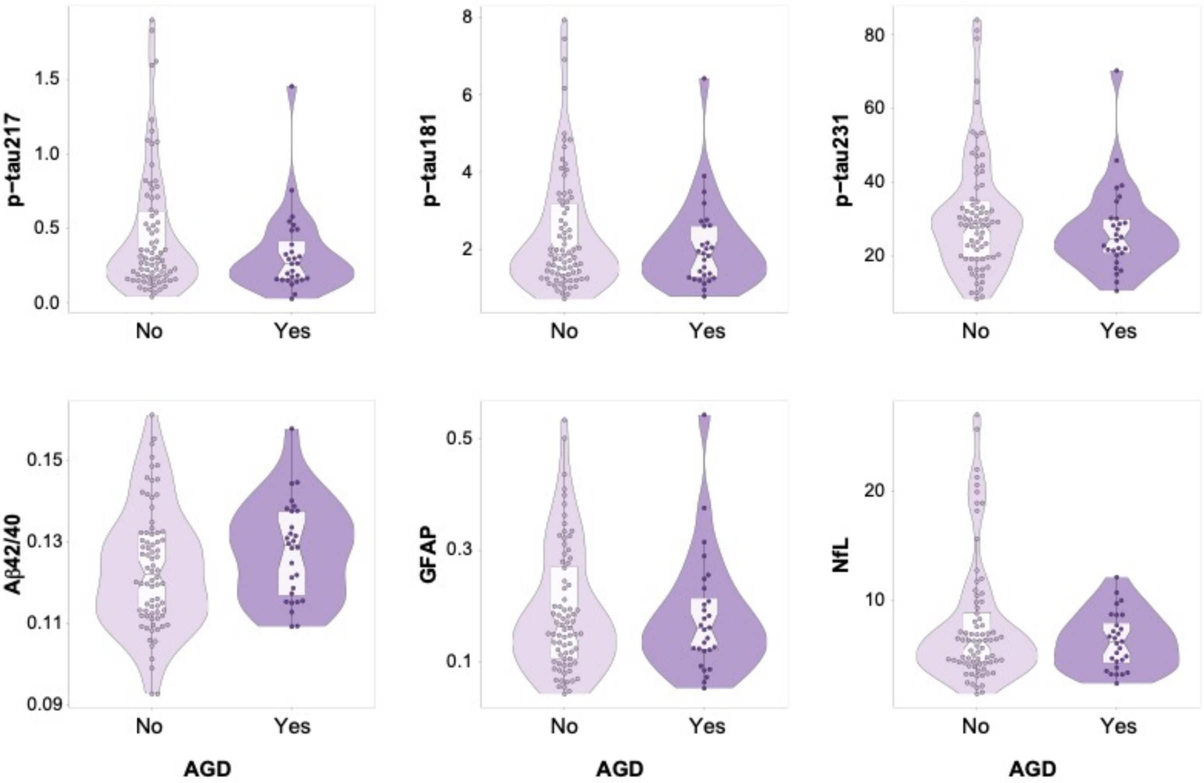

Appendix Figure S10 Plasma levels by presence or absence of AGD

Groups were compared using a Kruskal-Wallis test. Differences of plasma p-tau217 levels became significant after adjusting for covariates (Appendix Table S11).

*** p<0.001; ** p<0.010 ; * p<0.050

Abbreviations: Aβ, amyloid-β; AGD, argyrophilic grain disease; GFAP, glial fibrillary acidic protein; NfL, neurofilament light; p-tau, phosphorylated tau.

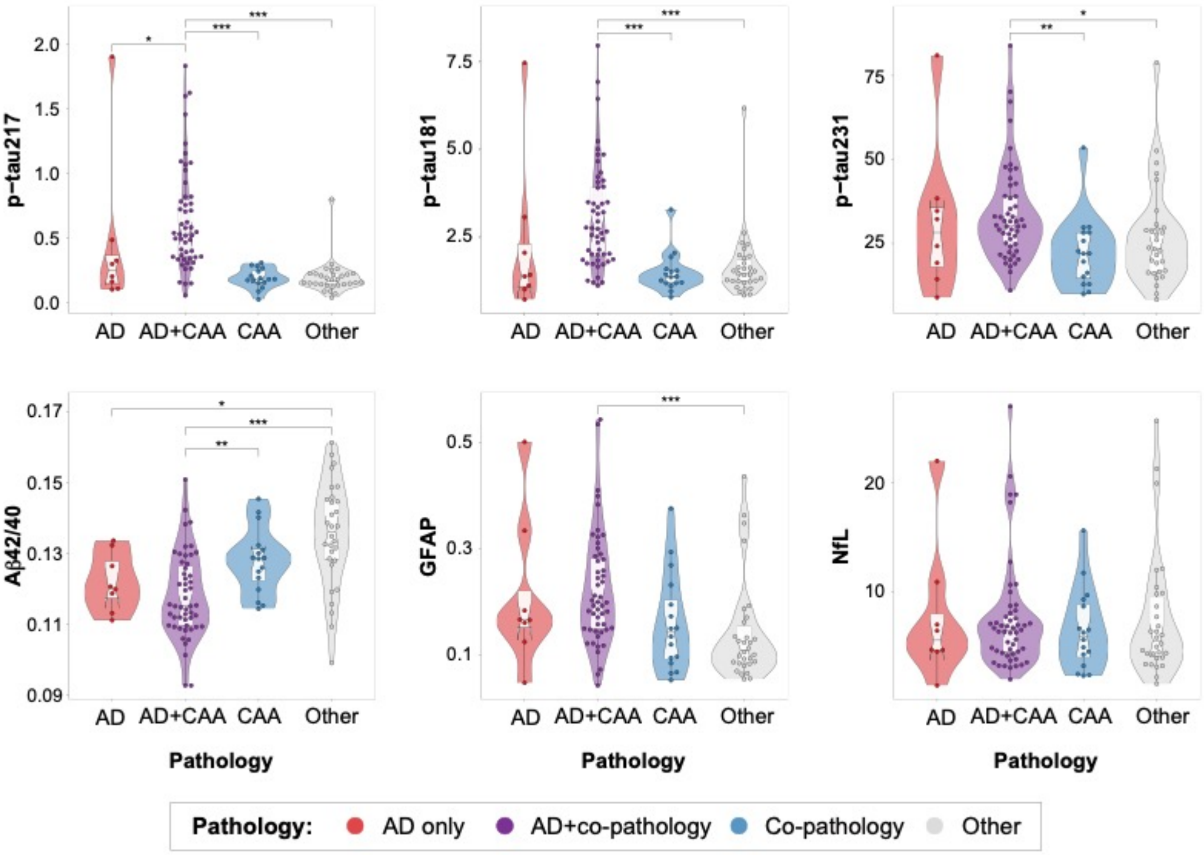

Appendix Figure S11 Plasma levels by presence of AD pathology and/or CAA

Groups were compared using a Kruskal-Wallis test and *post-hoc* comparisons among groups were performed using Wilcoxon's test. P-values were corrected for multiple comparisons using FDR. We compared all groups against the AD pathology only group, and all groups against the AD and CAA pathologies group.

*** p<0.001; ** p<0.010; * p<0.050

Abbreviations: Aβ, amyloid-β; CAA, cerebral amyloid angiopathy; FDR, false discovery rate; GFAP, glial fibrillary acidic protein; NfL, neurofilament light; p-tau, phosphorylated tau.

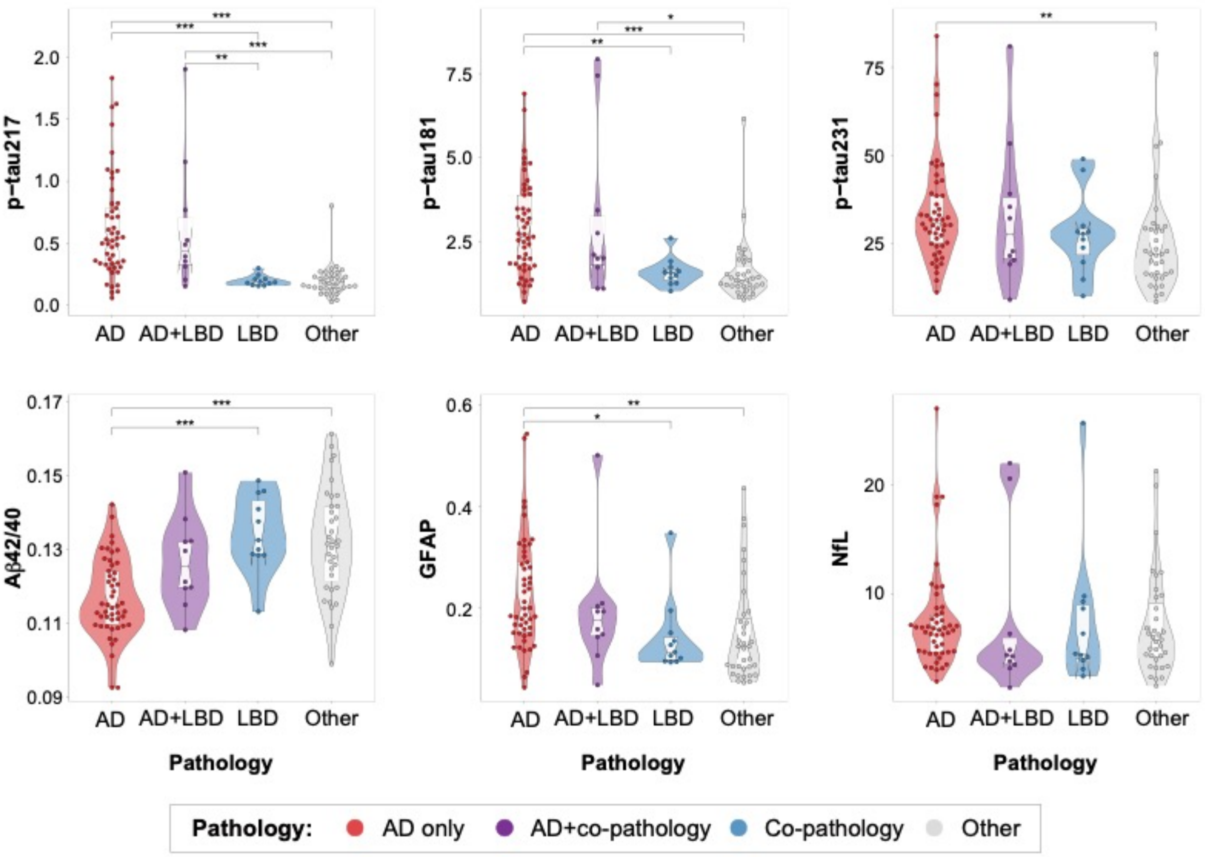

Appendix Figure S12 Plasma levels by presence of AD pathology and/or LBD

Groups were compared using a Kruskal-Wallis test and *post-hoc* comparisons among groups were performed using Wilcoxon's test. P-values were corrected for multiple comparisons using FDR. We compared all groups against the AD pathology only group, and all groups against the AD and LBD pathologies group.

*** p<0.001; ** p<0.010; * p<0.050

Abbreviations: Aβ, amyloid-β; FDR, false discovery rate; GFAP, glial fibrillary acidic protein; LBD, Lewy body disease; NfL, neurofilament light; p-tau, phosphorylated tau.

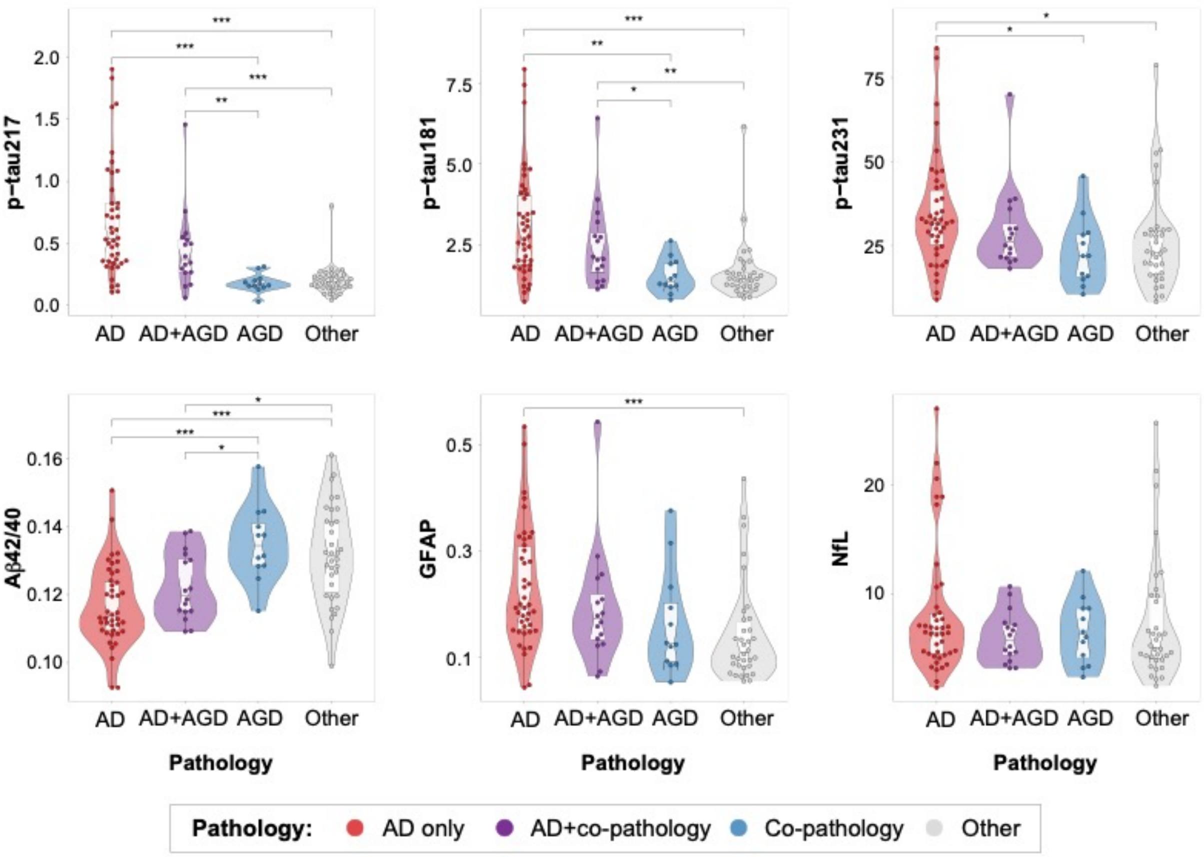

Appendix Figure S13 Plasma levels by presence of AD pathology and/or AGD

Groups were compared using a Kruskal-Wallis test and *post-hoc* comparisons among groups were performed using Wilcoxon's test. P-values were corrected for multiple comparisons using FDR. We compared all groups against the AD pathology only group, and all groups against the AD and AGD pathologies group.

*** p<0.001; ** p<0.010; * p<0.050

Abbreviations: Aβ, amyloid-β; AGD, AGD, argyrophilic grain disease; FDR, false discovery rate; GFAP, glial fibrillary acidic protein; NfL, neurofilament light; p-tau, phosphorylated tau.

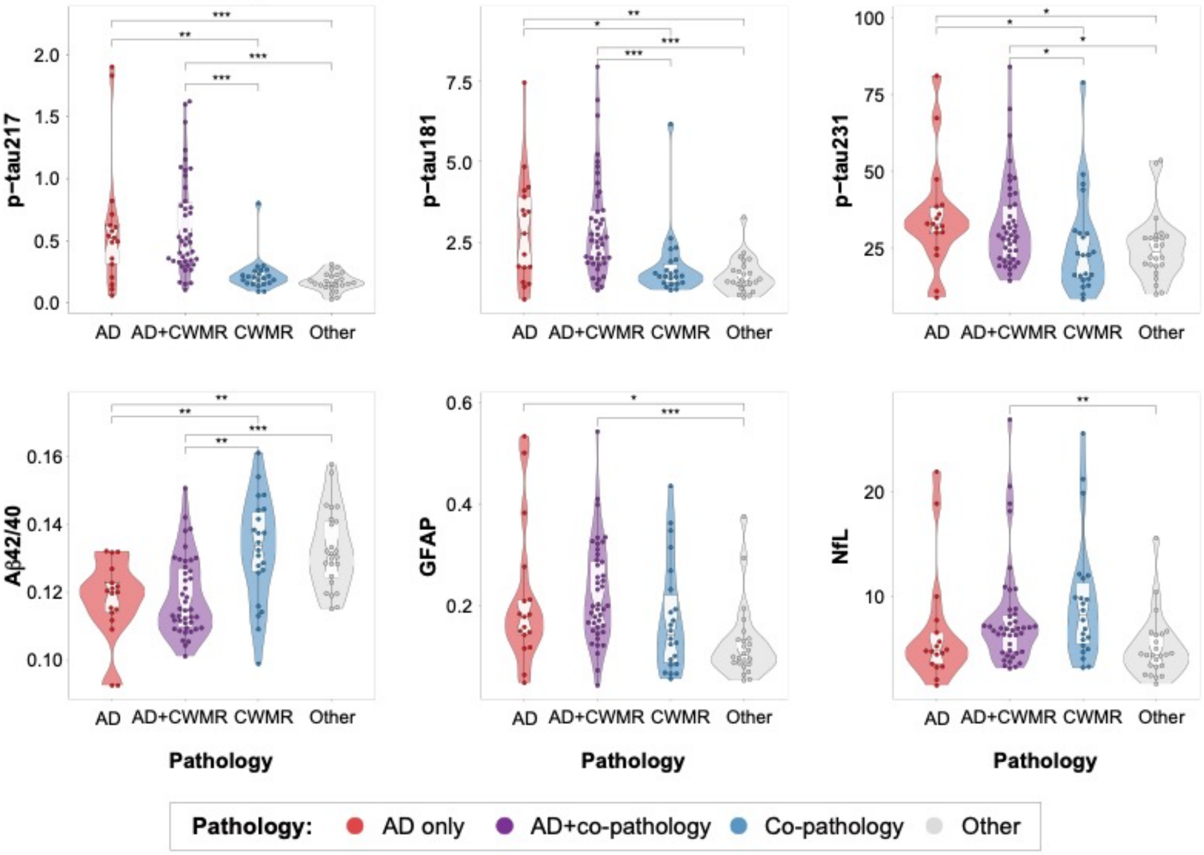

Appendix Figure S14 Plasma levels by presence of AD pathology and/or CWMR

Groups were compared using a Kruskal-Wallis test and *post-hoc* comparisons among groups were performed using Wilcoxon's test. P-values were corrected for multiple comparisons using FDR. We compared all groups against the AD pathology only group, and all groups against the AD and CWMR pathologies group.

*** p<0.001; ** p<0.010; * p<0.050

Abbreviations: Aβ, amyloid-β; CWMR, cerebral white matter rarefaction; FDR, false discovery rate; GFAP, glial fibrillary acidic protein; NfL, neurofilament light; p-tau, phosphorylated tau.

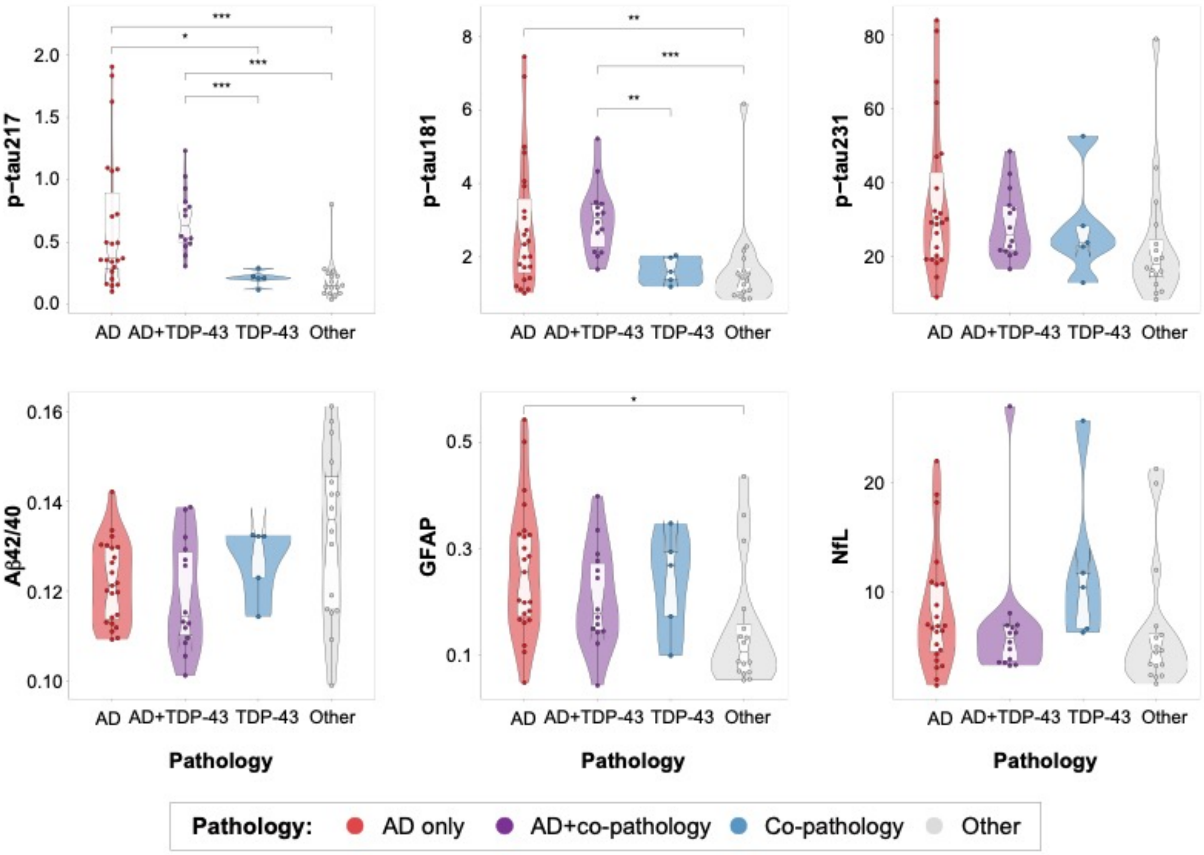

Appendix Figure S15 Plasma levels by presence of AD pathology and/or TDP-43

Groups were compared using a Kruskal-Wallis test and *post-hoc* comparisons among groups were performed using Wilcoxon's test. P-values were corrected for multiple comparisons using FDR. We compared all groups against the AD pathology only group, and all groups against the AD and TDP-43 pathologies group.

*** p<0.001; ** p<0.010; * p<0.050

Abbreviations: Aβ, amyloid-β; AGD, FDR, false discovery rate; GFAP, glial fibrillary acidic protein; NfL, neurofilament light; p-tau, phosphorylated tau; TDP-43, TDP-43, TAR DNA binding protein 43.

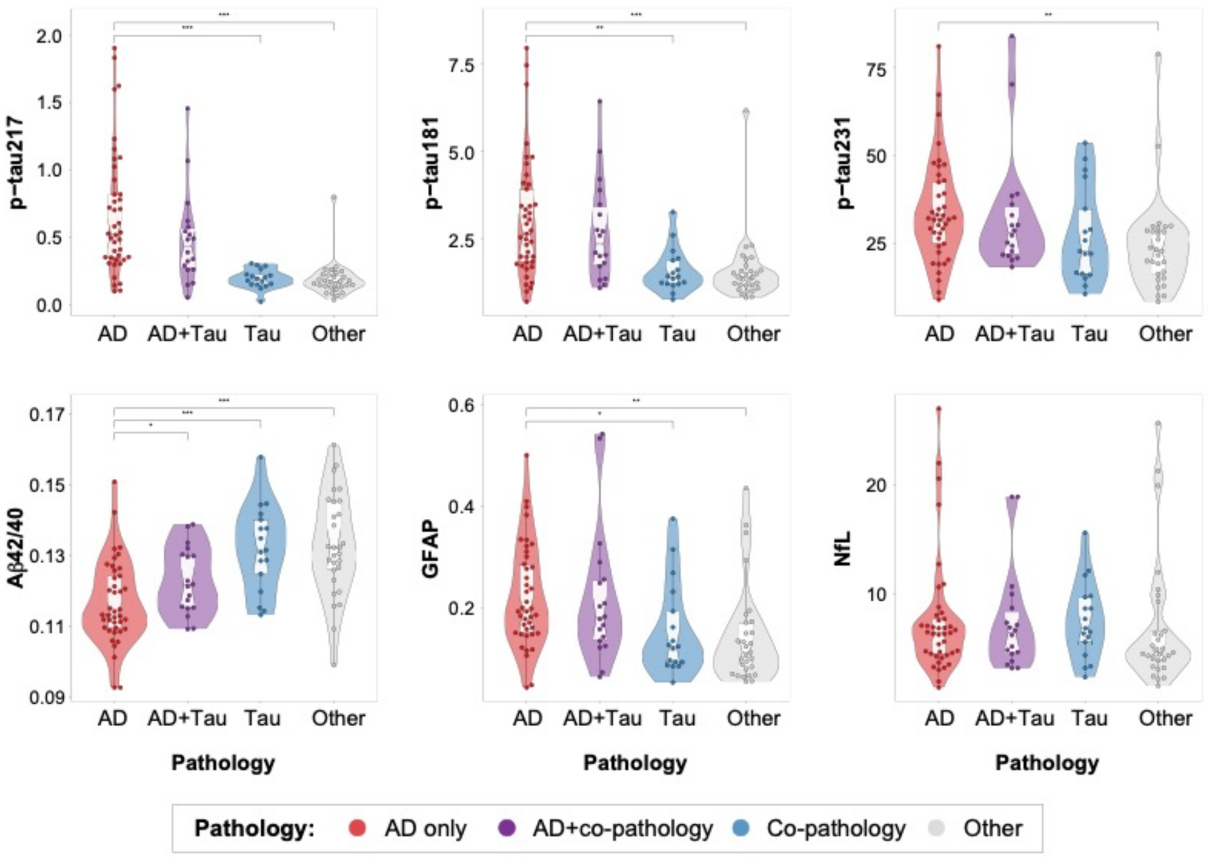

Appendix Figure S16 Plasma levels by presence of AD pathology and/or primary tauopathies

Groups were compared using a Kruskal-Wallis test and *post-hoc* comparisons among groups were performed using Wilcoxon's test. P-values were corrected for multiple comparisons using FDR. We compared all groups against the AD pathology only group, and all groups against the AD and primary tauopathies group.

*** $p<0.001$; ** $p<0.010$; * $p<0.050$

Abbreviations: Aβ, amyloid-β; AGD, FDR, false discovery rate; GFAP, glial fibrillary acidic protein; NfL, neurofilament light; p-tau, phosphorylated tau.

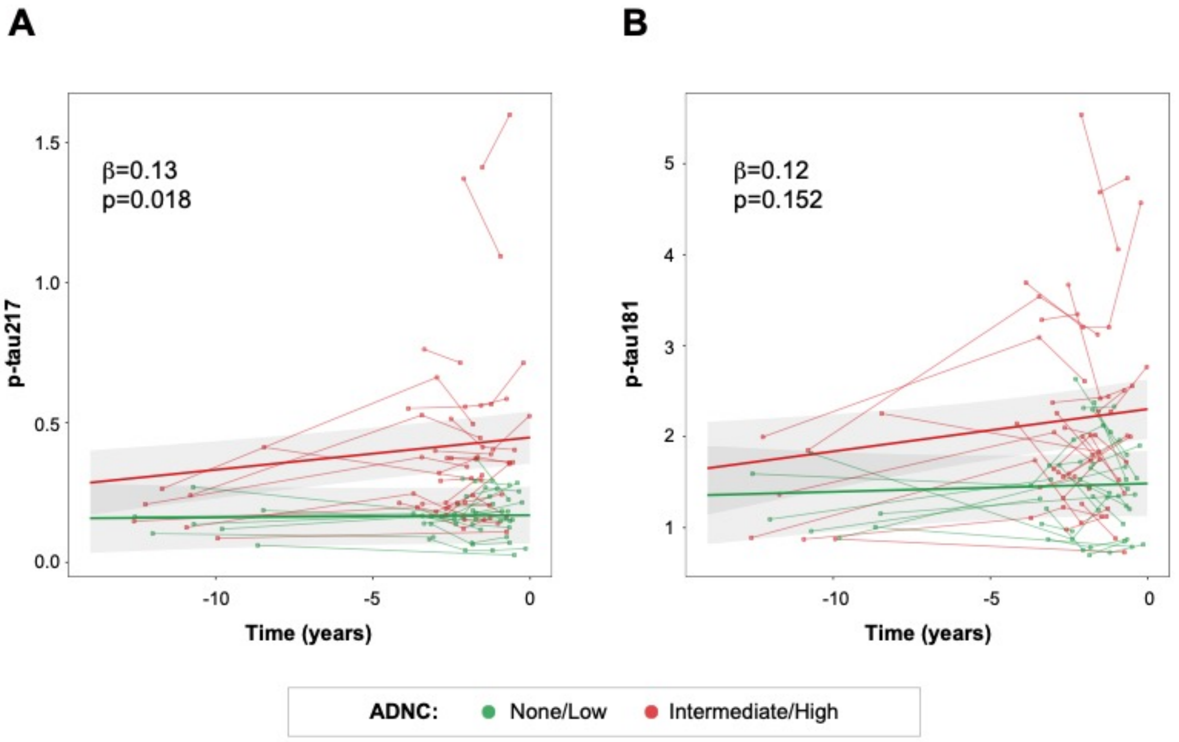

Appendix Figure S17 Associations between longitudinal changes of plasma biomarkers and presence of ADNC at death

Bold lines represent mean longitudinal changes of plasma p-tau217 (A) and plasma p-tau181 (B) by ADNC groups at death. Linear mixed effect models were used to derive these associations in independent models including: age at baseline, and sex as covariates using and random intercepts and fixed time-slopes. Intercept was fixed at time of death. ADNC was dichotomized as negative (none/low) or positive (intermediate/high). ADNC*time interaction standardized betas and p-values are shown in the figure.

Abbreviations: ADNC, Alzheimer's disease neuropathologic change; p-tau, phosphorylated tau.
