# Supplementary material for: Specific associations between plasma biomarkers and postmortem amyloid plaque and tau tangle loads
Source: EMBO Mol Med. 2023 Mar 13;15(5):e17123. doi: 10.15252/emmm.202217123 (PMC10165361; doi:10.15252/emmm.202217123)
Supplement: Supplementary file 2 [file EMMM-15-e17123-s002.pdf]

EMBO Molecular Medicine

Specific associations between plasma biomarkers and post-mortem amyloid plaque and tau tangle loads

Gemma Salvado, Rik Ossenkoppele, Nicholas Ashton, Thomas Beach, Geidy Serrano, Eric Reiman, Henrik Zetterberg, Niklas Mattsson-Carlgren, Shorena Janelidze, Kaj Blennow, and Oskar Hansson
DOI: 10.15252/emmm.202217123

Corresponding authors: Oskar Hansson (oskar.hansson@med.lu.se) , Gemma Salvado (gemma.salvado@med.lu.se)

Review Timeline:

Submission Date:	31st Oct 22
Editorial Decision:	30th Nov 22
Revision Received:	21st Dec 22
Editorial Decision:	23rd Jan 23
Revision Received:	21st Feb 23
Accepted:	21st Feb 23

Editor: Jingyi Hou

Transaction Report:

(Note: With the exception of the correction of typographical or spelling errors that could be a source of ambiguity, letters and reports are not edited. Depending on transfer agreements, referee reports obtained elsewhere may or may not be included in this compilation. Referee reports are anonymous unless the Referee chooses to sign their reports.)

30th Nov 2022

Dear Dr. Hansson,

Thank you again for submitting your work to EMBO Molecular Medicine. We have now heard back from three referees who agreed to evaluate your manuscript. As you will see from the reports below, the referees acknowledge the potential interest of the study. However, they raise a series of concerns, which should be addressed in a major revision of the current manuscript.

The referees' recommendations are relatively straightforward, so there is no need to reiterate their comments. In particular, Referee #3 raised significant concerns about the statistical approach (linear regression) applied and thought the current analysis did not support the study's conclusions. During the pre-decision cross-commenting process (in which the referees are given a chance to make additional comments, including on each other's reports), Referee #1 added, "Referee #3 is right in their statement that the obtained neuropathological variables are not normally distributed, and linear regression is not appropriate for the data. There are several open questions with this manuscript, and the revised version will have to be carefully re-evaluated." In light of the comments of Referees #1 and #3, we would ask you to carefully address the concerns about statistics raised by Referee #3.

Other issues raised by the referees need to be satisfactorily addressed as well. Please feel free to contact me in case you would like to discuss in further detail any of the issues raised by the referees. We would welcome the submission of a revised version within three months for further consideration. Please note that EMBO Molecular Medicine strongly supports a single round of revision. As acceptance or rejection of the manuscript will depend on another round of review, your responses should be as complete as possible.

EMBO Molecular Medicine has a "scooping protection" policy, whereby similar findings that are published by others during review or revision are not a criterion for rejection. Should you decide to submit a revised version, I do ask that you get in touch after three months if you have not completed it to update us on the status.

We are aware that many laboratories cannot function at full efficiency during the current COVID-19/SARS-CoV-2 pandemic and have therefore extended our "scooping protection policy" to cover the period required for a full revision to address the experimental issues. Please let me know should you need additional time and also if you see a paper with related content published elsewhere.

Please read below for important editorial formatting and consult our author's guidelines for proper formatting of your revised article for EMBO Molecular Medicine.

I look forward to receiving your revised manuscript soon.

Kind regards,
Jingyi

Jingyi Hou
Editor
EMBO Molecular Medicine

*****

***** Reviewer's comments *****

Referee #1 (Comments on Novelty/Model System for Author):

Salvado et al compared associations between multiple plasma biomarkers (i.e. p-tau181, p-tau217, p-tau231, Aβ42/40, GFAP and NfL) and neuropathologic measures of tau and amyloid pathology in an autopsy-based cohort of patients with antemortem blood samples available for analyses. They demonstrated that plasma p-tau181 and p-tau217 are specific markers of both tau tangles and amyloid plaques, whereas the Aβ42/40 ratio and p-tau231 levels are markers strictly associated with plaques and GFAP with only tangles. The combination of plasma p-tau217 and the Aβ42/40-ratio gave the highest accuracy for predicting amyloid plaque load, while P-tau217 alone seemed to be sufficient to predict tangle load. Furthermore, the longitudinal changes of p-tau217, but not those of p-tau181, were significantly associated with presence of AD pathology at death, especially tangle load. The results suggest that high-performing assays of plasma p-tau217 and Aβ42/40 might be a sufficient biomarker combination to assess Alzheimer's-related pathology in vivo.

The current work goes beyond the current knowledge, since a head-to head comparison of the plasma biomarkers measured by high performing assays and their correlation to gold standard neuropathological measures had been missing. Previous studies only focused on the relation of single biomarkers to amyloid and tau pathologies, which did not allow for interpretation of their independent associations with these two pathological measures.

Referee #1 (Remarks for Author):

Salvado et al compared associations between multiple plasma biomarkers (i.e. p-tau181, p-tau217, p-tau231, Aβ42/40, GFAP and NfL) and neuropathologic measures of tau and amyloid pathology in an autopsy-based cohort of patients with antemortem blood samples available for analyses. They demonstrated that plasma p-tau181 and p-tau217 are specific markers of both tau tangles and amyloid plaques, whereas the Aβ42/40 ratio and p-tau231 levels are markers strictly associated with plaques and GFAP with only tangles. The combination of plasma p-tau217 and the Aβ42/40-ratio gave the highest accuracy for predicting amyloid plaque load, while P-tau217 alone seemed to be sufficient to predict tangle load. Furthermore, the longitudinal changes

of p-tau217, but not those of p-tau181, were significantly associated with presence of AD pathology at death, especially tangle load. The results suggest that high-performing assays of plasma p-tau217 and Aβ42/40 might be a sufficient biomarker combination to assess Alzheimer's-related pathology in vivo.

The current work goes beyond the current knowledge, since a head-to head comparison of the plasma biomarkers measured by high performing assays and their correlation to gold standard neuropathological measures had been missing. Previous studies only focused on the relation of single biomarkers to amyloid and tau pathologies, which did not allow for interpretation of their independent associations with these two pathological measures.

Items to be addressed in a revised version of the manuscript:
1. Overall, the writing is from highly specialized perspective and lacks efforts to convey information to a broader neurological readership.

2. Although most relevant details are provided in the methods section, the readability of the paper suffers severely, since relevant information is not provided in the linear flow of the manuscript, with methods being placed only at the end.
a. E.g. on page 6, second section of the results, association between plasma biomarkers and "total amount of plaques and tangles" are reported. At this point, the reader needs to know how these parameters have been determined. Certainly the expression "total amount" is misleading, since the authors have not stereologically determined the total amount of plaques and tangles in the brain. The readers require at this point in the manuscript an operationalized definition of these parameters, e.g. Braak and Brak Tau NFT stage, CERAD a-beta stage, or (semi)quantitative evaluation of plaques / tangles in one region / in several regions / in which regions?
b. The results section reports groups defined by participants with none/low AD-neuropathological change (ADNC) and 59 participants with intermediate/high ADNC, whereas table 1 reports groups as ADNC-neg. vs. ADNC-pos. please seek consistency throughout the manuscript. Provide a reference, which the classification is based upon in the main body of the manuscript, not only in the results section at the end.
c. Also, the ADNC score incorporating both tau and a-beta pathology needs to be briefly introduced at the first occurrence in the manuscript.
3. One major weakness is the absence of an independent cohort to verify the key findings of the current work independently. If no independent sample is available, could statistical random splits of the current sample be used to validate the findings? The absence of a confirmatory sample needs to be emphasized, if it cannot be overcome.

4. The denomination of the parameters reported in Table 1 is not comprehensible to the general readership and requires specifications, e.g.:
a. "Time between blood and death", authors probably mean time between plasma sampling and death / blood and brain donation.
b. "CERAD moderate / frequent", the authors probably mean CERAD neuritic plaque frequency score
c. "Braak stage", the authors probably mean Braak and Braak stages of Tau-NFT stages.
d. "CAA", probably Thal stages
e. LBD, TDP, CWMR, AGD: the table needs to be self-explaining without a deep dive into the methods section, presently the table is incomprehensible.
f. pTau, a-beta, GFAP, Nf: Plasma concentrations
g. propose to have subheadings: demongraphic data / neuropathological data / plasma biomarker data
h. Please provide specific and comprehensible information for all parameters, provide references for the neuropath staging systems applied.

5. Table 2: please also provide a correlation analysis for the neuropathological plaque and tangle pathology in your sample. If factors A (e.g. plaques) and B (e.g. tangles) are significantly correlated with one another, then other factors Ci (e.g. p-tau181, p-tau217) causally related to A can also correlate with B in that particular sample irrespective of a causal association (indirect association). This is a conceptual aspect in the current study which requires adequate attention.

6. While all analyses are correlative in nature, the authors implicitly suggest causal relationships between neuropathological parameters and plasma biomarker levels, e.g. heading of Figure legend 2. The authors need to interpret their correlative data in one single sample with due care. Interpretations suggesting causal relationship need to be avoided.

7. Recent evidence suggests that microglial activation is involved in the spread of tau tangles over the neocortex in Alzheimer's disease (AD) and an interaction between Aβ and activated microglia sets the pace for tau spread across Braak stages (Pascoal et al, 2021, doi:10.1038/s41591-021-01456-w). Why did the colleagues not further check for neuroinflammation?

8. Another important issue concerns comparisons to other neurodegenerative diseases, which is missing in the current work. Thus, the conclusion of specificity of the associations in the last paragraph of the discussion needs to be tempered down.

9. The colleagues discuss the role of p-tau231, both as a CSF and a plasma biomarker, as a marker for early AD stages and associated amyloid pathology (Ashton et al, 2021; Meyer et al, 2022; Milà-Alomà, 2022; Smirnov et al, 2022; Suárez-calvet et al, 2020). Why was p-Tau 231 not included in the longitudinal investigation? The investigation of earlier blood samples might

enhance understanding of p-Tau231 as a possible early marker of AD.
Minor issues:
- Page last but one paragraph: wrong word order: ... importance the use of high performing assays is of utmost ...
- Figure 1: indication of the same parameters to the left AND at the top of the panels is redundant and confusing. The plaque and tangle unit (range 0-15) requires an explanation on the figure / in the legend, to enable the reader understanding without diving deep into the methods section.
- Figure 2: legend indicated plaques being green, tangles being orange, but figure shows blue and brown
- Suppl. Figures: the abbreviations seem to be partly inappropriate, e.g. CAA is not on display. CERAD does not refer to the consortium, but the corresponding staging system. The general reader needs to be told that CERAD and Braak classifications relate to tau and amyloid beta, NFT and plaque pathology, respectively.

Referee #2 (Comments on Novelty/Model System for Author):

see comments to the authors

Referee #2 (Remarks for Author):

In this neuropathological validation study of Alzheimer blood biomarkers in approx. 100 cases of the Arizona Brain Bank Salvado et al demonstrate a continuous relationship between 181phosphotau and 217phosphotau measures and amyloid plaque load as well as neurofibrillary tangle load. A strong relationship was also found for Aβ42/40 and amyloid plaque load. As a further novelty, the authors demonstrate a relationship between GFAP and neurofibrillary tangle load. The data were also analysed using a classification approach and confirm the diagnostic validity of 217phosphotau (plus Aβ42/40, see below) against an AD diagnosis based on CERAD or NIA-AA criteria. GFAP shows a surprisingly high diagnostic accuracy against Braak stageing. Overall NFL performs poorly as a diagnostic tool for detecting AD pathological changes. It is also of interest that the authors compared between each of the AD stages, e.g. none vs low neuritic plaque load, and observed significant differences for some of the biomarkers.
Needless to say, this type of neuropathological validation is essential and a critical step for research and clinical use of blood biomarkers. The data constitute a central piece of evidence, complementing prior studies that were mostly based on validation against molecular imaging biomarkers. The study design is relatively standard. There is a relatively long interval between blood sampling and neuropathological verification but this is hard (although not entirely impossible) to avoid outside the context of industry-sponsored trials. The analysis is conducted in a careful, detailed and rigorous manner and the results speak for themselves. They are comprehensively illustrated by a set of clear figures. This may become a landmark paper as far as this is predictable.
Major comment
1. It would be of interest to also report whether any of the biomarkers discriminate between CAA without vs with AD parenchymal aggregates. This is of clinical relevance and would also shed further insight in the neuropathological basis for the plasma biomarkers. Likewise for Lewy body pathology with vs without AD neuropathology.
2. P 11 top paragraph: the interpretation that 217phosphotau is the best biomarker should be qualified, as this may also depend on the exact assay used for 181phosphotau. The authors may want to add a qualification in the sense of 'for the assays tested in the current study'. They do so in one of the subsequent paragraphs.
3. The interpretation of the relationship between GFAP and tangle load: the authors may want to discuss the possibility of an indirect (eg as a microglial or astrocytic marker) vs a direct relationship.
4. Table 4: for the longitudinal samples, the beta coefficients are relatively low and the difference between 217phosphotau and 181phosphotau small, at least for the interaction with tangle load and the interaction with ADNC. This contrasts with the relatively strong conclusions in the discussion that the former is superior for tracking disease progression in trials. The design of the longitudinal study does not allow strong conclusions and the differences between 217phosphotau and 181phosphotau are overstated. The authors may consider to remove this component as the strength of evidence is substantially weaker than for the other components of the paper.
Minor comments:
1. Based on figure 4, the parsimonious model seems equivalent to the model with 217phosphotau alone, and there does not seem to be significant added value of combining with Abeta42/40. While numerically the parsimonious model has the highest accuracy, the difference with 217phophotau alone seems negligible. The authors argue that there is a difference in AIC but the superiority of the parsimonious model vs the 217phosphotau alone could be tested with DeLong test and it would be surprising if there is a significant difference. The difference in AIC reported on p 9 also seems very small.
Very minor comment:
1. In figure 3 I suggest to drop the figure titles since it may be confusing whether the title belongs to the figure above or below, it is clear enough from the y axis title what is plotted.

Referee #3 (Comments on Novelty/Model System for Author):

As described in the comments to the authors, the main result of the paper is based on linear regression models between plasma marker levels and plaque and tangle severity at postmortem (Figure 2 and Table 2). However, plaque and tangle loads are obtained by descriptive measures (or as stated by the authors "semi-quantitative measures") of severity that were converted into 0-3 scores in each region and then added across the 5 regions investigated. Thus, the pathology loads derive from an ordinal qualitative assessment, rather than a quantitative "continuous" evaluation (i.e. counting, AI quantification). This makes the statistical approach applied (linear regression) not appropriate for the data and research question. Indeed, the obtained variables are not normally distributed, and the distribution of data points in Figure 1, especially for plaques, is highly likely to lead to spurious correlations - partially due to the combination of groups that differ in the variables of interest (i.e. none/low vs intermediate/high ADNC). If the results of these analyses are excluded, the novelty of the work decreases significantly.

Referee #3 (Remarks for Author):

The authors investigate the association between dementia-related plasma markers and pathology at postmortem in patients with low to high ADNC. They describe that specific plasma markers are associated with only amyloid (Aβ42/40 and p-tau231), only tau pathology (plasma GFAP) and, some with both pathologies (p-tau217 and p-tau181). They suggest that the combination of ptau217 and the Aβ42/40 ratio may be useful to predict amyloid pathology, while p-tau217 alone may be a good marker to detect tau pathology.

Studies on plasma markers in dementia have become increasingly important to validate new tools that could be implemented in clinical practice and trials. The research question is very important and the dataset includes patients with different levels of ADNC severity. However, the analyses may not support the conclusions of the study completely.

The main result of the paper is based on linear regression models between plasma marker levels and plaque and tangle severity at postmortem (Figure 2 and Table 2). However, plaque and tangle loads are obtained by descriptive measures (or as stated by the authors "semi-quantitative measures") of severity that were converted into 0-3 scores in each region and then added across the 5 regions investigated. Thus, the pathology loads derive from an ordinal qualitative assessment, rather than a quantitative "continuous" evaluation (i.e. counting, AI quantification). This makes the statistical approach applied (linear regression) not appropriate for the data and research question. Indeed, the obtained variables are not normally distributed, and the distribution of data points in Figure 1, especially for plaques, is highly likely to lead to spurious correlations - partially due to the combination of groups that differ in the variables of interest (i.e. none/low vs intermediate/high ADNC). If the results of these analyses are excluded, the novelty of the work decreases significantly.

Another potentially novel part of the paper is the use of longitudinal plasma markers to predict pathology. The authors applied a linear mixed effect model with "time" as predictor, however, here it should be clarified that the intercept should be fixed on the date of death rather than the first blood sample available. As presented by the authors in Supp Figure 10, time is not the simple interval between blood samples but the time interval between each visit and death. The interval between visits and the number of samples available for each individual seem to vary a lot between participants (Supplementary Table 13: Time points, median[range] 2 [2-5]; Time difference, days, mean(SD) 1,378 (1,357)). Most of the participants are III/IV Braak stage (83.3%). These are all factors that should be discussed in the interpretation of the results. The authors describe a significant association between longitudinal changes of p-tau217 and presence of ADNC at death. However, upon visual inspection of the data in Supp Figure 10, this may be driven by two outliers (left panel, top right), and by a subsample of participants with longer intervals between visits.

Other comments:

Information on whether pathology evaluation was performed by multiple experts is not reported, and potential inter-rater agreement or limitations are not tested or described.

The results are based on multiple comparisons between different plasma markers and their relationship with pathology, but corrections for this have not been applied.

Correlations were found across the whole ADNC spectrum, but it is not clear whether the associations of tangles and plaques with blood markers differ within ADNC specific groups?

ADNC negative/positive groups were defined by pathology examination. Did these reflect AD-biomarker positivity/negativity in vivo, especially in MCI?

Other pathologies have been investigated in post-mortem examination (i.e. CAA, TDP-43, LBD and AGD). Were primary tauopathies such as CBD, PSP, Pick's disease not considered given that participants with AD, MCI and other neurodegenerative diseases were described in the method section?

In Table 2, parameters for other factors included in the model should be added (ie age sex etc).

Referee #1 (Remarks for Author):

Salvado et al compared associations between multiple plasma biomarkers (i.e. p-tau181, p-tau217, p-tau231, Aβ42/40, GFAP and NfL) and neuropathologic measures of tau and amyloid pathology in an autopsy-based cohort of patients with antemortem blood samples available for analyses. They demonstrated that plasma p-tau181 and p-tau217 are specific markers of both tau tangles and amyloid plaques, whereas the Aβ42/40 ratio and p-tau231 levels are markers strictly associated with plaques and GFAP with only tangles. The combination of plasma p-tau217 and the Aβ42/40-ratio gave the highest accuracy for predicting amyloid plaque load, while P-tau217 alone seemed to be sufficient to predict tangle load. Furthermore, the longitudinal changes of p-tau217, but not those of p-tau181, were significantly associated with presence of AD pathology at death, especially tangle load. The results suggest that high-performing assays of plasma p-tau217 and Aβ42/40 might be a sufficient biomarker combination to assess Alzheimer's-related pathology in vivo.

The current work goes beyond the current knowledge, since a head-to head comparison of the plasma biomarkers measured by high performing assays and their correlation to gold standard neuropathological measures had been missing. Previous studies only focused on the relation of single biomarkers to amyloid and tau pathologies, which did not allow for interpretation of their independent associations with these two pathological measures.

We thank the reviewer for their kind words.

Items to be addressed in a revised version of the manuscript:
1. Overall, the writing is from highly specialized perspective and lacks efforts to convey information to a broader neurological readership.
We thank the reviewer for this comment. We have now modified the text to better accommodate a broader audience. For instance, we have added more information about the measures and analyses at the beginning of each section. Further, we have expanded the clinical implications of our results across the manuscript. We feel that this has substantially improved the quality of our manuscript.

2. Although most relevant details are provided in the methods section, the readability of the paper suffers severely, since relevant information is not provided in the linear flow of the manuscript, with methods being placed only at the end.
We agree with the reviewer that the format was not adequate to allow an easy read. We have improved this following reviewer's comments and, in summary, adding more information about the methods every time we use a new variable or analysis.

a. E.g. on page 6, second section of the results, association between plasma biomarkers and "total amount of plaques and tangles" are reported. At this point, the reader needs to know how these parameters have been determined. Certainly the expression "total amount" is misleading, since the authors have not stereologically determined the total amount of plaques and tangles in the brain. The readers require at this point in the manuscript an operationalized definition of these parameters, e.g. Braak and Brak Tau NFT stage, CERAD a-beta stage, or (semi)quantitative evaluation of plaques / tangles in one region / in several regions / in which regions?
We have included a brief explanation of these measures in the results section (page 6): "Plaque and tangle loads were measured in a semi-quantitative scale that ranged from 0 to 3 in five different regions (Mirra et al, 1991), and we added up these regional measures to obtain a total score (0 to 15) for each pathology."
Page 9:

“Then we also looked at differences at pathological scales specific of amyloid (Consortium to establish a registry for Alzheimer's disease, [CERAD]) (Mirra et al, 1991) and tau (Braak staging) (Braak & Braak, 1991) pathologies.”

b. The results section reports groups defined by participants with none/low AD-neuropathological change (ADNC) and 59 participants with intermediate/high ADNC, whereas table 1 reports groups as ADNC-neg. vs. ADNC-pos. please seek consistency throughout the manuscript. Provide a reference, which the classification is based upon in the main body of the manuscript, not only in the results section at the end.
We have changed this in Table 1 and included a reference in the text as suggested by the reviewer (see text next question).

c. Also, the ADNC score incorporating both tau and a-beta pathology needs to be briefly introduced at the first occurrence in the manuscript.
We have included a brief description at the beginning of the results section (page 6):
“These participants were categorized as having significant AD pathology (n=59) or not (n=46) based on the Alzheimer’s disease neuropathologic change (ADNC) scale, in which both amyloid and tau pathologies are accounted (Montine et al, 2012). Participants with significant AD pathology were those that an intermediate or high scores in the ADNC scale, whereas those with none or low scores were classified as having non-significant AD pathology.”

3. One major weakness is the absence of an independent cohort to verify the key findings of the current work independently. If no independent sample is available, could statistical random splits of the current sample be used to validate the findings? The absence of a confirmatory sample needs to be emphasized, if it cannot be overcome.
We agree with the reviewer that not being able to replicate our results in an independent sample is a limitation of our study. However, we would like to emphasize that this a fairly unique cohort, and replicating the current results in a similar cohort is therefore exceptionally difficult. For this, we have now included this in the limitations section (page 13):
“Finally, we acknowledge that replication in an independent cohort is needed to establish the robustness of our results.”

4. The denomination of the parameters reported in Table 1 is not comprehensible to the general readership and requires specifications, e.g.:
a. "Time between blood and death", authors probably mean time between plasma sampling and death / blood and brain donation.
b. "CERAD moderate / frequent", the authors probably mean CERAD neuritic plaque frequency score
c. "Braak stage", the authors probably mean Braak and Braak stages of Tau-NFT stages.
d. "CAA", probably Thal stages
e. LBD, TDP, CWMR, AGD: the table needs to be self-explaining without a deep dive into the methods section, presently the table is incomprehensible.
f. pTau, a-beta, GFAP, Nf: Plasma concentrations
g. propose to have subheadings: demongraphic data / neuropathological data / plasma biomarker data
h. Please provide specific and comprehensible information for all parameters, provide references for the neuropath staging systems applied.
We have improved Table 1 to make it more comprehensible after reviewer’s suggestions.

5. Table 2: please also provide a correlation analysis for the neuropathological plaque and tangle pathology in your sample. If factors A (e.g. plaques) and B (e.g. tangles) are significantly correlated with one another, then other factors Ci (e.g. p-tau181, p-tau217) causally related to

A can also correlate with B in that particular sample irrespective of a causal association (indirect association). This is a conceptual aspect in the current study which requires adequate attention.

We agree with the reviewer with this comment and that is exactly the reason why we performed the subsequent analysis including both measures of pathology in the same model to understand the *specific* associations of these biomarkers with each particular pathology. However, we agree that showing the association between amyloid and tau pathologies may be of interest to have a deeper understanding of why we are doing this analysis so we now included this in the manuscript (Supplementary Figure 1, see below).

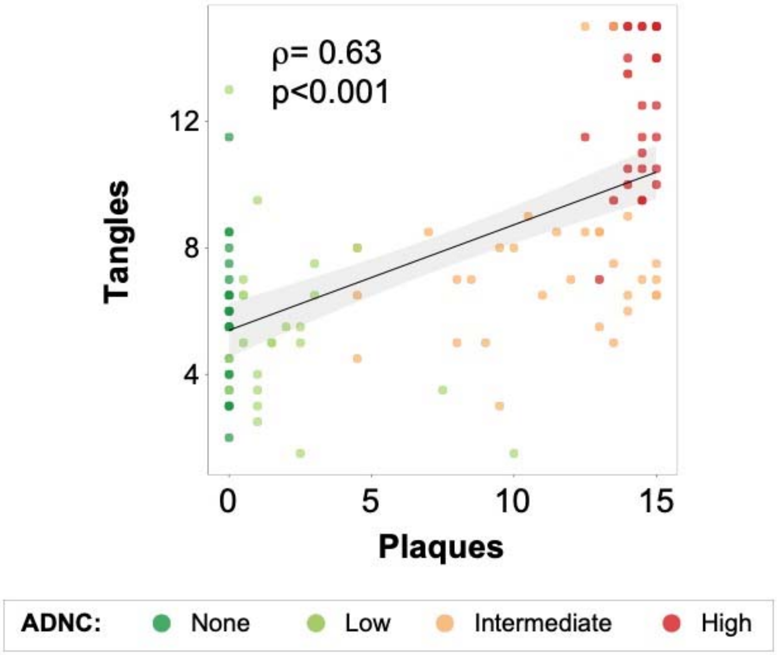

Further, we elaborated on this this analysis on page 7 of the manuscript:
"Since plaque and tangle load were highly correlated (ρ[95%CI]=0.63[0.48, 0.73], $p<0.001$, Supplementary Figure 1), we performed an analysis to identify the specific (or independent) associations between each plasma biomarker and the two pathologies."

6. While all analyses are correlative in nature, the authors implicitly suggest causal relationships between neuropathological parameters and plasma biomarker levels, e.g. heading of Figure legend 2. The authors need to interpret their correlative data in one single sample with due care. Interpretations suggesting causal relationship need to be avoided.

As the reviewer correctly noted correlation does not mean causation. Consequently we have changed Figure 2 heading and have adjusted our wording throughout manuscript.

"Figure 2 Specific associations between plasma levels and both amyloid plaque and tau tangle loads"

7. Recent evidence suggests that microglial activation is involved in the spread of tau tangles over the neocortex in Alzheimer's disease (AD) and an interaction between Aβ and activated microglia sets the pace for tau spread across Braak stages (Pascoal et al, 2021,

doi:10.1038/s41591-021-01456-w). Why did the colleagues not further check for neuroinflammation?
We agree with the reviewer that this comparison would be of utmost interest to the field. Unfortunately, these kind of data are currently not available in this dataset. We have included this as a limitation in the appropriate section (pages 13-14):

"Another plausible hypothesis is that plasma GFAP is not directly related to either plaque or tangle deposition, but rather to astrocytic reactivity in response to these pathological processes. Actually, GFAP as a protein is overexpressed in reactive astrocytes and, its measures in CSF GFAP have been widely accepted as marker of reactive astrogliosis. Unfortunately, no measures neuropathological measures of astrocytic reactivity were available in this sample, which prevented us to investigate this important issue. Future studies should investigate whether plasma GFAP is related to astrocytic reactivity and up to what level this is also indirectly related to amyloid and/or tau pathologies."

8. Another important issue concerns comparisons to other neurodegenerative diseases, which is missing in the current work. Thus, the conclusion of specificity of the associations in the last paragraph of the discussion needs to be tempered down.
We respectfully disagree with the reviewer on this point. First, the sample of this study comprised a very diverse sample in terms of neurological diagnoses including a large amount of non-AD dementias (n=42), including participants with Pick's disease and other frontotemporal dementia variants, parkinsonism or vascular dementia, among others. These participants have been included in all analyses, without taking into account their diagnosis and, therefore our results are not only focused on AD. Further, we also investigated whether any of the plasma biomarkers was able to predict the presence of other co-pathologies, such as Lewy bodies or TDP-43. Here, we found that only NfL levels were predictive of presence of cerebral white matter rarefactions. Thus, looking at comparisons to other neuropathologic findings. Thus, we consider that our conclusions are supportive of the work presented. Nonetheless we have moderated our first conclusion following reviewer's comment (14).

"In conclusion, our results support that plasma p-tau217 and plasma p-tau181 are specific markers of both amyloid plaques and tau tangles, whereas the Aβ42/40 ratio and p-tau231 levels are markers strictly associated with plaques and GFAP with tangles."

9. The colleagues discuss the role of p-tau231, both as a CSF and a plasma biomarker, as a marker for early AD stages and associated amyloid pathology (Ashton et al, 2021; Meyer et al, 2022; Milà-Alomà, 2022; Smirnov et al, 2022; Suárez-calvet et al, 2020). Why was p-Tau 231 not included in the longitudinal investigation? The investigation of earlier blood samples might enhance understanding of p-Tau231 as a possible early marker of AD.
We agree with the reviewer, and we would have liked to include all the plasma biomarkers in the longitudinal analysis, however these samples were only available for p-tau217 and p-tau181, as these were the only ones measured in-house.
We have included a brief mention to this in the methods section (page 17):
"Longitudinal samples were only analysed for p-tau217 and p-tau181, and not the Elecsys measurements or p-tau231 for logistic reasons."
And in the limitations (page 16):
"Also, we could only analyse p-tau217 and p-tau181 in this longitudinal sample, which did not allow a complete comparison among biomarkers"

Minor issues:

- Page last but one paragraph: wrong word order: ... importance the use of high performing assays is of utmost ...
- Figure 1: indication of the same parameters to the left AND at the top of the panels is redundant and confusing. The plaque and tangle unit (range 0-15) requires an explanation on the figure / in the legend, to enable the reader understanding without diving deep into the methods section.
- Figure 2: legend indicated plaques being green, tangles being orange, but figure shows blue and brown
- Suppl. Figures: the abbreviations seem to be partly inappropriate, e.g. CAA is not on display. CERAD does not refer to the consortium, but the corresponding staging system. The general reader needs to be told that CERAD and Braak classifications relate to tau and amyloid beta, NFT and plaque pathology, respectively.

We thank the reviewer for their thoughtful review. We have solved these minor issues in the current version of the manuscript.

Referee #2 (Remarks for Author):

In this neuropathological validation study of Alzheimer blood biomarkers in approx. 100 cases of the Arizona Brain Bank Salvado et al demonstrate a continuous relationship between 181phosphotau and 217phosphotau measures and amyloid plaque load as well as neurofibrillary tangle load. A strong relationship was also found for Aβ42/40 and amyloid plaque load. As a further novelty, the authors demonstrate a relationship between GFAP and neurofibrillary tangle load. The data were also analysed using a classification approach and confirm the diagnostic validity of 217phosphotau (plus Aβ42/40, see below) against an AD diagnosis based on CERAD or NIA-AA criteria. GFAP shows a surprisingly high diagnostic accuracy against Braak stageing. Overall NFL performs poorly as a diagnostic tool for detecting AD pathological changes. It is also of interest that the authors compared between each of the AD stages, e.g. none vs low neuritic plaque load, and observed significant differences for some of the biomarkers.
Needless to say, this type of neuropathological validation is essential and a critical step for research and clinical use of blood biomarkers. The data constitute a central piece of evidence, complementing prior studies that were mostly based on validation against molecular imaging biomarkers. The study design is relatively standard. There is a relatively long interval between blood sampling and neuropathological verification but this is hard (although not entirely impossible) to avoid outside the context of industry-sponsored trials. The analysis is conducted in a careful, detailed and rigorous manner and the results speak for themselves. They are comprehensively illustrated by a set of clear figures. This may become a landmark paper as far as this is predictable.

We appreciate the kind words of the reviewer about our work.

Major comment
1. It would be of interest to also report whether any of the biomarkers discriminate between CAA without vs with AD parenchymal aggregates. This is of clinical relevance and would also shed further insight in the neuropathological basis for the plasma biomarkers. Likewise for Lewy body pathology with vs without AD neuropathology.
In our previous analysis, we tried to assess whether presence of co-pathologies could be predicted with the use of plasma biomarkers regardless of their AD pathology status (adjusting for dichotomized ADNC). However, we agree with the reviewer that being able to distinguish participants with only AD pathology to those with AD pathology and other co-pathologies can

be of clinical interest. Unfortunately, due to our limited sample size, these analyses are not sufficiently powered. For this reason we decided to include this as a supplementary analysis, because it is indeed an important topic. Interestingly, we found that those participants with both CAA and AD pathologies had significantly higher p-tau217 levels than those only with AD pathology, even when correcting for multiple comparisons (FDR). However, we have to note that the AD only pathology group was very small (n=8), which prevents us to make any substantial claims on this regard, and we consider this a hypothesis generating analysis that needs follow-up in a more suitable cohort with a larger sample size.
We have included this in the results section (page 9):

"As an additional analysis, we further checked whether there were differences between plasma levels in participants with only co-pathologies (e.g, CAA only) and participants with AD pathology and co-pathologies (e.g., CAA and ADNC) as it may have clinical implications. We found that p-tau217 was significantly higher on those participants having both AD pathology (as ADNC intermediate or high) and CAA than those only with AD pathology (p=0.037, Supplementary Figure 11). However, we must note that the group of pure AD pathology was small (n=8). At the trend level, we also found differences in plasma Aβ42/40 levels in AD only vs AD and LBD groups (p=0.058, Supplementary Figure 12); in both p-tau217 and Aβ42/40 levels in AD only vs AD and AGD groups (p=0.052 and p=0.069, respectively; Supplementary Figure 13) and in NfL levels in AD only vs AD and CWMR (p=0.090, Supplementary Figure 14). No differences were observed in the case of TDP-43 (Supplementary Figure 15)."

And in the discussion (page 15):
"Interestingly, we found that participants with AD and CAA pathologies had significant higher levels of p-tau217 than those with only CAA or only AD pathology. However, due to the low number of subjects with only AD pathology, we consider this as a hypothesis generating result that needs confirmation in a larger sample."

Supplementary Figure 11:

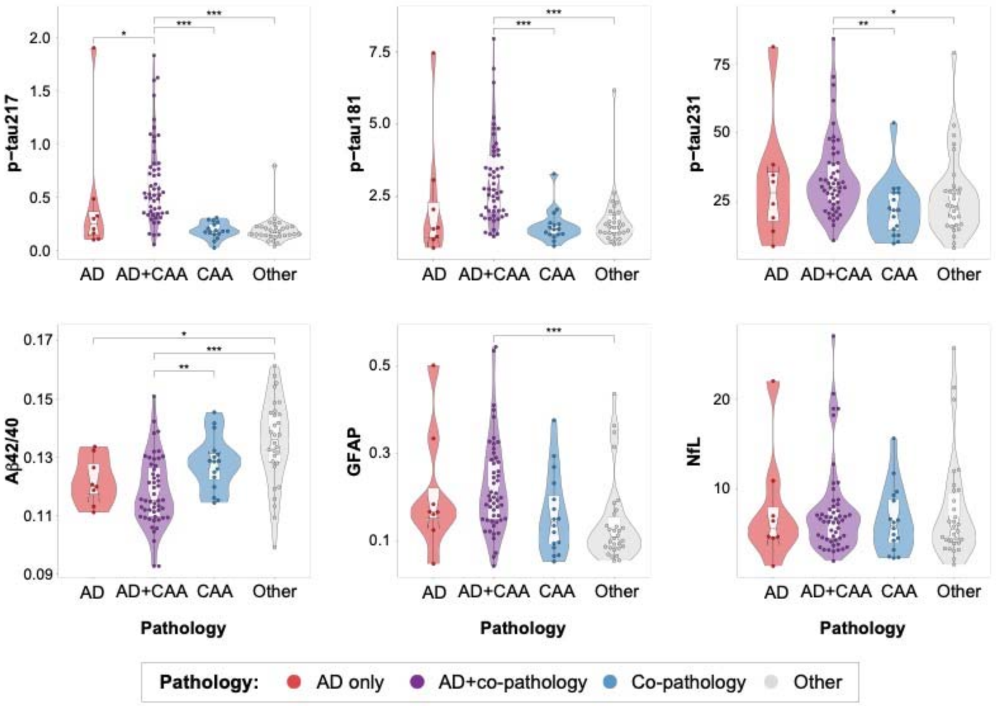

2. P 11 top paragraph: the interpretation that 217phosphotau is the best biomarker should be qualified, as this may also depend on the exact assay used for 181phosphotau. The authors may want to add a qualification in the sense of 'for the assays tested in the current study'. They do so in one of the subsequent paragraphs.

The reviewer is right that we should emphasize that our results can only be applied to the assays tested in our study.

We have included this in the Discussion (page 11):

"Taken altogether, this study supports the use of plasma p-tau217, when assessed with high-performing assays, as the best biomarker for measuring AD-related pathology, supported by its independent associations with neuropathological measures of both plaques and tangles."

3. The interpretation of the relationship between GFAP and tangle load: the authors may want to discuss the possibility of an indirect (eg as a microglial or astrocytic marker) vs a direct relationship.

We agree that this is an important point and should be included in the discussion. The following potential explanation has been added (page 13):

"Another plausible hypothesis is that plasma GFAP is not directly related to either plaque or tangle deposition, but to the astrocytic reactivity in response to these processes. Actually, GFAP as a protein is overexpressed in reactive astrocytes and, its measures in CSF GFAP have been widely accepted as marker of reactive astrogliosis. Unfortunately, no measures neuropathological measures of astrocytic reactivity were available in this sample, which prevented us to investigate this important issue. Future studies should investigate whether plasma GFAP is related to astrocytic reactivity and up to what level this is also indirectly related to amyloid and/or tau pathologies."

4. Table 4: for the longitudinal samples, the beta coefficients are relatively low and the difference between 217phosphotau and 181phosphotau small, at least for the interaction with tangle load and the interaction with ADNC. This contrasts with the relatively strong conclusions in the discussion that the former is superior for tracking disease progression in trials. The design of the longitudinal study does not allow strong conclusions and the differences between 217phosphotau and 181phosphotau are overstated. The authors may consider to remove this component as the strength of evidence is substantially weaker than for the other components of the paper.

We understand reviewer's comment, and we have modified the text to temper the conclusions of our results. We understand that these results should be taken with caution as the number of participants, among other factors, may have limited our power to also detect changes in p-tau181. However, while fully acknowledging the exploratory nature of this analysis, we think that it is important to show and discuss these results to foster other groups on trying to replicate them and advance into a deeper knowledge about plasma biomarkers trajectories over time based on pathology classification.

We have included some comments across results and discussion sections to lower our claims of these results.

Results (page 10):

"Finally, we investigated whether longitudinal changes in plasma p-tau217 and p-tau181 were associated with presence of AD pathology at death (median[range] timepoints: 2[2-5], mean(SD) time difference from first timepoint to death: 1378(1357) days). However, and given the low number of the participants included, we interpret this analysis as exploratory. Details of these participants can be found in Supplementary Table 13. First, we observed that longitudinal increments of p-tau217 but not p-tau181 were associated with plaque burden (p-tau217: β=0.09, p=0.005; p-tau181: β=0.05, p=0.350, Table 4). In independent models, we

observed that p-tau217 increments, but not those in p-tau181, were also associated with tangle load (p-tau217: β=0.09, p=0.004; p-tau181: β=0.08, p=0.094, Table 4). This result remained when removing two cases with very high plasma levels."

Discussion (page 11):
"Finally, we observed that longitudinal increases of p-tau217, but not those of p-tau181, were significantly associated with presence of AD pathology at death, especially with tangle burden. Although this analysis was exploratory, due to the limited sample size with longitudinal data, it is in agreement with a very recent study in which plasma p-tau217 was the only biomarker with significant different longitudinal increases based on amyloid status in both CU and MCI participants (Ashton et al, 2022a)."

Discussion (page 14):
"Another limitation is the restricted number of participants in the longitudinal subsample, which may have reduced the power to find significant time interactions with plasma p-tau181. In this analysis, the big difference in number of blood draws as well as their time lags may have also affected our results. Thus, our results on this regard should be taken with caution."

Minor comments:
1. Based on figure 4, the parsimonious model seems equivalent to the model with 217phosphotau alone, and there does not seem to be significant added value of combining with Abeta42/40. While numerically the parsimonious model has the highest accuracy, the difference with 217phophotau alone seems negligible. The authors argue that there is a difference in AIC but the superiority of the parsimonious model vs the 217phosphotau alone could be tested with DeLong test and it would be surprising if there is a significant difference. The difference in AIC reported on p 9 also seems very small.
As the reviewer correctly points, these two models were not significantly different when using the DeLong test, as we already commented in the previous version of the manuscript (end page 8, beginning page 9). We have now further modified the discussion to clarify this result (page 12):

"The most important finding regarding the plasma Aβ42/40 ratio was that combining it with plasma p-tau217 could slightly improve amyloid plaque assessment, replicating a previous result from our group when assessing amyloid positivity by CSF (Janelidze et al, 2022b), however in our case this improvement was not significant. Thus, our results suggest that the combination of plasma Aβ42/40 ratio and p-tau217 may be useful in clinical trials targeting amyloid pathology as a pre-screening method, but more powered studies are needed to confirm it."

Very minor comment:
1. In figure 3 I suggest to drop the figure titles since it may be confusing whether the title belongs to the figure above or below, it is clear enough from the y axis title what is plotted.
Thanks for noticing this, we have modified the figure accordingly.

Referee #3 (Remarks for Author):

The authors investigate the association between dementia-related plasma markers and pathology at postmortem in patients with low to high ADNC. They describe that specific plasma markers are associated with only amyloid (Aβ42/40 and p-tau231), only tau pathology (plasma GFAP) and, some with both pathologies (p-tau217 and p-tau181). They suggest that the combination of ptau217 and the Aβ42/40 ratio may be useful to predict amyloid

pathology, while p-tau217 alone may be a good marker to detect tau pathology.

Studies on plasma markers in dementia have become increasingly important to validate new tools that could be implemented in clinical practice and trials. The research question is very important and the dataset includes patients with different levels of ADNC severity. However, the analyses may not support the conclusions of the study completely.

The main result of the paper is based on linear regression models between plasma marker levels and plaque and tangle severity at postmortem (Figure 2 and Table 2). However, plaque and tangle loads are obtained by descriptive measures (or as stated by the authors "semi-quantitative measures") of severity that were converted into 0-3 scores in each region and then added across the 5 regions investigated. Thus, the pathology loads derive from an ordinal qualitative assessment, rather than a quantitative "continuous" evaluation (i.e. counting, AI quantification). This makes the statistical approach applied (linear regression) not appropriate for the data and research question. Indeed, the obtained variables are not normally distributed, and the distribution of data points in Figure 1, especially for plaques, is highly likely to lead to spurious correlations - partially due to the combination of groups that differ in the variables of interest (i.e. none/low vs intermediate/high ADNC). If the results of these analyses are excluded, the novelty of the work decreases significantly.
Thanks for raising this important point. We have addressed this in the current version of the manuscript using partial Spearman's ρ, which is a non-parametric test, and the main results remain very similar. In particular, all plasma biomarkers except NfL show significant associations both with amyloid plaque and tau tangle loads when assessed in independent models. For assessing plaque and tangle loads contributions on plasma levels, we used partial Spearman's ρ adjusting for the other pathology. In this case, the proportion of variance explained for each pathology changed slightly more. However, the main result, which was which main pathologies explained the major part of the variance of each biomarker, remained untouched. This meaning that both plaque and tangle loads explained a similar proportion of the variance of p-tau217 and p-tau181, whereas mainly plaques (Aβ42/40 and p-tau231) or tangles (GFAP) explained most of the variance in the other biomarkers, as previously shown with the linear regression models. Therefore, we could ascertain that our results are robust and consistent.

All results and figures have been changed across the manuscript according to the new methodological approach.

Another potentially novel part of the paper is the use of longitudinal plasma markers to predict pathology. The authors applied a linear mixed effect model with "time" as predictor, however, here it should be clarified that the intercept should be fixed on the date of death rather than the first blood sample available. As presented by the authors in Supp Figure 10, time is not the simple interval between blood samples but the time interval between each visit and death. The interval between visits and the number of samples available for each individual seem to vary a lot between participants (Supplementary Table 13: Time points, median[range] 2 [2-5]; Time difference, days, mean(SD) 1,378 (1,357)). Most of the participants are III/IV Braak stage (83.3%). These are all factors that should be discussed in the interpretation of the results. The authors describe a significant association between longitudinal changes of p-tau217 and presence of ADNC at death. However, upon visual inspection of the data in Supp Figure 10, this may be driven by two outliers (left panel, top right), and by a subsample of participants with longer intervals between visits.
We agree with the reviewer that this analysis has lower power compared to other analyses conducted with the full sample. Therefore, we acknowledge that it should be interpreted as an exploratory analysis rather than a confirmatory analysis. Please note that the intercept in our

analysis was indeed already put at time of death (and not at time of first blood draw). We have modified the figure caption to make it clearer.
"Intercept was fixed at time of death."

Further, we acknowledge the presence of a two potentially influential outliers. However, we have repeated the analyses without these two cases and p-tau217 longitudinal rates of change were still significantly different in those with significant AD pathology than those without (even with a stronger coefficient, β=0.21, p=0.009), whereas p-tau181 levels were not (β=0.16, p=0.118).

We have included some comments across results and discussion sections to lower our claims of these results.
Results (page 10):
"Finally, we investigated whether longitudinal changes in plasma p-tau217 and p-tau181 were associated with presence of AD pathology at death (median[range] timepoints: 2[2-5], mean(SD) time difference from first timepoint to death: 1378(1357) days). However, and given the low number of the participants included, we interpret this analysis as exploratory. Details of these participants can be found in Supplementary Table 13. First, we observed that longitudinal increments of p-tau217 but not p-tau181 were associated with plaque burden (p-tau217: β=0.09, p=0.005; p-tau181: β=0.05, p=0.350, Table 4). In independent models, we observed that p-tau217 increments, but not those in p-tau181, were also associated with tangle load (p-tau217: β=0.09, p=0.004; p-tau181: β=0.08, p=0.094, Table 4). This result remained when removing two cases with very high plasma levels."

Discussion (page 11):
"Finally, we observed that longitudinal increases of p-tau217, but not those of p-tau181, were significantly associated with presence of AD pathology at death, especially with tangle burden. Although this analysis was exploratory, due to the limited sample size with longitudinal data, it is in agreement with a very recent study in which plasma p-tau217 was the only biomarker with significant different longitudinal increases based on amyloid status in both CU and MCI participants (Ashton et al, 2022a)."

Discussion (page 14):
"Another limitation is the restricted number of participants in the longitudinal subsample, which may have reduced the power to find significant time interactions with plasma p-tau181. In this analysis, the big difference in number of blood draws as well as their time lags may have also affected our results. Thus, our results on this regard should be taken with caution."

Other comments:

Information on whether pathology evaluation was performed by multiple experts is not reported, and potential inter-rater agreement or limitations are not tested or described.
We have followed reviewer's suggestion and included this information in the methods section (page 16):
"All neuropathological measures were performed by a single US certified neuropathologist (TB)."

The results are based on multiple comparisons between different plasma markers and their relationship with pathology, but corrections for this have not been applied.
We agree with the reviewer and in this new version we have applied FDR correction to all our analyses. We have included this in the methods section (page 17):
"Significance was set at p<0.05 (two-tailed), corrected for multiple comparisons using false

discovery rate (FDR).”

Correlations were found across the whole ADNC spectrum, but it is not clear whether the associations of tangles and plaques with blood markers differ within ADNC specific groups?
We have investigated this aspect after reviewer’s comment. As the sample size was relatively small, we could only perform these correlations in two groups (ADNC none/low and ADNC intermediate high) to maintain sufficient statistical power. We found that the only plasma biomarker associated with amyloid plaque loads on ADNC none/low group was the Aβ42/40 ratio (ρ=-0.33, p<0.001), suggesting that this marker may be related to amyloid loads even when the total amount of pathology is very low. No biomarker showed significant associations with tangle loads in this group. When looking at the ADNC intermediate/high group, we found that the association between amyloid plaque loads and p-tau217 became significant (ρ=0.41, p=0.049) with a correlation coefficient slightly higher than that of the Aβ42/40 ratio (ρ=-0.30, p<0.001). Regarding tau pathology in this group, p-tau217 (ρ=0.56, p=0.001), p-tau181 (ρ=0.49, p=0.008) and GFAP (ρ=0.47, p=0.011) showed significant positive associations. We have included this as sensitivity analysis (page 7).

“As a sensitivity analysis, we also investigated these correlations in groups without (ADNC none or low) and with significant (ADNC intermediate or high) separately (Supplementary Table 1). In the group without significant AD pathology, only the Aβ42/40 ratio showed a significant correlation with amyloid (ρ =-0.33, p<0.001). No plasma biomarkers showed a significant correlation with tau tangle loads in this group. In the group of significant AD pathology, both p-tau217 (ρ =0.41, p=0.049) and the Aβ42/40 ratio (ρ =-0.30, p<0.001) presented a significant correlation with amyloid plaque load. Further, p-tau217 (ρ=0.56, p=0.001), p-tau181 (ρ =0.49, p=0.008) and GFAP (ρ =0.47, p=0.011) had a significant correlation with tau tangle load.”

ADNC negative/positive groups were defined by pathology examination. Did these reflect AD-biomarker positivity/negativity in vivo, especially in MCI?
The gold standard of measuring presence of Alzheimer’s disease pathology is with the use of neuropathological examination. For this reason, we used dichotomized ADNC as to define presence of pathology and not the reverse. However, we have calculated this comparison after reviewer’s suggestion. We used a previously derived p-tau217 cut-off based on the mean and SD (cut-off=0.208) of a group of amyloid negative controls from an independent sample. When using this cut-off we observed a good accuracy (acc=0.86, sensitivity=0.83, specificity=0.89, see attached table) in the whole cohort. Notably, all MCI patients (n=8) were correctly categorized with this cut-off (TP=4, TN=4).

	p-tau217 negative	p-tau217 positive
ADNC none/low	41	5
ADNC interm./high	10	49

Other pathologies have been investigated in post-mortem examination (i.e. CAA, TDP-43, LBD and AGD). Were primary tauopathies such as CBD, PSP, Pick's disease not considered given that participants with AD, MCI and other neurodegenerative diseases were described in the method section?

We thank the reviewer for highlighting this point. In a sensitivity analyses we have now examined whether there were any differences between subjects with only AD pathology, from those with other tauopathies (CBD, PSP and AGD) or mixed pathologies (AD+tauopathy). Interestingly, p-tau217, p-tau181 the Aβ42/40 ratio and GFAP levels from those with only AD pathology were significantly different than from those with only primary tauopathies, as these had similar levels as subjects without any of these pathologies. However, only Aβ42/40 ratio levels were also significantly different from those participants with only AD pathology than those that also had tauopathies, although their CERAD classification was not different.
We have included this in the results section (page 10):
"Finally, we also considered primary tauopathies (CBD, PSP and AGD) as a unique group and compared plasma levels of those participants to those with only AD pathology and those with AD pathology and other tauopathies. Plasma p-tau217 ($p<0.001$), p-tau181 ($p=0.001$), Ab42/40 ratio ($p<0.001$) and GFAP ($p=0.024$) levels were significantly different when comparing participants with only AD pathology and participants with only primary tauopathies (Supplementary Figure 16). Only Ab42/40 ratio levels were different between AD only and the AD group with CBD, PSP or AGD pathology ($p=0.038$)."

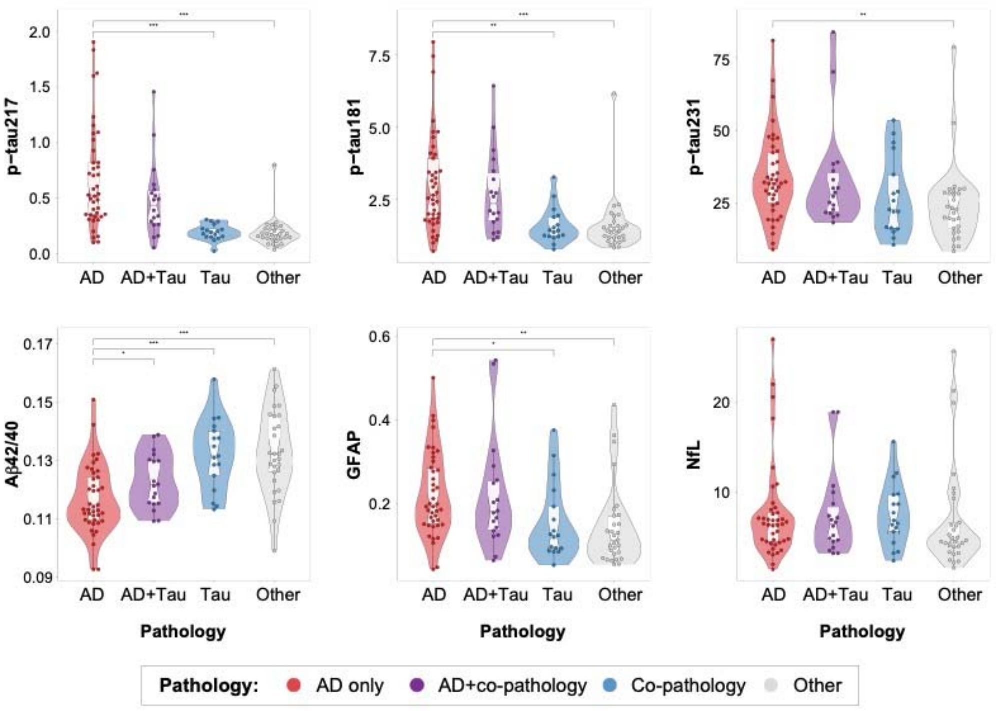

In Table 2, parameters for other factors included in the model should be added (ie age sex etc).
Following reviewer's previous comment, we have now used partial Spearman's in our analyses instead of linear regression models. Therefore, we cannot include any other factor in the table as we are only controlling for them, but no correlation factors are calculated.

23rd Jan 2023

Dear Dr. Hansson,

Thank you for the submission of your revised manuscript to EMBO Molecular Medicine. We have now received the enclosed reports from the three referees who agreed to re-assess it. As you will see, the referees are now overall supportive, and I am pleased to inform you that we will be able to accept your manuscript pending the following amendments:

1. Please address the remaining minor concerns of Referees #2 and #3.

2. On a more editorial level:

***** Reviewer's comments *****

Referee #1 (Comments on Novelty/Model System for Author):

high quality in-vivo-biospecimen vs. postmortem chohort.

Referee #2 (Comments on Novelty/Model System for Author):

see my previous review

Referee #2 (Remarks for Author):

In response to minor comment 1 of rev 2, the authors have added a paragraph to the discussion. The paragraph should end at 'was not significant'. If it is not significant, it is inconsequential to conclude that it suggests that adding Abeta42/40 helps in clinical trial design as much more robust evidence is needed for that purpose.
In response to major comment 4 reviewer 2 and a similar comment by reviewer 3, the authors nonetheless decided to keep the correlation analysis between longitudinal change in biomarker assay values and neuropathology and the interpretation that this proved superiority of phosphotau217. Scientifically, it is not enough to show that the correlation is significant for one regression analysis and not for the other. The authors should test whether the correlation differs significantly between the two analyses. If that is not the case, there is no scientific basis from the current data for the conclusion that phosphotau217 is superior

Referee #3 (Remarks for Author):

The authors have satisfactorily addressed my comments.

Since the last revision, the authors may want to discuss their findings as compared to the recently published ones by Murray et al https://pubmed.ncbi.nlm.nih.gov/36575455/, which assessed the relationship between ptau blood markers and post-mortem pathology features.

Referee #2 (Remarks for Author):

In response to minor comment 1 of rev 2, the authors have added a paragraph to the discussion. The paragraph should end at 'was not significant'. If it is not significant, it is inconsequential to conclude that it suggests that adding Abeta42/40 helps in clinical trial design as much more robust evidence is needed for that purpose.

Authors' response: We thank the reviewer for pointing this out as there was an error in our explanation. The addition of plasma Ab42/40 improves the model for predicting the continuous measure of plaques, but not the dichotomization of the CERAD scale. This was already explained in the results but not in the discussion. Thus, we have modified the discussion appropriately.

"Nonetheless, the most important finding regarding the plasma Aβ42/40 ratio was that combining it with plasma p-tau217 might slightly improve amyloid plaque assessment. This is a replication of a previous result from our group when assessing amyloid positivity by CSF (Janelidze et al, 2022b), although it should be mentioned that in our study this improvement only reached significance when predicting the continuous variable. Thus, our results suggest that the combination of plasma Aβ42/40 ratio and p-tau217 may be useful in clinical trials targeting amyloid pathology as a pre-screening method, but more powered studies are needed to confirm the additional value of the Aβ42/40 ratio."

In response to major comment 4 reviewer 2 and a similar comment by reviewer 3, the authors nonetheless decided to keep the correlation analysis between longitudinal change in biomarker assay values and neuropathology and the interpretation that this proved superiority of phosphotau217. Scientifically, it is not enough to show that the correlation is significant for one regression analysis and not for the other. The authors should test whether the correlation differs significantly between the two analyses. If that is not the case, there is no scientific basis from the current data for the conclusion that phosphotau217 is superior

Authors' response: We agree with the reviewer that to interpret the difference in association between two markers we should compare them statistically. We have now performed this comparison and found no significant differences, as expected. However, we would like to highlight that when we argued that p-tau217 may be a better biomarker for pathology rather than p-tau181, we took into account the whole set of analyses and not only the longitudinal results. To clarify this, we have included a few sentences in the new version of the manuscript. To address this important distinction, we have added the new analysis to the results, and further elaborated on its interpretation in the discussion.

Results:
"Notably, none of the correlation coefficients of p-tau217 were significantly different than those of p-tau181 ($p \geq 0.447$), when comparing them using bootstrapping."

Discussion:
"The novelties of our study were to demonstrate that this dual association only occurred in p-tau217 and p-tau181. Further, we observed that p-tau217 changed earlier along the ADNC scale (Figure 3). And also that longitudinal changes in plasma p-tau217, but not those of p-tau181, were associated with AD-related pathology, although their coefficients were not statistically different. Thus, these analyses should be repeated in a larger sample size to be able to accurately compare both biomarkers longitudinally, as has been recently done with imaging outcomes (Ashton et al, 2023). Although p-tau181 has shown very good performance as an AD biomarker (Karikari et al, 2020a; Thijssen et al, 2020; Karikari et al, 2021; Grothe et al,

2021; Janelidze et al, 2020a), multiple (plasma and CSF) studies support that p-tau217 may be a more useful biomarker than p-tau181, as it has stronger correlations with amyloid and tau pathology proxies, earlier change, and better diagnostic accuracy (Barthélemy et al, 2020; Janelidze et al, 2020b, 2021a; Hanes et al, 2020; Grothe et al, 2021; Palmqvist et al, 2020; Leuzy et al, 2021). Altogether, our and previous data suggest that plasma p-tau217 is the best suited plasma biomarker among the ones studied here to assess presence of AD-related pathology across the whole continuum. Further, our longitudinal results suggest that the utilization of plasma p-tau217 in clinical trials may be useful not only as a pre-screening method, but also for disease monitoring, especially for those drugs targeting tau pathology Importantly, larger sample sizes are needed to confirm this finding in future studies."

Referee #3 (Remarks for Author):

The authors have satisfactorily addressed my comments.

Since the last revision, the authors may want to discuss their findings as compared to the recently published ones by Murray et al https://pubmed.ncbi.nlm.nih.gov/36575455/, which assessed the relationship between ptau blood markers and post-mortem pathology features.

Authors' response: We agree with the reviewer that this work support some of our results and needs to be discussed in our manuscript. We have included a mention in our discussion.

Discussion (page 12):
"The main result of this study was the observation that plasma p-tau217 and plasma p-tau181 were specific markers of both amyloid plaques and tau tangles. A previous study with a subsample of the individuals included here (n=88) already suggested an independent association between plasma p-tau217 and the two main AD-related pathologies (Mattsson-Carlgren et al, 2021). Also, a very recent independent study showed similar results, where both amyloid and tau pathologies were associated with plasma p-tau217 and p-tau181 (Murray et al, 2022)."

(page 13):
"Although p-tau181 has shown very good performance as an AD biomarker (Karikari et al, 2020a; Thijssen et al, 2020; Karikari et al, 2021; Grothe et al, 2021; Janelidze et al, 2020a), multiple (plasma and CSF) studies support that p-tau217 may be a more useful biomarker than p-tau181, as it has stronger correlations with amyloid and tau pathology proxies, earlier change, and better diagnostic accuracy (Barthélemy et al, 2020; Janelidze et al, 2020b, 2021a; Hanes et al, 2020; Grothe et al, 2021; Palmqvist et al, 2020; Leuzy et al, 2021; Murray et al, 2022). Altogether, our and previous data suggest that plasma p-tau217 is the best suited plasma biomarker among the ones studied here to assess presence of AD-related pathology across the whole continuum."

21st Feb 2023

Dear Oskar,

We are pleased to inform you that your manuscript is accepted for publication and is now being sent to our publisher to be included in the next available issue of EMBO Molecular Medicine.

We would like to remind you that as part of the EMBO Publications transparent editorial process initiative, EMBO Molecular Medicine will publish a Review Process File online to accompany accepted manuscripts. If you do NOT want the file to be published or would like to exclude figures, please immediately inform the editorial office via e-mail.

Please read below for additional IMPORTANT information regarding your article, its publication and the production process.

Congratulations on your interesting work,

Jingyi

Jingyi Hou
Editor
EMBO Molecular Medicine

Follow us on Twitter @EmboMolMed
Sign up for eTOCs at embopress.org/alertsfeeds

*** *** *** IMPORTANT INFORMATION *** *** ***

SPEED OF PUBLICATION
The journal aims for rapid publication of papers, using using the advance online publication "Early View" to expedite the process: A properly copy-edited and formatted version will be published as "Early View" after the proofs have been corrected. Please help the Editors and publisher avoid delays by providing e-mail address(es), telephone and fax numbers at which author(s) can be contacted.

Should you be planning a Press Release on your article, please get in contact with embomolmed@wiley.com as early as possible, in order to coordinate publication and release dates.

LICENSE AND PAYMENT:

All articles published in EMBO Molecular Medicine are fully open access: immediately and freely available to read, download and share.

EMBO Molecular Medicine charges an article processing charge (APC) to cover the publication costs. You, as the corresponding author for this manuscript, should have already received a quote with the article processing fee separately. Please let us know in case this quote has not been received.

Once your article is at Wiley for editorial production you will receive an email from Wiley's Author Services system, which will ask you to log in and will present you with the publication license form for completion. Within the same system the publication fee can be paid by credit card, an invoice, pro forma invoice or purchase order can be requested.

Payment of the publication charge and the signed Open Access Agreement form must be received before the article can be published online.

PROOFS

You will receive the proofs by e-mail approximately 2 weeks after all relevant files have been sent o our Production Office. Please return them within 48 hours and if there should be any problems, please contact the production office at embopressproduction@wiley.com.

Please inform us if there is likely to be any difficulty in reaching you at the above address at that time. Failure to meet our deadlines may result in a delay of publication.

All further communications concerning your paper proofs should quote reference number EMM-2022-17123-V3 and be directed to the production office at embopressproduction@wiley.com.

Thank you,

Jingyi Hou
Editor
EMBO Molecular Medicine

EMBO Press Author Checklist

Corresponding Author Name: Oskar Hansson	
Journal Submitted to: EMBO Molecular Medicine	
Manuscript Number: EMM-2022-17123-V2	

USEFUL LINKS FOR COMPLETING THIS FORM
The EMBO Journal - Author Guidelines
EMBO Reports - Author Guidelines
Molecular Systems Biology - Author Guidelines
EMBO Molecular Medicine - Author Guidelines

Reporting Checklist for Life Science Articles (updated January 2022)
This checklist is adapted from Materials Design Analysis Reporting (MDAR) Checklist for Authors. MDAR establishes a minimum set of requirements in transparent reporting in the life sciences (see Statement of Task: 10.31222/osf.io/9sm4x). Please follow the journal's guidelines in preparing your manuscript.
Please note that a copy of this checklist will be published alongside your article.

Abridged guidelines for figures
1. Data
The data shown in figures should satisfy the following conditions:
- the data were obtained and processed according to the field's best practice and are presented to reflect the results of the experiments in an accurate and unbiased manner.
- ideally, figure panels should include only measurements that are directly comparable to each other and obtained with the same assay.
- plots include clearly labeled error bars for independent experiments and sample sizes. Unless justified, error bars should not be shown for technical replicates.
- if n<5, the individual data points from each experiment should be plotted. Any statistical test employed should be justified.
- Source Data should be included to report the data underlying figures according to the guidelines set out in the authorship guidelines on Data Presentation.

2. Captions
Each figure caption should contain the following information, for each panel where they are relevant:
- a specification of the experimental system investigated (eg cell line, species name).
- the assay(s) and method(s) used to carry out the reported observations and measurements.
- an explicit mention of the biological and chemical entity(ies) that are being measured.
- an explicit mention of the biological and chemical entity(ies) that are altered/varied/perturbed in a controlled manner.
- the exact sample size (n) for each experimental group/condition, given as a number, not a range;
- a description of the sample collection allowing the reader to understand whether the samples represent technical or biological replicates (including how many animals, litters, cultures, etc.).
- a statement of how many times the experiment shown was independently replicated in the laboratory.
- definitions of statistical methods and measures:
  - common tests, such as t-test (please specify whether paired vs. unpaired), simple χ2 tests, Wilcoxon and Mann-Whitney tests, can be unambiguously identified by name only, but more complex techniques should be described in the methods section;
  - are tests one-sided or two-sided?
  - are there adjustments for multiple comparisons?
  - exact statistical test results, e.g., P values = x but not P values < x;
  - definition of 'center values' as median or average;
  - definition of error bars as s.d. or s.e.m.

Please complete ALL of the questions below.
Select "Not Applicable" only when the requested information is not relevant for your study.

Materials

Newly Created Materials	Information included in the manuscript?	In which section is the information available? (Reagents and Tools Table, Materials and Methods, Figures, Data Availability Section)
New materials and reagents need to be available; do any restrictions apply?	Not Applicable	

Antibodies	Information included in the manuscript?	In which section is the information available? (Reagents and Tools Table, Materials and Methods, Figures, Data Availability Section)
For **antibodies** provide the following information:<br>- Commercial antibodies: RRID (if possible) or supplier name, catalogue number and or/clone number<br>- Non-commercial: RRID or citation	Not Applicable	

DNA and RNA sequences	Information included in the manuscript?	In which section is the information available? (Reagents and Tools Table, Materials and Methods, Figures, Data Availability Section)
**Short novel DNA or RNA including primers, probes**: provide the sequences.	Not Applicable	

Cell materials	Information included in the manuscript?	In which section is the information available? (Reagents and Tools Table, Materials and Methods, Figures, Data Availability Section)
**Cell lines:** Provide species information, strain. Provide accession number in repository **OR** supplier name, catalog number, clone number, and/**OR** RRID.	Not Applicable	
**Primary cultures:** Provide species, strain, sex of origin, genetic modification status.	Not Applicable	
Report if the cell lines were recently **authenticated** (e.g., by STR profiling) and tested for mycoplasma contamination.	Not Applicable	

Experimental animals	Information included in the manuscript?	In which section is the information available? (Reagents and Tools Table, Materials and Methods, Figures, Data Availability Section)
**Laboratory animals or Model organisms:** Provide species, strain, sex, age, genetic modification status. Provide accession number in repository **OR** supplier name, catalog number, clone number, **OR** RRID.	Not Applicable	
**Animal observed in or captured from the field:** Provide species, sex, and age where possible.	Not Applicable	
Please detail **housing and husbandry conditions**.	Not Applicable	

Plants and microbes	Information included in the manuscript?	In which section is the information available? (Reagents and Tools Table, Materials and Methods, Figures, Data Availability Section)
**Plants:** provide species and strain, ecotype and cultivar where relevant, unique accession number if available, and source (including location for collected wild specimens).	Not Applicable	
**Microbes:** provide species and strain, unique accession number if available, and source.	Not Applicable	

Human research participants	Information included in the manuscript?	In which section is the information available? (Reagents and Tools Table, Materials and Methods, Figures, Data Availability Section)
If collected and within the bounds of privacy constraints report on age, sex and gender or ethnicity for all study participants.	Yes	Materials and Methods

Core facilities	Information included in the manuscript?	In which section is the information available? (Reagents and Tools Table, Materials and Methods, Figures, Data Availability Section)
If your work benefited from core facilities, was their service mentioned in the acknowledgments section?	Not Applicable	

Design

Study protocol	Information included in the manuscript?	In which section is the information available? (Reagents and Tools Table, Materials and Methods, Figures, Data Availability Section)
If study protocol has been **pre-registered, provide DOI in the manuscript**. For clinical trials, provide the trial registration number **OR** cite DOI.	Not Applicable	
Report the **clinical trial registration number** (at ClinicalTrials.gov or equivalent), where applicable.	Not Applicable	

Laboratory protocol	Information included in the manuscript?	In which section is the information available? (Reagents and Tools Table, Materials and Methods, Figures, Data Availability Section)
Provide DOI OR other citation details if **external detailed step-by-step protocols** are available.	Not Applicable	

Experimental study design and statistics	Information included in the manuscript?	In which section is the information available? (Reagents and Tools Table, Materials and Methods, Figures, Data Availability Section)
Include a statement about **sample size** estimate even if no statistical methods were used.	Not Applicable	
Were any steps taken to minimize the effects of subjective bias when allocating animals/samples to treatment (e.g. **randomization procedure**)? If yes, have they been described?	Not Applicable	
Include a statement about **blinding** even if no blinding was done.	Yes	Materials and Methods
Describe **inclusion/exclusion criteria** if samples or animals were excluded from the analysis. Were the criteria pre-established?<br><br>If sample or data points were omitted from analysis, report if this was due to attrition or intentional exclusion and provide justification.	Yes	Materials and Methods
For every figure, are **statistical tests** justified as appropriate? Do the data meet the assumptions of the tests (e.g., normal distribution)? Describe any methods used to assess it. Is there an estimate of variation within each group of data? Is the variance similar between the groups that are being statistically compared?	Yes	Materials and Methods

Sample definition and in-laboratory replication	Information included in the manuscript?	In which section is the information available? (Reagents and Tools Table, Materials and Methods, Figures, Data Availability Section)
In the figure legends: state number of times the experiment was **replicated** in laboratory.	Not Applicable	
In the figure legends: define whether data describe **technical or biological replicates**.	Not Applicable	

Ethics

Ethics	Information included in the manuscript?	In which section is the information available? (Reagents and Tools Table, Materials and Methods, Figures, Data Availability Section)
Studies involving **human participants**: State details of **authority granting ethics approval** (IRB or equivalent committee(s), provide reference number for approval.	Yes	Materials and Methods
Studies involving **human participants**: Include a statement confirming that **informed consent** was obtained from all subjects and that the experiments conformed to the principles set out in the WMA Declaration of Helsinki and the Department of Health and Human Services Belmont Report.	Yes	Materials and Methods
Studies involving **human participants:** For publication of **patient photos**, include a statement confirming that consent to publish was obtained.	Not Applicable	
Studies involving experimental **animals**: State details of **authority granting ethics approval** (IRB or equivalent committee(s), provide reference number for approval. Include a statement of compliance with ethical regulations.	Not Applicable	
Studies involving **specimen and field samples:** State if relevant **permits** obtained, provide details of authority approving study; if none were required, explain why.	Not Applicable	

Dual Use Research of Concern (DURC)	Information included in the manuscript?	In which section is the information available? (Reagents and Tools Table, Materials and Methods, Figures, Data Availability Section)
Could your study fall under dual use research restrictions? Please check biosecurity documents and list of **select agents and toxins** (CDC): https://www.selectagents.gov/sat/list.htm	Not Applicable	
If you used a select agent, is the security level of the lab appropriate and reported in the manuscript?	Not Applicable	
If a study is subject to dual use research of concern regulations, is the name of the **authority granting approval and reference number** for the regulatory approval provided in the manuscript?	Not Applicable	

Reporting

The MDAR framework recommends adoption of discipline-specific guidelines, established and endorsed through community initiatives. Journals have their own policy about requiring specific guidelines and recommendations to complement MDAR.

Adherence to community standards	Information included in the manuscript?	In which section is the information available? (Reagents and Tools Table, Materials and Methods, Figures, Data Availability Section)
State if relevant guidelines or checklists (e.g., **ICMJE, MIBBI, ARRIVE, PRISMA**) have been followed or provided.	Not Applicable	
For **tumor marker prognostic studies,** we recommend that you follow the **REMARK** reporting guidelines (see link list at top right). See author guidelines, under 'Reporting Guidelines'. Please confirm you have followed these guidelines.	Not Applicable	
For **phase II and III randomized controlled trials**, please refer to the **CONSORT** flow diagram (see link list at top right) and submit the CONSORT checklist (see link list at top right) with your submission. See author guidelines, under 'Reporting Guidelines'. Please confirm you have submitted this list.	Not Applicable	

Data Availability

Data availability	Information included in the manuscript?	In which section is the information available? (Reagents and Tools Table, Materials and Methods, Figures, Data Availability Section)
Have **primary datasets** been deposited according to the journal's guidelines (see 'Data Deposition' section) and the respective accession numbers provided in the Data Availability Section?	Not Applicable	
Were **human clinical and genomic datasets** deposited in a public access-controlled repository in accordance to ethical obligations to the patients and to the applicable consent agreement?	Not Applicable	
Are **computational models** that are central and integral to a study available without restrictions in a machine-readable form? Were the relevant accession numbers or links provided?	Not Applicable	
If publicly available data were reused, provide the respective **data citations in the reference list**.	Not Applicable	
